# Erratum to “Aggravated Ulcerative Colitis via circNlgn-Mediated Suppression of Nuclear Actin Polymerization”

**DOI:** 10.34133/research.0684

**Published:** 2025-04-29

**Authors:** William W. Du, Chi Zhou, Hui Yang, Shuoyang Wen, Yu Chen, Eric X. Chen, Xiuwei H. Yang, Feiya Li, Kevin Y. Du, Hui Yuan, Ting Ye, Javeria Qadir, Burton B. Yang

**Affiliations:** ^1^ Sunnybrook Research Institute, Sunnybrook Health Sciences Centre, Toronto, ON, Canada.; ^2^ Department of Laboratory Medicine and Pathobiology, University of Toronto, Toronto, ON, Canada.; ^3^ Department of Colorectal Surgery, Sun Yat-sen University Cancer Center, Guangzhou, China.; ^4^ State Key Laboratory of Oncology in South China, Collaborative Innovation Center for Cancer Medicine, Sun Yat-sen University Cancer Center, Guangzhou, China.; ^5^ State Key Laboratory of Oncology in South China, Guangdong Provincial Clinical Research Center for Cancer, Sun Yat-sen University Cancer Center, Guangzhou, China.; ^6^ The Second Affiliated Hospital of Guangzhou Medical University, Guangzhou, China.; ^7^ Princess Margaret Cancer Centre, Toronto, ON, Canada.; ^8^ Department of Pharmacology and Nutritional Sciences, College of Medicine, University of Kentucky, Lexington, KY, USA.

In the Research Article “Aggravated Ulcerative Colitis via circNlgn-Mediated Suppression of Nuclear Actin Polymerization,” an error occurred in panel F of Figure 3 [1]. During the final submission, the authors inadvertently uploaded a version of Figure 3 with an error in panel F. The figure has now been corrected in the original version and is also presented below.

**Figure 3.**
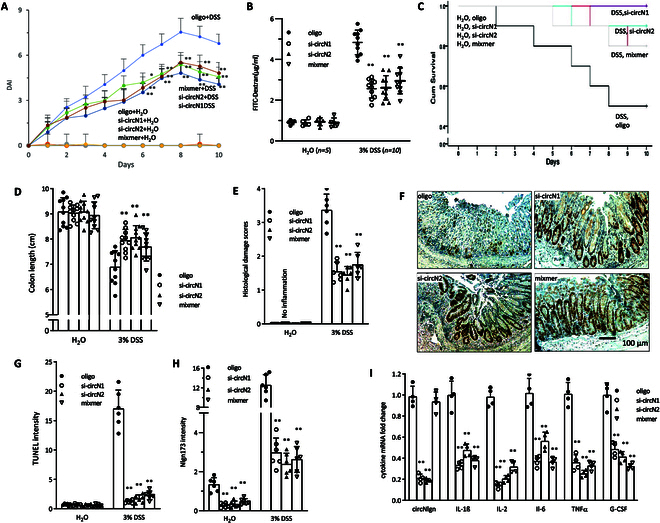
Improvement of colitis outcome by targeting Nlgn173. (A) A graph showing that silencing circNlgn with siRNAs or blocking circNlgn translation with a mixmer mitigated DSS-induced DAI increase. **P < 0.05, **P < 0.01 versus oligo (*n* = 10). (B) A graph showing that circNlgn siRNAs- or mixmer-delivered mice had lower FITC-dextran levels in the serum after DSS treatment. **P < 0.01 versus oligo (*n* = 10). (C) Mice were delivered with circNlgn siRNAs or mixmer by nanoparticles as Materials and Methods described, and administrated with 3% DSS or H_2_O. Kaplan–Meier survival test showed that silencing circNlgn with siRNAs or blocking circNlgn translation with a mixmer enhanced mouse survival in the 3% DSS-induced mouse colitis model. **P < 0.05, **P < 0.01 versus oligo (H_2_O, *n* = 5; 3% DSS, *n* = 20). (D) Left: A graph showing that silencing circNlgn with siRNAs or blocking circNlgn translation with a mixmer prevented colon shortening induced by DSS treatment. **P < 0.01 versus oligo (*n* = 10). (E) Left: A graph showing that circNlgn siRNAs- or mixmer-delivered mouse colon sections displayed lower histological damage score than control mice after DSS treatment. **P < 0.01 versus oligo (*n* = 6). (F) Immunofluorescence staining showed that circNlgn siRNAs- or mixmer-delivered mouse mucosa expressed higher Ki67 levels than control mice after DSS treatment. (G) Left: ImageJ analysis of TUNEL staining showed that circNlgn siRNAs- or mixmer-delivered mouse mucosa displayed lower TUNEL intensity than those of control mice after DSS treatment. **P < 0.01 versus oligo (*n* = 6). (H) ImageJ analysis of in situ hybridization staining showed that circNlgn siRNAs-delivered colonic mucosa expressed lower levels of Nlgn173 in the nucleic. **P < 0.01 versus oligo (*n* = 6). (I) Left: Colonic mucosa was collected and subjected to RT-PCR, showing that circNlgn siRNAs- or mixmer-delivered mouse mucosa expressed much lower levels of IL-1β, IL-2, IL-6, TNFα, and G-CSF mRNA after DSS treatment. **P < 0.05, **P < 0.01 versus oligo (*n* = 4).
