# Supplementary material for: Aggravated Ulcerative Colitis via circNlgn-Mediated Suppression of Nuclear Actin Polymerization
Source: Research (Wash D C). 2024 Aug 23;7:0441. doi: 10.34133/research.0441 (PMC11342054; doi:10.34133/research.0441)
Supplement: 20240823-1 [file research.0441.v1.pdf]

Research
A SCIENCE PARTNER JOURNAL

RESEARCH ARTICLE

Aggravated Ulcerative Colitis via circNlgn-Mediated Suppression of Nuclear Actin Polymerization

William W. Du[1,2†], Chi Zhou[1,2,3,4,5†], Hui Yang[6†], Shuoyang Wen[1,2], Yu Chen[1,2], Eric X. Chen[7], Xiuwei H. Yang[8], Feiya Li[1,2], Kevin Y. Du[1,2], Hui Yuan[1,2], Ting Ye[1,2], Javeria Qadir[1,2], and Burton B. Yang[1,2*]

[1]Sunnybrook Research Institute, Sunnybrook Health Sciences Centre, Toronto, ON, Canada. [2]Department of Laboratory Medicine and Pathobiology, University of Toronto, Toronto, ON, Canada. [3]Department of Colorectal Surgery, Sun Yat-sen University Cancer Center, Guangzhou, China. [4]State Key Laboratory of Oncology in South China, Collaborative Innovation Center for Cancer Medicine, Sun Yat-sen University Cancer Center, Guangzhou, China. [5]State Key Laboratory of Oncology in South China, Guangdong Provincial Clinical Research Center for Cancer, Sun Yat-sen University Cancer Center, Guangzhou, China. [6]The Second Affiliated Hospital of Guangzhou Medical University, Guangzhou, China. [7]Princess Margaret Cancer Centre, Toronto, ON, Canada. [8]Department of Pharmacology and Nutritional Sciences, College of Medicine, University of Kentucky, Lexington, KY, USA.

*Address correspondence to: byang@sri.utoronto.ca
†These authors contributed equally to this work.

Citation: Du WW, Zhou C, Yang H, Wen S, Chen Y, Chen EX, Yang XH, Li F, Du KY, Yuan H, et al. Aggravated Ulcerative Colitis via circNlgn-Mediated Suppression of Nuclear Actin Polymerization. *Research* 2024;7:Article 0441. https://doi.org/10.34133/research.0441

Submitted 7 June 2024
Accepted 12 July 2024
Published 23 August 2024

Copyright © 2024 William W. Du et al. Exclusive licensee Science and Technology Review Publishing House. No claim to original U.S. Government Works. Distributed under a Creative Commons Attribution License 4.0 (CC BY 4.0).

Colitis is a chronic bowel disease characterized by damage to the lining of the large intestine, with its precise underlying causes remaining incompletely understood. In this study, we provide evidence that circular RNA circNlgn plays a pivotal role in promoting the development of colitis. Colitis patients produce significant higher levels of circNlgn. Transgenic mice expressing circNlgn exhibit heightened susceptibility to colitis development and progression, primarily attributed to the presence of the protein isoform Nlgn173 encoded by circNlgn. Nlgn173 undergoes translocation into cell nuclei, where it interacts with actin, impeding the binding of actin-related protein 2 and 3 (Arp2/3) complex to actin molecules. Consequently, this leads to a reduction in actin polymerization. Mechanistically, Nlgn173 enhances tyrosine-53 phosphorylation of nuclear actin, diminishing its capacity to interact with the Arp2/3 complex and causing a decrease in filamentous actin levels. These alterations in actin dynamics result in inhibited cell cycle progression, increased apoptosis, and decreased proliferation of colonic epithelial cells, thereby exacerbating colitis development and progression. In contrast, the silencing of circNlgn or the targeted inhibition of Nlgn173 translation and nuclear translocation leads to the promotion of nuclear actin polymerization, enhanced cell survival, and reduced apoptosis and ultimately improves the outcome of colitis in vivo. Interestingly, nuclear actin polymerization is highly related with expression of PIAS3, which modulates signal transducer and activator of transcription 3 and NF-κB activity in colitis. Strategies such as circNlgn knockdown and targeting nuclear actin polymerization of the colonic epithelium may explore a novel avenue for acute ulcerative colitis clinical intervention.

Introduction

Colitis is a chronic bowel disease that predominantly affects the colon and rectum, characterized by inflammation that leads to damage in the intestinal lining. This complex ailment results from a confluence of factors, including genetic predisposition, environmental influences, the composition of the intestinal microbiota, and aberrant immune responses [1–3]. Despite substantial knowledge about these contributing elements, the precise etiology of colitis remains elusive. A vital element in maintaining the integrity of the intestinal barrier is the single-cell layer that separates the gut lumen from the underlying tissue [4,5]. Alterations in the function and structure of these epithelial cells are strongly implicated in the development of colitis. Actin, a highly conserved molecule, serves as a major component of the cytoskeleton and plays a pivotal role in regulating the dynamics of epithelial cells [6–8]. Actin dynamics encompass both polymerization and depolymerization processes in the cytoplasm and nucleus.

While actin dynamics are most commonly associated with its cytoplasmic functions, it also exerts crucial control in the

nucleus, influencing gene expression and chromatin remodeling. This influence is mediated by interactions between actin and various nuclear proteins, including transcription factors and chromatin modifiers [9–11]. Polymerization of actin in the nucleus results in the binding of actin to actin-related protein 2/3 (Arp2/3 complex), consequently enhancing processes such as gene transcription [12], DNA repair [13,14], and chromatin remodeling [15,16]. Arp2/3 complex controls actin nucleation and branching filament assembly in actin polymerization. Increased nuclear filamentous actin (F-actin) levels promote overall transcription levels [17], whereas reduced levels suppress the expression of specific genes [18]. Nuclear actin polymerization is a key regulator of various cellular processes, including tissue repair and regeneration. Upon injury or tissue damage, nuclear actin is activated to polymerize, initiating gene expression, structural remodeling, cell proliferation, migration, and differentiation, ultimately leading to tissue repair and regeneration. However, the precise mechanisms that govern the initiation of nuclear actin polymerization remain enigmatic.

In this study, we present evidence that circular RNA (circRNA) circNlgn plays roles in the modulation of nuclear actin polymerization. Our investigation involved the profiling of circRNA expression in human colitis samples, with circNlgn emerging as one of the most significantly up-regulated circRNAs. Building on our prior research, which demonstrated the translation of circNlgn and the translocation of the encoded protein Nlgn173 into cell nuclei [19,20], we now provide further insights into the role of Nlgn173 in decreasing nuclear actin polymerization and impairing epithelial renewal.

Results

Expression of circNlgn in acute ulcerative colitis

High-throughput circRNA sequencing was conducted on colon samples obtained from patients diagnosed with colitis. In total, 26,251 circRNAs were identified in these samples, with each circRNA supported by at least 2 reads spanning a head-to-tail splice junction in every sample. When compared to control tissues, 232 circRNAs exhibited significant differential expression, exceeding a 2-fold threshold (Fig. 1A), while 104 circRNAs exceeded a 10-fold threshold. Among these, circNlgn emerged as one of the most prominently up-regulated circRNAs. In a previous study, we elucidated that circNlgn is translated into an encoded protein, Nlgn173, which is translocated to cell nuclei, contributing to cardiac remodeling and fibrosis [19].

We subsequently assessed circNlgn levels in human colitis samples through immunohistochemistry. Acute ulcerative colitis (A-UC) samples revealed significantly higher levels of Nlgn173 compared to remission stage (R-UC) and normal colon samples (Fig. 1B). In situ hybridization immunofluorescence staining showed that both circNlgn and Nlgn173 levels were markedly elevated in A-UC compared to remission ulcerative colitis (R-UC) and normal colon (Fig. 1C, full panel provided in Fig. S1). Reverse transcription-polymerase chain reaction (RT-PCR) analysis showed that the increased expression was specific to circNlgn but not the Nlgn mRNA (Fig. 1D). In a pairwise analysis, the inflamed areas of A-UC displayed higher levels of circNlgn compared to the unaffected A-UC regions (Fig. 1E).

To investigate the impact of circNlgn on colitis development, we utilized a mouse colitis model by administering dextran sulfate sodium (DSS) to C57BL/6J mice. Colon tissue staining showed that the histological damage score was significantly increased in the DSS-treated mice, while Ki67 levels increased at lower concentration but declined at higher concentration of DSS (Fig. S2A). This was due to a large number of cell apoptosis at the higher concentration of DSS (Fig. S2B). In situ hybridization indicated a substantial up-regulation of both circNlgn and the translated protein Nlgn173 in response to DSS treatment (Fig. 1F and Fig. S2C). Additionally, we subjected the mice to 2,4,6-trinitrobenzenesulfonic acid (TNBS) treatment, where we observed elevated levels of circNlgn through RT-PCR and Nlgn173 via Western blotting in the colonic mucosa of chemically treated mice relative to the control group (Fig. 1G).

Impact of circNlgn in transgenic mice on colitis susceptibility

Given the pivotal role of circNlgn in regulating cellular activities, we developed transgenic mouse lines expressing circNlgn [19] to investigate its influence on colitis development. We conducted experiments involving treatment with DSS and TNBS, which revealed several noteworthy observations. In comparison to wild-type (WT) mice, circNlgn-transgenic mice exhibited reduced body weight, higher bleeding scores, elevated stool scores, and increased disease activity index (DAI) (Fig. 2A). Furthermore, in mice administered with fluorescein isothiocyanate (FITC)-dextran, the circNlgn-transgenic mice displayed higher levels of FITC-dextran in their serum compared to controls (Fig. 2B). Consequently, the circNlgn-transgenic mice demonstrated decreased survival rates relative to the controls (Fig. 2C). Upon dissection, it became evident that the transgenic mice had shorter colon lengths compared to the controls (Fig. 2D).

Colon tissues were subjected to RT-PCR (Fig. S3A) and in situ hybridization (Fig. S3, B and C) to confirm increased expression of circNlgn and Nlgn173. Hematoxylin and eosin (H&E) staining showed that the circNlgn-transgenic mice displayed higher histological damage score than WT mice (Fig. S4A). Decreased Ki67 staining was observed in the colitis tissues (Fig. 2E and Fig. S4B) that also showed increased cell apoptosis (Fig. S4C and D). The colonic mucosa was collected to measure cytokine expression. It showed increased levels of cytokines in the colitis tissues compared with the controls (Fig. 2F and Fig. S4E).

To confirm the effect of circNlgn, we delivered 2 small interfering RNAs (siRNAs) targeting the junction sequence of circNlgn and a mixmer targeting translation of circNlgn to the DSS-treated mice. We detected increased body weight, lower bleeding score, lower stool score (Fig. S5A), and lower DAI (Fig. 3A) relative to the oligo control. In mice fed with FITC-dextran, the siRNA- and mixmer-treated mice showed lower amounts of FITC-dextran in the serum than the controls (Fig. 3B). As a consequence, these mice showed increased survival rates relative to the controls (Fig. 3C). Longer colons were detected in the siRNA- and mixmer-treated mice than the control (Fig. 3D and Fig. S5B). H&E staining of mouse colon sections displayed lower histological damage score in the siRNA- and mixmer-treated mice than in the control mice (Fig. 3E and Fig. S6A). Ki67 staining detected increased number of positive cells (Fig. 3F and Fig. S6B), consistent with decreased TUNEL-positive cells (Fig. 3G and Fig. S6C), in the siRNA- and mixmer-treated mice compared with the controls. RT-PCR confirmed the silencing of circNlgn by both siRNAs

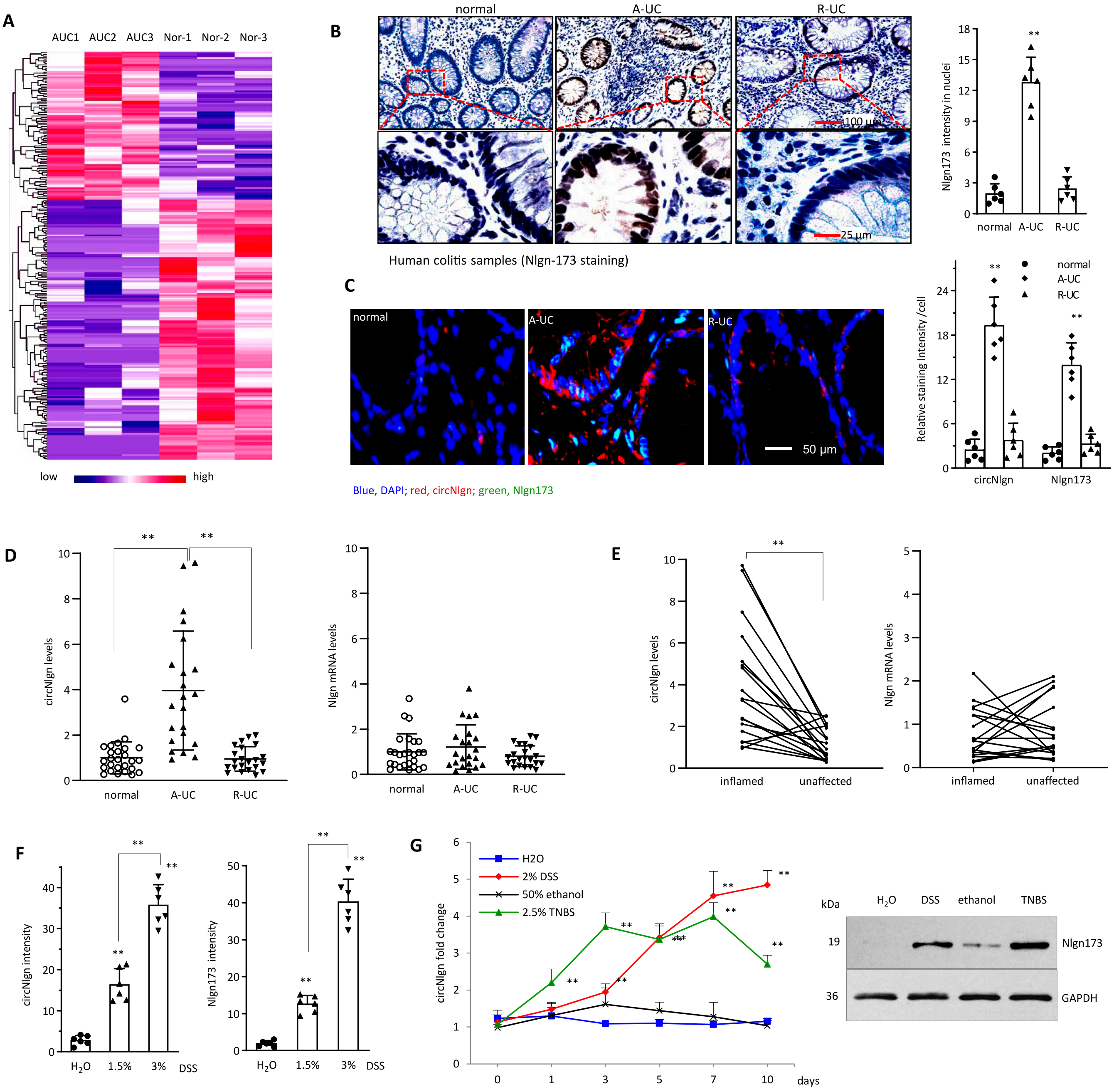

Fig. 1. Up-regulation of circNlgn in A-UC. (A) Heatmap illustrating differentially expressed circRNA profiles in human colon mucus with or without A-UC. (B) Immunohistochemical staining showing Nlgn173 protein levels in human colon mucus with A-UC and R-UC. ImageJ analysis showed human colon tissues with A-UC expressed high levels of Nlgn173 protein. $^{**}P < 0.01$ versus normal ($n = 6$). (C) In situ hybridization immunofluorescence staining showing circNlgn and Nlgn173 protein levels in human colonic mucosa with A-UC and R-UC. ImageJ analysis demonstrated elevated levels of circNlgn and Nlgn173 protein in human colon tissues with A-UC. $^{**}P < 0.01$ versus normal ($n = 6$). (D) Human colonic mucosa collected from inflamed and unaffected areas of A-UC cases and subjected to RT-PCR, showing that inflamed mucosa expressed high levels of circNlgn. $^{**}P < 0.01$ versus unaffected ($n = 18$). (E) Human colonic mucosa with A-UC or R-UC was lysed and subjected to RT-PCR, showing that A-UC mucosa expressed high levels of circNlgn. $^{**}P < 0.01$ versus normal (normal, $n = 26$; A-UC and R-UC, $n = 22$). (F) Left: C57BL/6J mice were orally administered with 1.5% or 3% DSS for 7 d followed by a return to tap water for 3 d. Colonic mucosa was collected and subjected to in situ hybridization staining. ImageJ analysis showed that DSS-treated mice expressed higher levels of circNlgn than the control group, with a dose-dependent effect. $^{**}P < 0.01$ versus H_2O group ($n = 6$). Right: ImageJ analysis of the colonic mucosa slides showed that the DSS-treated mouse colonic mucosa expressed higher levels of Nlgn173 than the control group, which was dose related. $^{**}P < 0.01$ versus H_2O group ($n = 6$). (G) Left: In the 2% DSS and 2.5% TNBS-induced mouse colitis models, mouse colonic mucosa was collected at the indicated time points. RT-PCR showed that the colonic mucosa of DSS or TNBS-treated mice expressed high levels of circNlgn. $^{**}P < 0.01$ versus H_2O or 50% ethanol ($n = 5$). Right: The above mouse colonic mucosa was lysed and subjected to Western blot with antibody against Nlgn173, showing that DSS- or TNBS-treated mouse colonic mucosa expressed high levels of Nlgn173.

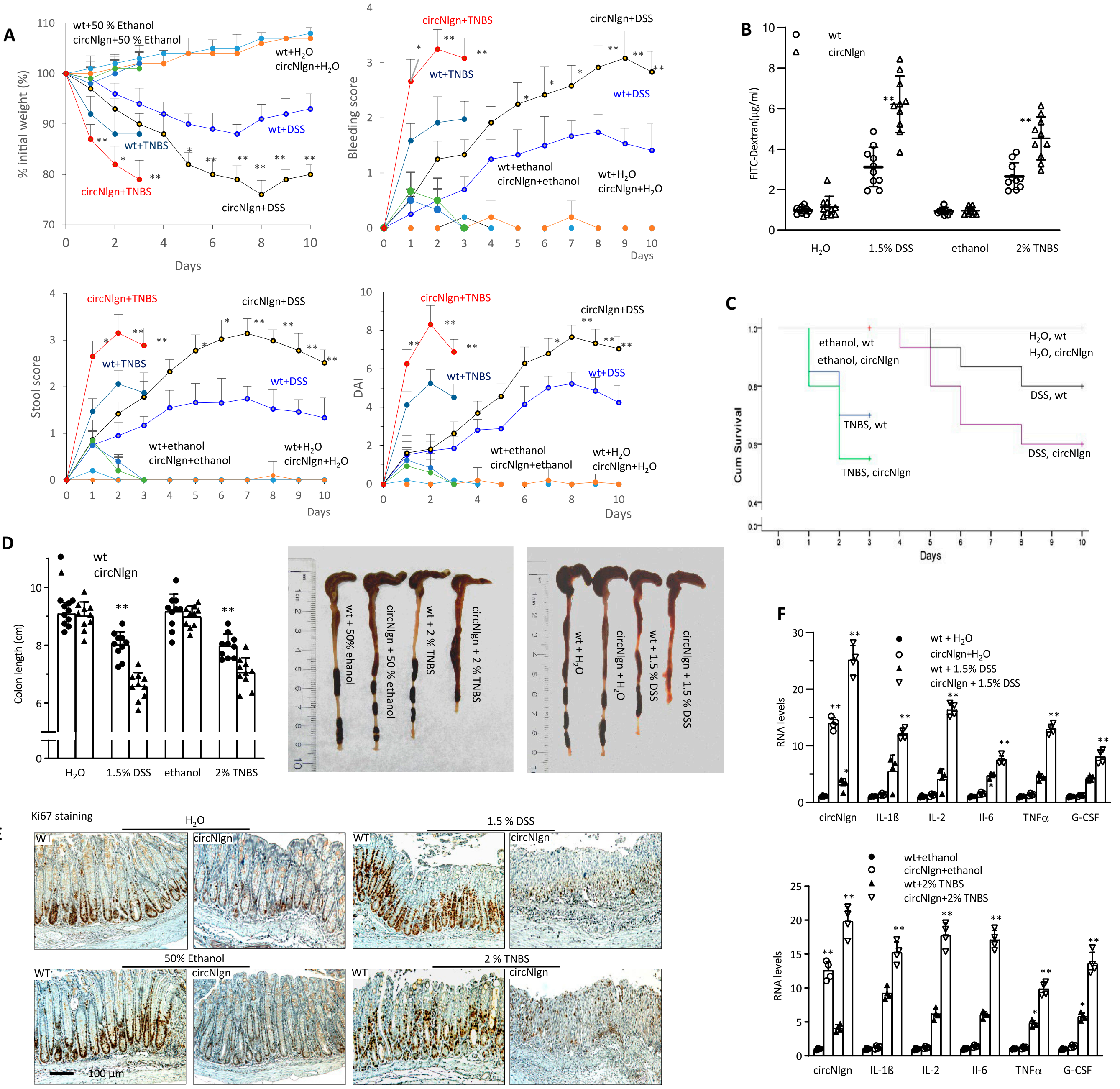

Fig. 2. Susceptibility of colitis in the circNlgn-transgenic mice. (A) Experimental mice were administered 1.5% DSS or 2.0% TNBS, and body weight was assessed daily until euthanized. circNlgn(+) mice exhibited decreased body weight compared to WT mice after DSS or TNBS treatment. circNlgn(+) mice showed higher bleeding score than WT mice after DSS or TNBS treatment. circNlgn(+) mice showed higher stool score than WT mice after DSS or TNBS treatment. circNlgn(+) mice showed higher DAI than WT mice after DSS or TNBS treatment. ***P* < 0.05, ***P* < 0.01 versus WT (*n* = 10). (B) Mice were fed with FITC-dextran (600 mg/kg) 4 h before euthanized, and serum levels of FITC-dextran were assessed by enzyme-linked immunosorbent assay (ELISA). circNlgn(+) mice exhibited high levels of FITC-dextran in the serum after DSS or TNBS treatment. ***P* < 0.01 versus WT (*n* = 10). (C) Kaplan–Meier survival test showed that circNlgn(+) transgenic mice exhibited decreased survival in the DSS- or TNBS-induced mouse colitis model. ***P* < 0.05, ***P* < 0.01 versus WT (control, *n* = 5; chemical treated, *n* = 10). (D) Left: The graph showed that circNlgn(+) mice had shorter colon length compared to the WT mice after DSS or TNBS treatment. ***P* < 0.01 versus WT (*n* = 10). Right: Typical images showing the length of colon in above WT and circNlgn(+) mice. (E) Typical images of immunofluorescence staining showing Ki67 expression in mouse colonic mucosa of the above mice. (F) Colonic mucosa was collected and subjected to RT-PCR, showing that circNlgn(+) mouse mucosa expressed much higher levels of interleukin-1β (IL-1β), IL-2, IL-6, TNFα, and granulocyte colony-stimulating factor (G-CSF) mRNA after DSS (upper) or TNBS treatment (lower). ***P* < 0.05, ***P* < 0.01 versus WT (*n* = 4).

used (Fig. S7A). In situ hybridization showed that the mixmer significantly inhibited translation of Nlgn173, while it had no effect on circNlgn levels (Fig. 3H and Fig. S7B and C). Cytokine expression was inhibited by the siRNAs and mixmer (Fig. 3I and Fig. S7D).

Expression of circNlgn suppressed mCEC nuclear actin polymerization in colitis models

To explore the role of Nlgn173 in promoting colitis development, we conducted an immunoprecipitation assay using primary mouse colonic epithelial cells (mCECs) isolated from

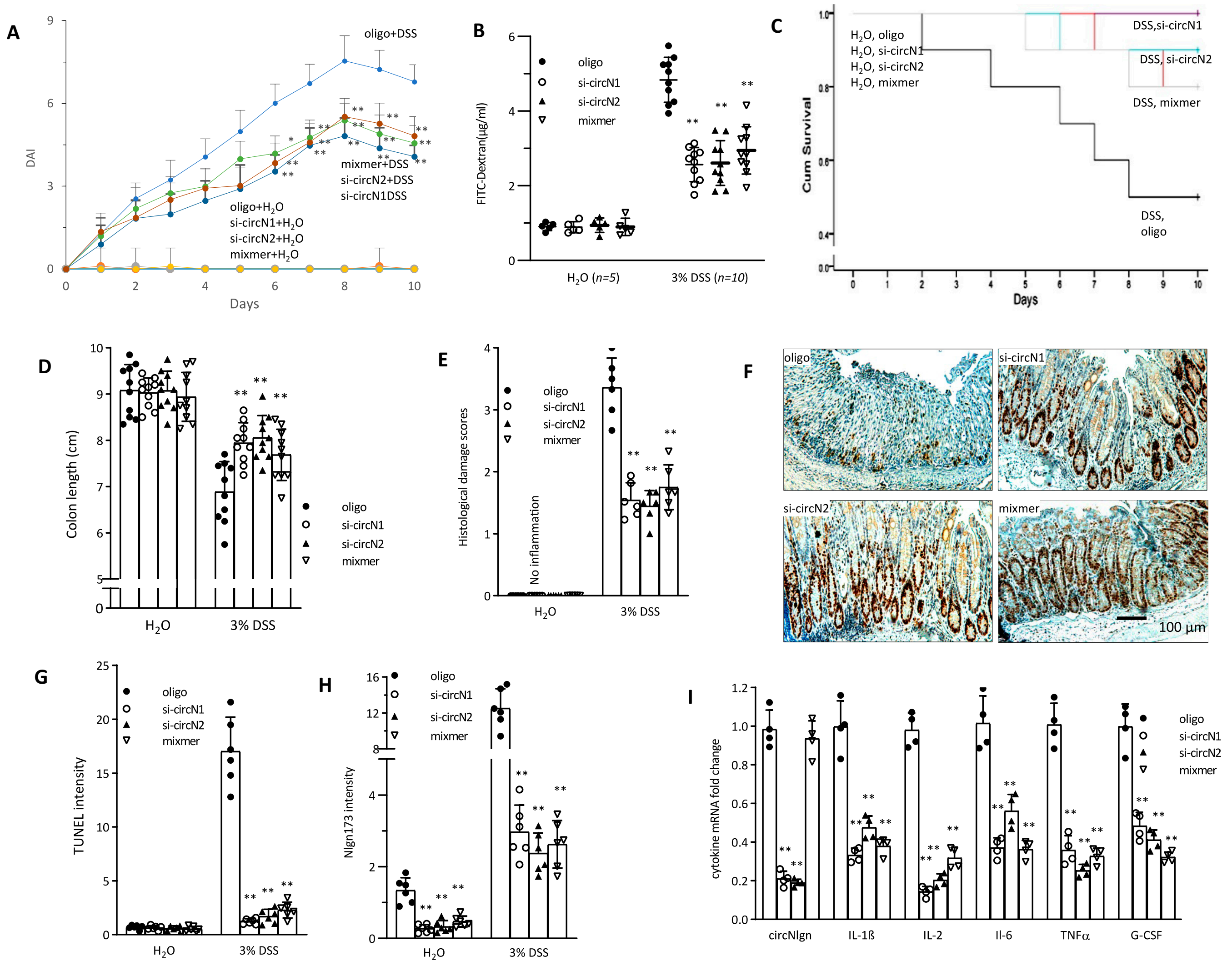

Fig. 3. Improvement of colitis outcome by targeting Nlgn173. (A) A graph showing that silencing circNlgn with siRNAs or blocking circNlgn translation with a mixmer mitigated DSS-induced DAI increase. $**P < 0.05$, $**P < 0.01$ versus oligo ($n = 10$). (B) A graph showing that circNlgn siRNAs- or mixmer-delivered mice had lower FITC-dextran levels in the serum after DSS treatment. $**P < 0.01$ versus oligo ($n = 10$). (C) Mice were delivered with circNlgn siRNAs or mixmer by nanoparticles as Materials and Methods described, and administrated with 3% DSS or H_2O. Kaplan–Meier survival test showed that silencing circNlgn with siRNAs or blocking circNlgn translation with a mixmer enhanced mouse survival in the 3% DSS-induced mouse colitis model. $**P < 0.05$, $**P < 0.01$ versus oligo (H_2O, $n = 5$; 3% DSS, $n = 20$). (D) Left: A graph showing that silencing circNlgn with siRNAs or blocking circNlgn translation with a mixmer prevented colon shortening induced by DSS treatment. $**P < 0.01$ versus oligo ($n = 10$). (E) Left: A graph showing that circNlgn siRNAs- or mixmer-delivered mouse colon sections displayed lower histological damage score than control mice after DSS treatment. $**P < 0.01$ versus oligo ($n = 6$). (F) Immunofluorescence staining showed that circNlgn siRNAs- or mixmer-delivered mouse mucosa expressed higher Ki67 levels than control mice after DSS treatment. (G) Left: ImageJ analysis of TUNEL staining showed that circNlgn siRNAs- or mixmer-delivered mouse mucosa displayed lower TUNEL intensity than those of control mice after DSS treatment. $**P < 0.01$ versus oligo ($n = 6$). (H) ImageJ analysis of in situ hybridization staining showed that circNlgn siRNAs-delivered colonic mucosa expressed lower levels of Nlgn173 in the nucleic. $**P < 0.01$ versus oligo ($n = 6$). (I) Left: Colonic mucosa was collected and subjected to RT-PCR, showing that circNlgn siRNAs- or mixmer-delivered mouse mucosa expressed much lower levels of IL-1β, IL-2, IL-6, TNFα, and G-CSF mRNA after DSS treatment. $**P < 0.05$, $**P < 0.01$ versus oligo ($n = 4$).

circNlgn-transgenic and WT mice. We utilized an antibody against Nlgn173, followed by mass spectrometry analysis, to identify Nlgn173-binding proteins. The results yielded a list of Nlgn173-binding partners (Fig. 4A). Notably, among several proteins essential for the nuclear translocation of Nlgn173, actin emerged as the most significant binding partner. It was noted that the read count of actin was much greater than Nlgn173, even though Nlgn173 was the direct target of the antibody and actin was indirectly pulled down by Nlgn173. The result suggested that one antibody molecule might bind one Nlgn173 and pulled down many actin molecules in the complex: F-actin rather than globular actin (G-actin) might be involved. The nuclear extract was then subjected to actin antibody precipitation. In this way, actin became the direct target of the antibody, and large number of read counts were expected for actin (Fig. 4B).

In light of the increased expression of Nlgn173 in colitis, we prepared lysates from the colonic mucosa of the mice with and without colitis induced by DSS and TNBS. We conducted an actin polymerization assay, which revealed that lysates from colitis-affected mucosa exhibited reduced actin polymerization in comparison to controls (Fig. 4C). Computational algorithm predicted the interaction of Nlgn173 and actin (Fig. 4D). The lysates were also subjected to subcellular fractionation. The nuclear extracts from the colitis showed decreased actin polymerization (Fig. 4E).

A

Identified Proteins	WT	circNlgn
Actin, cytoplasmic 2	37	140
Actin, cytoplasmic 1	0	137
Nlgn173	0	33
Exportin-1	0	18
Serine/arginine-rich splicing factor 1	7	13
Importin subunit beta-1	0	8
Exportin-2	2	10
Ubiquitin-60S ribosomal protein L40	5	8
Keratin, type II cytoskeletal 1	4	6
39S ribosomal protein L34, mitochondrial	5	7
Prohibitin-2	3	7
Lamin-B1	0	7
Importin-7	0	9
Prohibitin	3	5
Importin-9	0	6
F-actin-capping protein subunit beta	0	5
Importin subunit alpha-1	0	5

B

Identified Proteins	wt	circNlgn
Actin, cytoplasmic 2	606	580
Actin, cytoplasmic 1	608	572
Actin, alpha cardiac muscle 1	319	261
Beta-actin-like protein 2	112	81
Nlgn173	0	23
F-actin-capping protein subunit alpha-2	19	14
F-actin-capping protein subunit alpha-1	20	11
Actin-binding LIM protein 1	6	7
Polymerase delta-interacting protein 3	3	4
F-actin-capping protein subunit beta	11	1
Filamin A-interacting protein 1-like	3	1
Actin-related protein 3	8	0
Actin-related protein 2	5	0

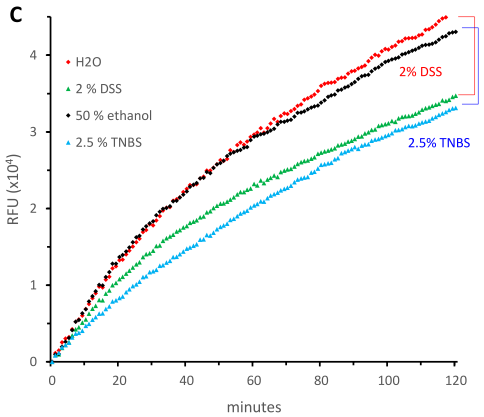

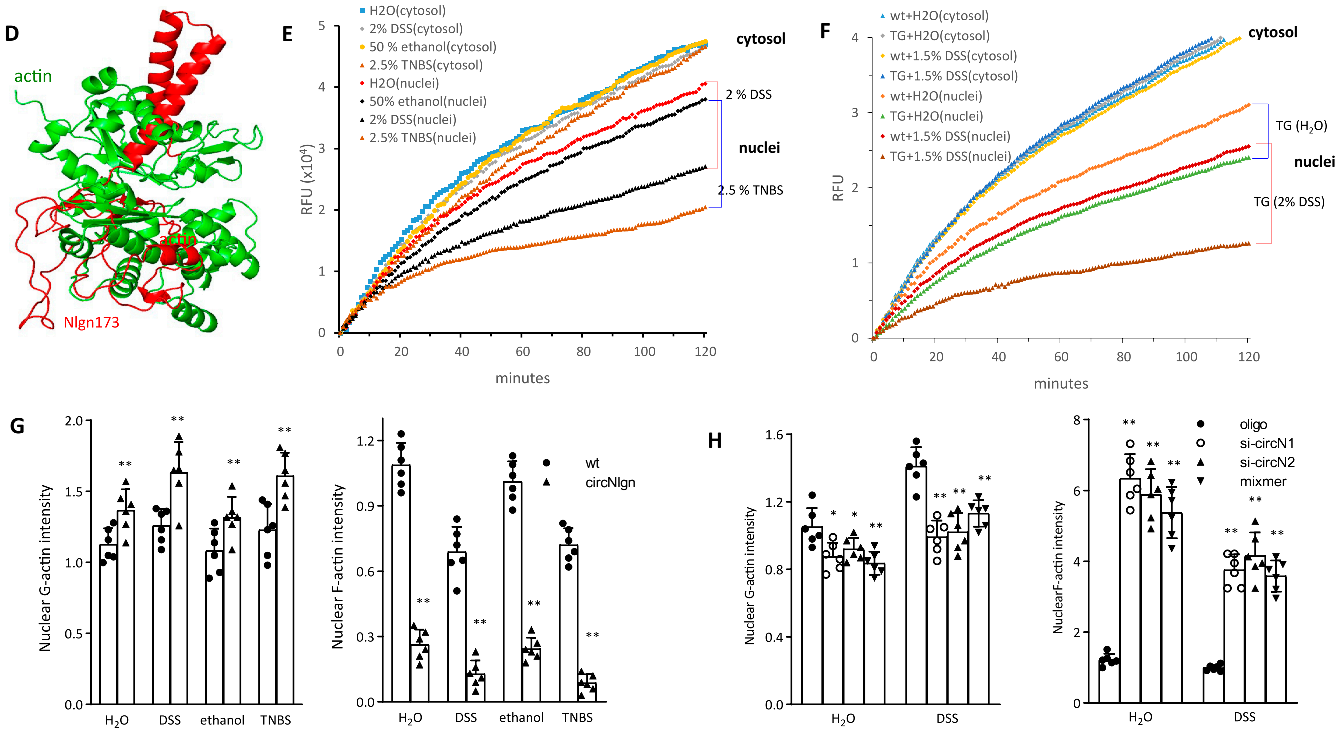

Fig. 4. Nlgn173 suppressed nuclear actin polymerization. (A) Primary mouse colonic epithelial cells (mCECs) were isolated from WT and circNlgn(+) transgenic mice and subjected to immunoprecipitation assay with antibody against Nlgn173. Mass spectrometry identified a list of Nlgn173-binding proteins. (B) Nuclear extracts of mCECs were subjected to immunoprecipitation with an antibody against actin. Mass spectrometry revealed a list of actin-binding proteins. In the presence of Nlgn173, actin did not precipitate Arp2/3. (C) In the 2% DSS- and 2.5% TNBS-induced mouse colitis models, mouse colonic mucosa was collected, lysed, and subjected to actin polymerization assay. The mucosa lysis of DSS- or TNBS-treated mice suppressed actin polymerization. (D) Diagram showing the interaction of Nlgn173 and actin. (E) The mucosa was subjected to subcellular fractionation and processed to actin polymerization assay. Total lysate and nuclear lysate from the DSS- and TNBS-treated mice suppressed actin polymerization. (F) Colonic mucosa from WT and circNlgn(+) mice treated with or without 1.5% DSS was collected and processed to actin polymerization assay. Lysate of nuclear extract from circNlgn(+) mucosa showed suppression of actin polymerization. (G) Left: ImageJ analysis showed that circNlgn(+) mCEC nuclei expressed higher levels of G-actin relative to the WT. Right: ImageJ analysis showed that circNlgn(+) mCEC nuclei expressed lower levels of F-actin relative to WT. $^{**}P < 0.01$ versus WT ($n = 6$). (H) Left: Delivery of circNlgn siRNAs and mixmer in mCECs decreased G-actin in the nuclei. Right: Delivery of circNlgn siRNAs and mixmer increased F-actin in the nuclei. $^{**}P < 0.01$ versus oligo ($n = 6$).

Nuclear extract of colonic mucosa was also prepared from the circNlgn-transgenic and WT mice with colitis induced by DSS and TNBS. Transgenic expression of Nlgn173 decreased actin polymerization (Fig. 4F). Primary mCECs were isolated from the circNlgn-transgenic and WT mice with colitis induced by DSS. Increased expression of Nlgn173 decreased actin polymerization (Fig. S8A), while silencing circNlgn increased actin polymerization (Fig. S8B). Ectopic expression of circNlgn in cell lines also decreased actin polymerization (Fig. S8C).

Above evidence indicated expression of circNlgn suppressed nuclear actin polymerization. Our previous study demonstrated that circNlgn encoded a protein isoform Nlgn173, which was mainly translocated to nuclei by binding to Lamin B1. Thus, silencing Lamin B1 prevented Nlgn173 nuclear translocation, and accumulation of Nlgn173 was found in cytosol [19]. To observe the effect of the nuclear protein Nlgn173 on actin polymerization, the circNlgn-positive mCECs were transfected with siRNAs targeting Lamin B1, followed by subcellular actin fractionation. Actin polymerization assays showed that the nuclear

extract from the cells transfected with Lamin B1 siRNAs promoted actin polymerization, but the cytosolic extract repressed actin polymerization (Fig. S8D), confirming the inhibitory effect of Nlgn173 in actin polymerization.

We then examined the effects of Nlgn173 on colitis development in the mouse colitis model. Colon tissues treated with DSS or TNBS were sectioned and subjected to G-actin and F-actin immunostaining. ImageJ analysis showed that the colon tissues from the circNlgn-transgenic mice expressed higher levels of G-actin but lower levels of F-actin in the nuclei relative to WT (Fig. 4G). The total levels of G-actin and F-actin were not affected in both the DSS and TNBS colitis models (Fig. S9). Silencing circNlgn by circNlgn siRNAs and blocking its translation by mixmer prevented nuclear actin polymerization, resulting in decreased G-actin but increased F-actin in the nuclei compared to the oligo controls in the DSS model (Fig. 4H and Fig. S10A), while the total levels of G-actin and F-actin were similar (Fig. S10B). Human colon samples were also subjected to G-actin and F-actin immunostaining. Human A-UC samples revealed higher levels of nuclear G-actin and lower levels of F-actin compared to normal colon samples (Fig. S11).

Nlgn173 inhibits Arp2/3 complex binding to actin and represses actin polymerization

While we detected pulldown of Nlgn173 in the circNlgn-transgenic mice, we did not detect actin-related protein 2 and 3 (ARP2/3), the 2 molecules essential for driving actin polymerization [21–23]. To validate the role of Nlgn173 in actin polymerization, we purified Nlgn173 from the circNlgn-transfected 293T cells (Fig. 5A, left). The purified Nlgn173 inhibited actin polymerization (Fig. 5A, right). The inhibition of actin polymerization by Nlgn173 was dose dependent and required inclusion of Arp2/3 (Fig. S12A and B). Solid-phase microplate protein binding assay was used to confirm the interaction between F-actin and Arp2/3. Addition of the purified Nlgn173 repressed Arp2/3 binding to F-actin (Fig. 5B).

We further validated the interaction among Arp2/3, F-actin, and Nlgn173 through a direct binding assay, using an actin-binding protein biochemistry kit (Cat# BK001). Western blot analysis following the separation of F-actin and G-actin binding partners confirmed that the presence of Nlgn173 inhibited the interaction between Arp2/3 and F-actin (Fig. 5C). Immunoprecipitation with antibody against actin showed that both Arp2/3 and Nlgn173 bound F-actin. The binding of Arp2/3 to F-actin decreased in the presence of Nlgn173.

To test the interaction in cells, we isolated mCECs from circNlgn-transgenic mice followed by subcellular fractionation and Western blot. Nlgn173 was primarily detected in the nuclei, where the levels of actin and Arp2/3 were lower relative to the levels in the cytosol (Fig. 5D). Direct interaction was performed using immunoprecipitation and Western blot. Precipitation of Nlgn173 coprecipitated actin in the nuclei. Actin immunoprecipitation pulled down Arp2/3 and Nlgn173 in the nuclei. Ectopic expression of Nlgn173 decreased Arp2/3 binding to actin. Arp2/3 precipitation pulled down actin in the nuclei. Expression of Nlgn173 decreased actin binding to Arp2/3.

We tested the role of endogenous Nlgn173 in mediating the interaction by delivering circNlgn siRNAs into mCECs and confirmed silencing and nuclear localization of Nlgn173 (Fig. S12C). Immunoprecipitation of Nlgn173 pulled down actin in the nuclei (Fig. S12D), and precipitation of actin pulled down Arp2/3 and Nlgn173, where silencing Nlgn173 increased Arp2/3 binding to actin (Fig. 5E). Arp2/3 precipitation pulled down actin in the nuclei, where silencing Nlgn173 increased actin binding to Arp2/3 (Fig. 5E). We also silenced Lamin B1 to inhibit Nlgn173 nuclear translocation (Fig. S12E). Immunoprecipitation of Nlgn173 coprecipitated actin in the nuclei, which was inhibited by silencing Lamin B1 (Fig. S12F). Actin precipitation pulled down Arp2/3 and Nlgn173. Nuclear Arp2/3 precipitation pulled down actin that was promoted by silencing Lamin B1. Silencing Lamin B1 increased Arp2/3 binding to actin in the nuclei but decreased its binding to actin in the cytosol.

We then examined the interaction in the colitis model. Primary mCECs were isolated from circNlgn-transgenic mice, in which colitis was induced with 1.5% DSS and 2% TNBS. Western blot confirmed increased expression and nuclear translocation of Nlgn173 (Fig. 6A). Immunoprecipitation of Nlgn173 pulled down actin in the nuclei; actin precipitation pulled down Arp2/3 and Nlgn173; Arp2/3 precipitation pulled down actin (Fig. 6B). Increased expression of Nlgn173 decreased actin binding to Arp2/3. Decreased expression and nuclear translocation were observed by treatment with circNlgn siRNAs and mixmer (Fig. 6C). Nlgn173 precipitation pulled down actin in the nuclei, while actin precipitation pulled down Nlgn173 and Arp2/3; Arp2/3 precipitation pulled down actin in the nuclei (Fig. 6D). Decreased expression of Nlgn173 enhanced the interaction between actin and Arp2/3.

Validation of the functions of circNlgn-encoded protein Nlgn173

We validated the effect of the translated protein Nlgn173 on colitis development using various expression constructs, including circNlgn, linNlgn (expressing a linear mRNA to translate Nlgn173), Precursor (expressing a linear RNA that could not translate Nlgn173), circ-mut (expressing a mutated circRNA that could not translate Nlgn173), circ-164 (expressing a mutated circRNA that could translate a smaller protein, Nlgn164), the full-length Nlgn, and the control vector, as previously described [19]. In all of these expression constructs, only circNlgn and linNlgn encoded the protein Nlgn173. Our experiments demonstrated that the expression of circNlgn or Nlgn173 in DSS-treated mice was critical for observing the altered phenotypes: increased DAI (Fig. 7A), increased levels of FITC-dextran in the serum (Fig. 7B), decreased body weight (Fig. S13A), higher bleeding score (Fig. S13B), higher stool score (Fig. S13C), and shortened colon length (Fig. S13D). These phenotypic changes correlated with decreased survival rates compared to the controls (Fig. 7C). Tissue staining revealed increased histological damage scores (Fig. 7D and Fig. S14A), decreased Ki67-positive cells, and elevated apoptosis in constructs expressing Nlgn173 (Fig. 7E and Fig. S14B-C). RT-PCR and in situ hybridization confirmed the up-regulation of circNlgn (Fig. S15A) and increased levels of Nlgn173 (Fig. S15B and C).

In an actin polymerization assay, we confirmed that mucosa lysates from the circNlgn and linNlgn groups exhibited reduced actin polymerization relative to controls, highlighting the essential inhibitory effect of Nlgn173 on actin polymerization (Fig. 7F). Additionally, cytokine expression was enhanced in constructs expressing Nlgn173 (Fig. 7G).

We further validated the interaction of Nlgn173 with F-actin and Arp2/3. After Western blot analysis confirming expression of proteins by circNlgn, linNlgn, circ-164, and full-length Nlgn constructs (Fig. S15D), Nlgn173 proteins expressed by circNlgn

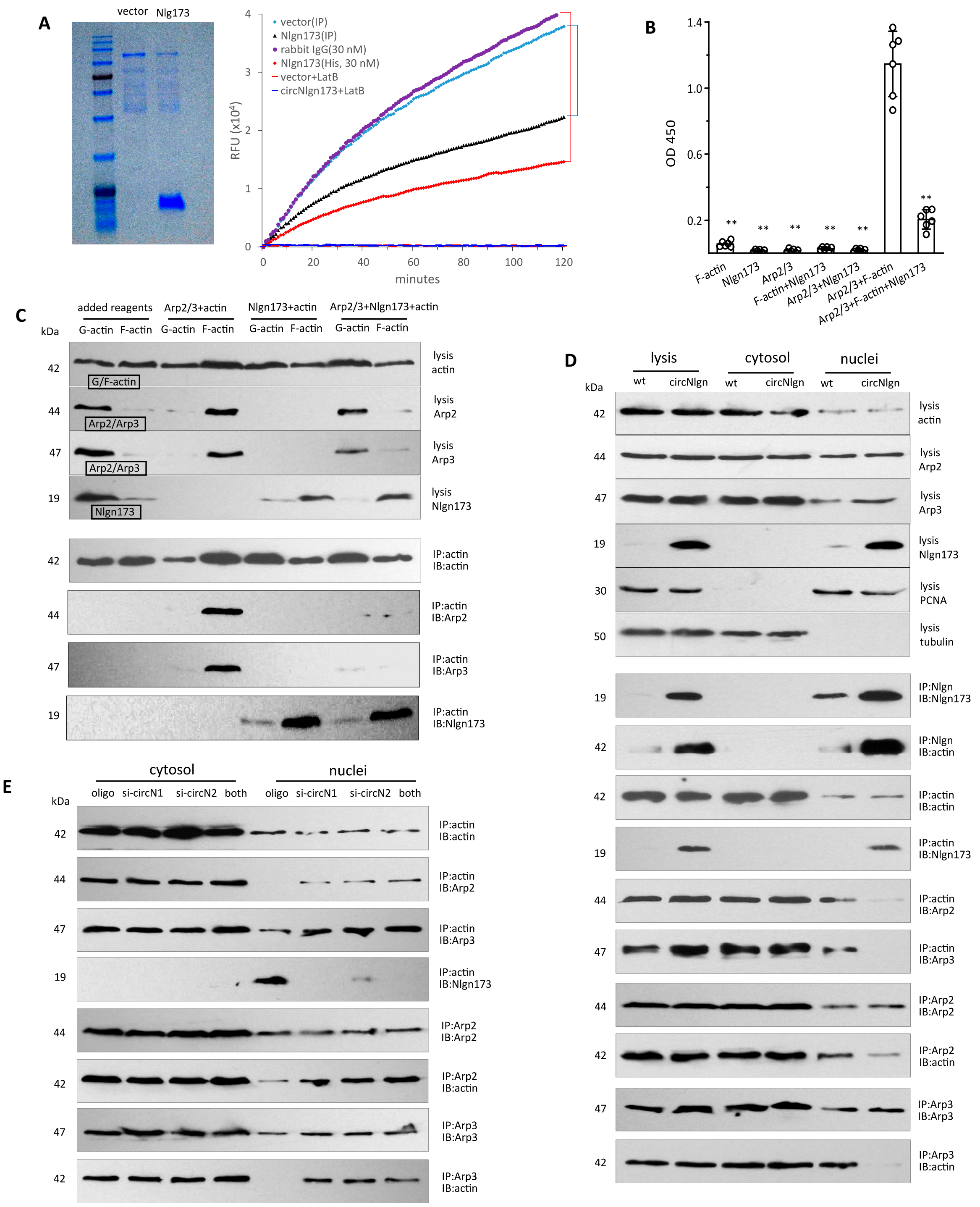

Fig. 5. Nlgn173 repressed nuclear actin polymerization was Arp2/3 dependent. (A) Left: Coomassie blue staining confirmed the purification of Nlgn173 from the circNlgn-transfected 293T cells. Right: The purified Nlgn173 suppressed actin polymerization. (B) Solid-phase microplate protein binding assay confirmed the interaction between F-actin and Arp2/3 with or without Nlgn173. The purified Nlgn173 repressed Arp2/3 binding to F-actin. ***P* < 0.01 versus Arp2/3+F-actin ($n = 6$). (C) F-actin (40 μg) and G-actin (40 μg) were mixed with 5 μg of Arp2/3 (from an actin-binding protein biochem kit, Cat# BK001) with or without 5 μg of purified Nlgn173. The mixture was subjected to fractionation to separate F-actin (pellet) and G-actin (supernatant), followed by Western blot. Incubation with Arp2/3 increased F-actin levels, and incubation with Nlgn173 increased G-actin levels. Incubation with Nlgn173 abolished the effect of Arp2/3 on increasing F-actin levels. Arp2/3 bound F-actin, which was blocked by Nlgn173. Nlgn173 bound F-actin, but the binding did not decrease after incubated with Arp2/3. Immunoprecipitation with an antibody against actin showed that both Arp2/3 and Nlgn173 bound F-actin. Binding of Nlgn173 to F-actin decreased the interaction of Arp2/3 with F-actin. (D) mCECs isolated from WT and circNlgn(+) mice were subjected to subcellular fractionation followed by Western blot. Nlgn173 was mainly detected in the nuclei, where the levels of actin and Arp2/3 were lower compared to cytosol. Immunoprecipitation with an antibody against Nlgn173 coprecipitated actin in the nuclei. Immunoprecipitation with an antibody against actin coprecipitated Arp2/3 and Nlgn173 in the nuclei. Expression of Nlgn173 decreased Arp2/3 binding to actin. Immunoprecipitation with an antibody against Arp2 or Arp3 coprecipitated actin in the nuclei. Expression of Nlgn173 decreased actin binding to Arp2/3. (E) Precipitation of actin pulled down Arp2/3 and Nlgn173 in the nuclei. Expression of Nlgn173 decreased Arp2/3 binding to actin in the nuclei. Immunoprecipitation with an antibody against Arp2 or Arp3 coprecipitated actin in the nuclei. Expression of Nlgn173 decreased actin binding to Arp2/3.

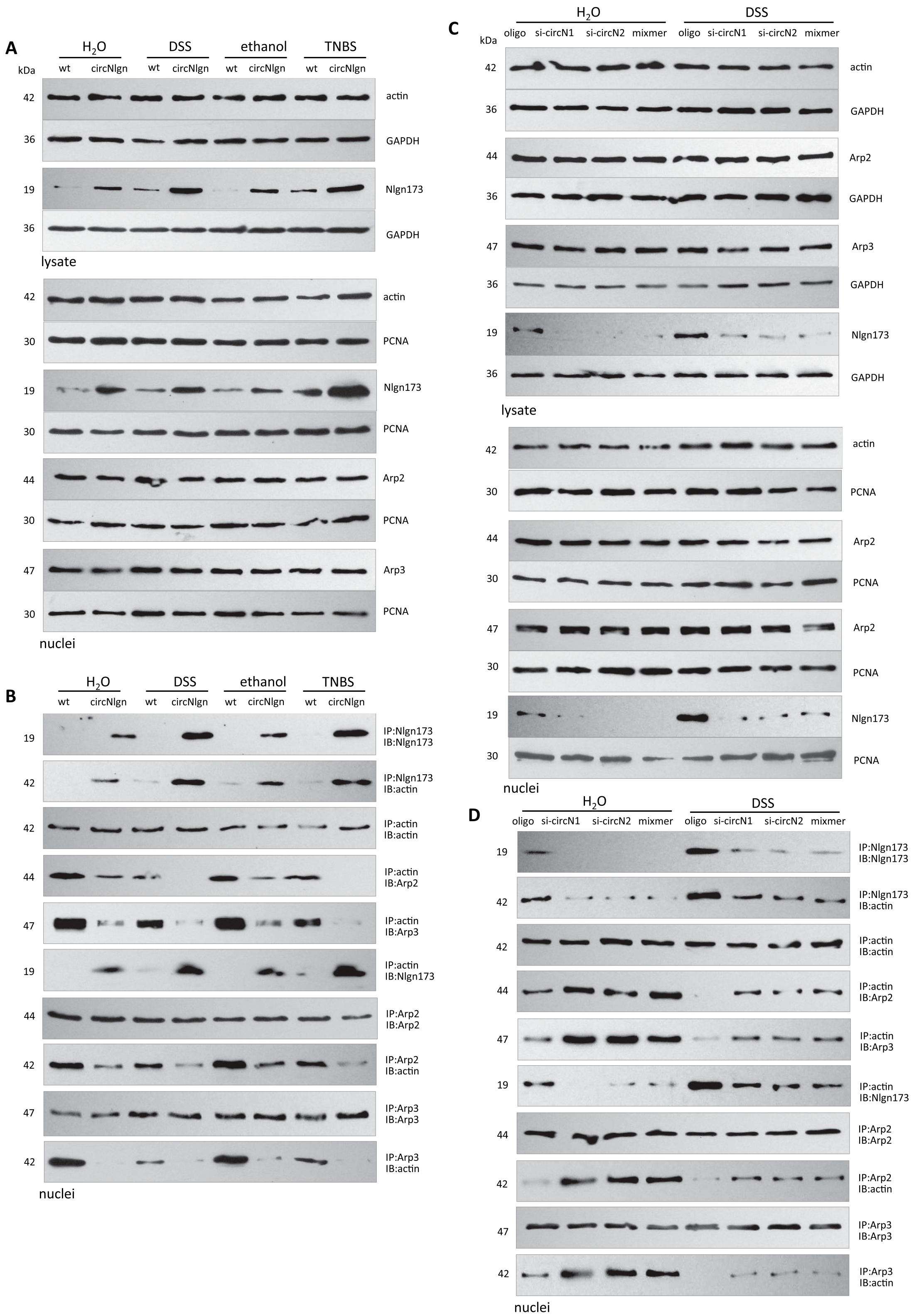

Fig. 6. Expression of circNlgn represses Arp2/3 binding to actin in mCEC nuclei. (A) Upper, mCECs were isolated from WT and circNlgn(+) mice treated with 1.5% DSS or 2% TNBS and subjected to Western blot. Treatment with DSS and TNBS increased Nlgn173 levels. Lower, Subcellular fractionation and Western blot showed that treatment with DSS and TNBS increased Nlgn173 levels in the nuclei. (B) Immunoprecipitation with an antibody against Nlgn173 coprecipitated actin in the nuclei. Actin precipitation coprecipitated Arp2/3 and Nlgn173. Arp2/3 precipitation pulled down actin. Increased expression of Nlgn173 decreased actin binding to Arp2/3. (C) mCECs transfected with circNlgn siRNAs and mixmer were subjected to Western blot. Delivery of circNlgn siRNAs and mixmer decreased Nlgn173 expression in mCECs. Subcellular fractionation followed by Western blot detected Nlgn173 in the mCEC nuclei. (D) Immunoprecipitation with antibody against Nlgn173 showed that decreased Nlgn173 expression precipitated lower levels of actin in the nuclei. Immunoprecipitation with an antibody against actin coprecipitated Arp2/3 and Nlgn173. Arp2/3 precipitation pulled down actin. Expression of Nlgn173 repressed Arp2/3 binding to actin.

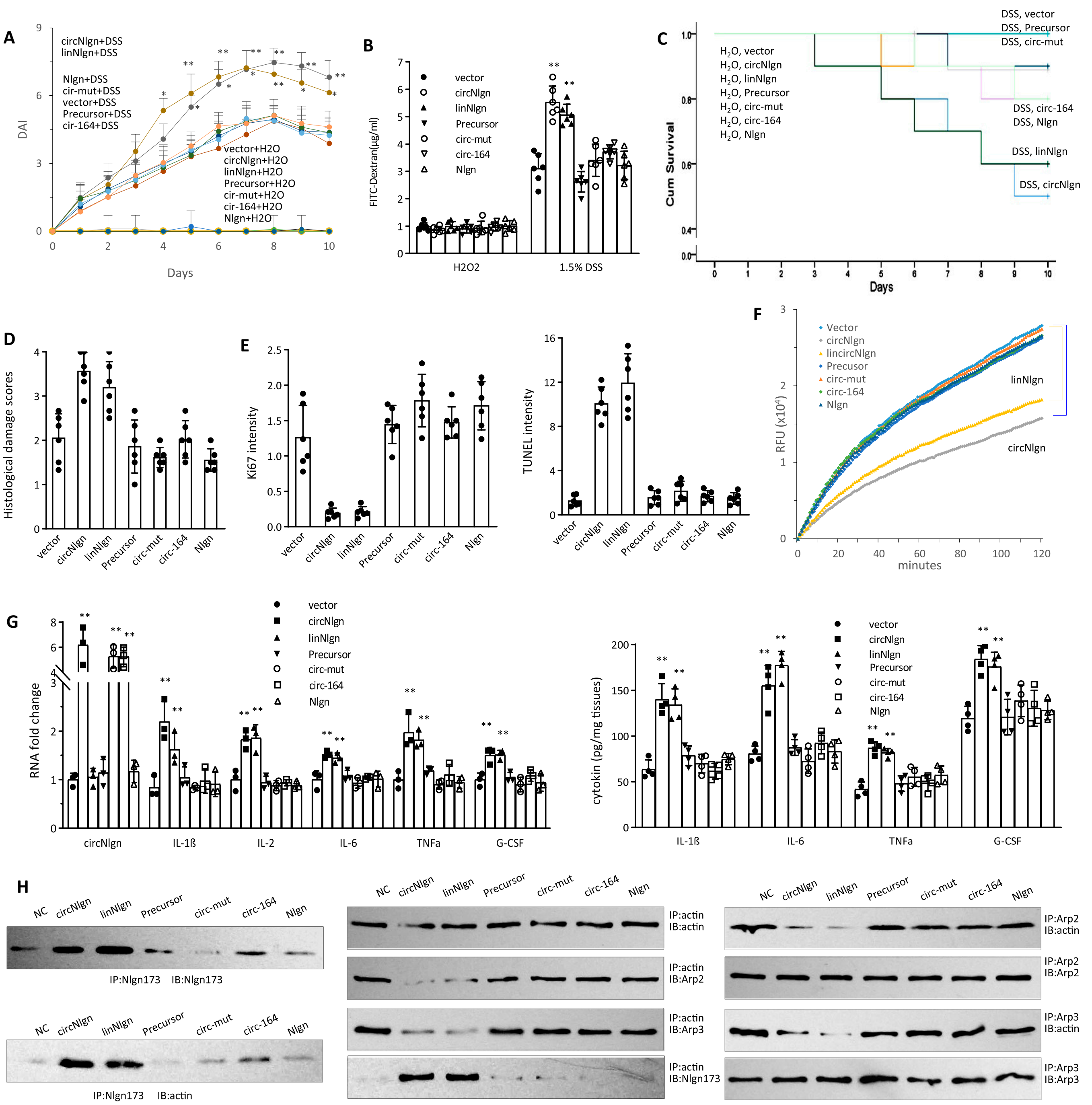

Fig. 7. The circNlgn translated protein is essential for colitis development. (A) A control vector, circNlgn, linNlgn, Precursor, circ-mut, circ-164, and Nlgn were delivered by nanoparticles as Materials and Methods described. The graph showed that delivery of circNlgn or linNlgn increased mouse colon DAI after DSS treatment. $^{**}P < 0.05$, $^{**}P < 0.01$ versus vector ($n = 10$). (B) ELISA analysis showed that mice delivered with circNlgn or linNlgn had higher FITC-dextran levels in the serum after DSS treatment. $^{**}P < 0.01$ versus vector ($n = 10$). (C) Kaplan–Meier survival test showed that delivery of circNlgn or linNlgn decreased mouse survival in the 1.5% DSS-induced mouse colitis model. $^{**}P < 0.05$, $^{**}P < 0.01$ versus vector (H_2O, $n = 5$; DSS-treated, $n = 20$). (D) The graph showed that circNlgn- or linNlgn-delivered mouse colon sections displayed higher histological damage score than control mice after DSS treatment. $^{**}P < 0.01$ versus vector ($n = 6$). (E) Left: ImageJ analysis of immunofluorescence staining showed that circNlgn- or linNlgn-delivered mouse mucosa expressed lower Ki67 levels than control mice after DSS treatment. Right: TUNEL staining showed that circNlgn or linNlgn delivery displayed higher TUNEL intensity than the controls. $^{**}P < 0.01$ versus vector ($n = 6$). (F) Colonic mucosa of control vector, circNlgn, linNlgn, Precursor-, circ-mut-, circ-164-, or Nlgn plasmid-delivered mice was collected and processed for an actin polymerization assay, showing that circNlgn- or linNlgn-delivered mucosa nuclear extracts could repress actin polymerization. (G) Left: Colonic mucosa of the DSS-treated mice was collected and subjected to RT-PCR, showing that circNlgn- or linNlgn-delivered mouse mucosa expressed higher levels of IL-1β, IL-2, IL-6, TNFα, and G-CSF mRNA after DSS treatment. Right: The supernatant were subjected to ELISA, showing that circNlgn- or linNlgn-delivered mouse mucosa secreted higher levels of IL-1β, IL-6, TNFα, and G-CSF after DSS treatment. $^{**}P < 0.05$, $^{**}P < 0.01$ versus vector ($n = 4$). (H) Left: Colonic mucosa from mouse delivered with different plasmids and treated with 1.5% DSS were subjected to nuclear fractionation and immunoprecipitation. The nuclear fraction was precipitated with an antibody against Nlgn173. Nlgn173 precipitation pulled down actin. Middle: The fraction was precipitated with an actin antibody. Actin precipitation pulled down Arp2/3 and Nlgn173. Right: The fraction was precipitated with an Arp2 or Arp3 antibody. Arp2 or Arp3 precipitation pulled down actin.

and linNlgn were detected in the nuclei (Fig. S15E). Immunoprecipitation with antibody against actin, Nlgn, and Arp2/3 followed by Western blotting showed that the expression of Nlgn173 decreased the binding of F-actin to Arp2/3 (Fig. 7H).

Expression of circNlgn enhances nuclear F-actin tyrosine-53 phosphorylation

It has been reported that tyrosine-53 phosphorylation is involved in actin polymerization [24]. We tested whether actin phosphorylation was affected in mCECs isolated from circNlgn-transgenic mice and found transgenic expression of circNlgn decreased nuclear F-actin levels but increased F-actin phosphorylation at tyrosine (Fig. 8A). With comparable quantities of F-actin, the circNlgn group displayed increased F-actin phosphorylation of tyrosine but not threonine and serine, resulting in decreased pulldown of Arp2/3 (Fig. 8B). Precipitation of F-actin by phalloidin pulled down more Nlgn173 and p-Tyr-53 but less Arp2/3 in mCECs expressing circNlgn (Fig. 8C). Silencing circNlgn increased F-actin levels in the nuclei (Fig. 8D). At equal amounts of F-actin, actin precipitation pulled down decreased levels of phospho-tyrosine, but more Arp2/3, in mCECs treated with circNlgn siRNAs, while phosphorylation of threonine and serine was not affected (Fig. 8E). F-actin precipitation by phalloidin pulled down less Nlgn173 and p-Tyr-53 but more Arp2/3 in mCECs transfected with circNlgn siRNAs (Fig. 8F).

In the colitis model, phalloidin precipitated decreased levels of F-actin in the nuclei of circNlgn-transgenic mice, which was promoted by colitis development (Fig. 8G). At equal amounts of F-actin, increased p-Tyr-53 levels but decreased Arp2/3 were observed in the circNlgn-transgenic mice, which was promoted by colitis development. Delivery of circNlgn siRNAs and mixmer increased F-actin in the nuclei, which was promoted by DSS treatment (Fig. 8H). At equal amounts of F-actin, precipitation of F-actin pulled down less Nlgn173 and p-Tyr-53 but more Arp2/3.

Nlgn173 decreased PIAS3 expression but activated STAT3 and NF-κB

To uncover the mechanism by which Nlgn173 inhibited actin polymerization further, we examined expression of genes associated with colitis development. Mouse colonic mucosa obtained from circNlgn-transgenic and litter-matched negative mice was lysed and subjected to RT-PCR to analyze levels of 17 currently reported colitis-related genes. We detected several genes that were differentially expressed, in which PIAS3 was the most differentially expressed (Fig. 9A, left). Human colonic mucosa from A-UC and R-UC was then lysed and subjected to RT-PCR. Both A-UC and R-UC mucosa expressed lower levels of PIAS3 than the normal tissues (Fig. 9A, right).

To observe whether expression of PIAS3 was related with nuclear actin polymerization, we cultured mCECs with jasplakinolide (Jasp, enhancing actin polymerization), latrunculin B (LatB, decreasing actin polymerization) and cytochalasin D (CytD, decreasing actin polymerization), followed by RT-PCR. The cells showed increased PIAS3 levels when treated with Jasp but decreased PIAS3 levels when treated with LatB and CytD (Fig. 9B and Fig. S16A). It seemed that expression of PIAS3 was related with actin polymerization.

We previously generated a cell model to study nuclear F-actin function with minimal effect on the actin dynamics in cytosol, by using actin polymerization stabilizers and Exportin 6 (XPO6)/Importin 9 (IPO9) siRNAs [25]. The mCECs were transfected with XPO6 or IPO9 siRNAs and treated with Jasp to regulate nuclear F-actin expression levels, followed by RT-PCR. Silencing XPO6, which could enhance nuclear F-actin levels, up-regulated PIAS3 expression, while silencing IPO9, which repressed nuclear F-actin, down-regulated PIAS3 expression (Fig. 9C, left). The human fetal colon epithelial cell line (FHC) was transfected with XPO6 and IPO9 siRNAs and cultured in Jasp or cotransfected with mDia2, followed by subcellular and actin fractionation. Western blot showed that silencing XPO6 increased nuclear F-actin compared to control oligo after cultured in Jasp or cotransfected with mDia2; silencing IPO9 decreased nuclear F-actin compared to control oligo; both did not significantly change actin dynamics in total cell lysate (Fig. S16B). RT-PCR showed that silencing XPO6 up-regulated, while silencing IPO9 down-regulated expression of PIAS3 (Fig. 9C, right). We further confirmed the effect of nuclear actin polymerization on PIAS3 expression by introducing a number of actin-modified constructs into the FHC cells including YFP-NLS-β-actin (NLS-β-actin), YFP-NLS-β-actin G13R (G13R), YFP-NLS-β-actin S14C (S14C), and NLS-β-actin R62D (R62D) mCherry, in which NLS-β-actin and S14C can increase nuclear actin polymerization [25]. Expression of NLS-β-actin and S14C increased PIAS3 levels (Fig. 9D).

Thus, nuclear actin polymerization was confirmed to play roles in moderating colitis-related gene PIAS3 expression. Subsequently, we investigated the downstream signaling molecules of PIAS3, including signal transducer and activator of transcription 3 (STAT3) and NF-κB [26], using the circNlgn-transgenic mouse model. The mCECs isolated from cicNlgn transgenic mice displayed decreased PIAS3 expression at mRNA levels (Fig. 9E, left) and increased phosphorylation of STAT3 and NF-κB in cell nuclei (Fig. 9E, right).

Decreased expression of PIAS3 and activation of STAT3 and NF-κB were confirmed in circNlgn-transgenic mice treated with DSS and TNBS (Fig. 9F and Fig. S16C). Silencing circNlgn and inhibiting circNlgn translation increased PIAS3 expression but decreased activation of STAT3 and NF-κB (Fig. S17). In human colonic mucosa, decreased expression of PIAS3 and increased activation of STAT3 and NF-κB were also detected in A-UC (Fig. 9G and H). Taken together, circNlgn/Nlgn173 promoted A-UC via suppressing nuclear actin polymerization (Fig. 9I).

Discussion

Nuclear actin was first observed in the late 1970s, but its function remained poorly understood until recently [13,27]. It is now known that nuclear actin plays essential roles in regulating gene expression, DNA repair, and nuclear structural organization. One of the most well-studied functions of nuclear actin is its regulation of gene expression. Nuclear actin is known to associate with chromatin and play a role in modulating the accessibility of the transcription factors and other regulatory factors to DNA [28–31]. This results in changing gene expression, positively or negatively. Our results support this role, demonstrating that the expression of circNlgn leads to altered levels of nuclear F-actin, resulting in the up- and down-regulation of inflammatory factors associated with colitis development.

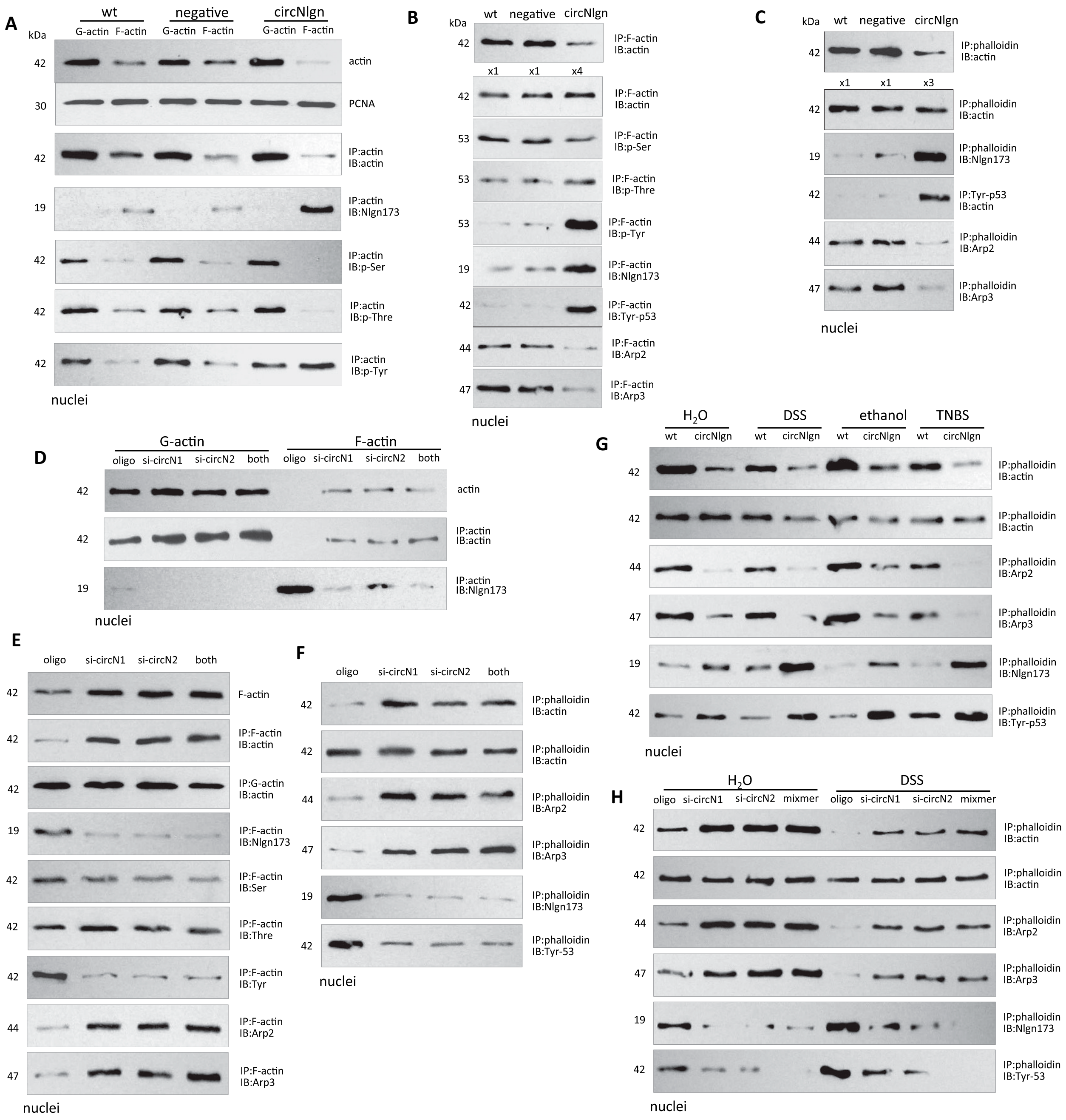

Fig. 8. Expression of circNlgn promotes tyrosine-53 phosphorylation of nuclear F-actin. (A) mCECs were isolated from WT, circNlgn(-) and circNlgn(+) mice. The nuclei were subjected to F/G-actin fractionation. Expression of circNlgn(+) decreased F-actin levels in the nuclei. Immunoprecipitation with antibody against actin followed by Western blot probed with antibodies against phospho-threonine, phospho-serine, and phospho-tyrosine showed that circNlgn expression promoted F-actin phosphorylation at tyrosine. (B) The nuclear F-actin fraction was subjected to actin immunoprecipitation. Precipitation of F-actin pulled down Nlgn173. Loading amount of precipitated products was modulated until similar expression levels of nuclear F-actin were detected for each sample. Phosphorylation of threonine and serine levels did not change, while phospho-tyrosine levels increased in F-actin. The levels of p-Tyrosine-actin increased in the precipitated F-actin, while the pulled down Arp2/3 amount decreased. (C) F-actin was precipitated with 20 μl of biotin–phalloidin (50 μM). Precipitation of F-actin pulled down more Nlgn173 and p-Tyr-53 but less Arp2/3 in mCECs expressing circNlgn. (D) F/G-actin was fractionated from mCECs transfected with circNlgn siRNAs and treated with 100 μM DSS for 24 h. Silencing circNlgn increased F-actin levels in the nuclei. Immunoprecipitation with an antibody against actin coprecipitated Nlgn173. Silencing circNlgn decreased the precipitated amount. (E) Immunoprecipitation with an antibody against actin precipitated increased nuclear F-actin by silencing circNlgn. At an equal loading amount of precipitated F-actin, the levels of threonine and serine phosphorylation did not change, while phospho-tyrosine levels decreased in mCECs transfected with circNlgn siRNAs, which also showed decreased precipitation of Nlgn173 and p-Tyr-53 but more Arp2/3 by nuclear F-actin pulldown. (F) F-actin precipitation with 20 μl of biotin–phalloidin (50 μM) precipitated less Nlgn173 and p-Tyrosine but more Arp2/3 in mCECs transfected with circNlgn siRNAs. (G) Mouse colonic mucosa isolated from WT and circNlgn(+) mice treated with 1.5% DSS and 2% TNBS were subjected to nuclear fractionation and immunoprecipitation with biotin–phalloidin. Expression of circNlgn decreased F-actin levels in the nuclei, which was promoted by DSS and TNBS treatment. At an equal amount of the precipitated products, F-actin pulled down more Nlgn173 and p-Tyr-53 but less Arp2/3 in the circNlgn(+) mucosa, which was promoted by DSS and TNBS treatment. (H) Mucosa isolated from mice delivered with circNlgn siRNAs and mixmer treated with 3% DSS were subjected to nuclear fractionation and immunoprecipitation with biotin–phalloidin. Increased F-actin was detected in the nuclei when Nlgn173 decreased, which was promoted by DSS treatment. At equal amount of F-actin, precipitation of F-actin pulled down less Nlgn173 and p-Tyr-53 but more Arp2/3 when Nlgn173 levels decreased, which was enhanced by DSS treatment.

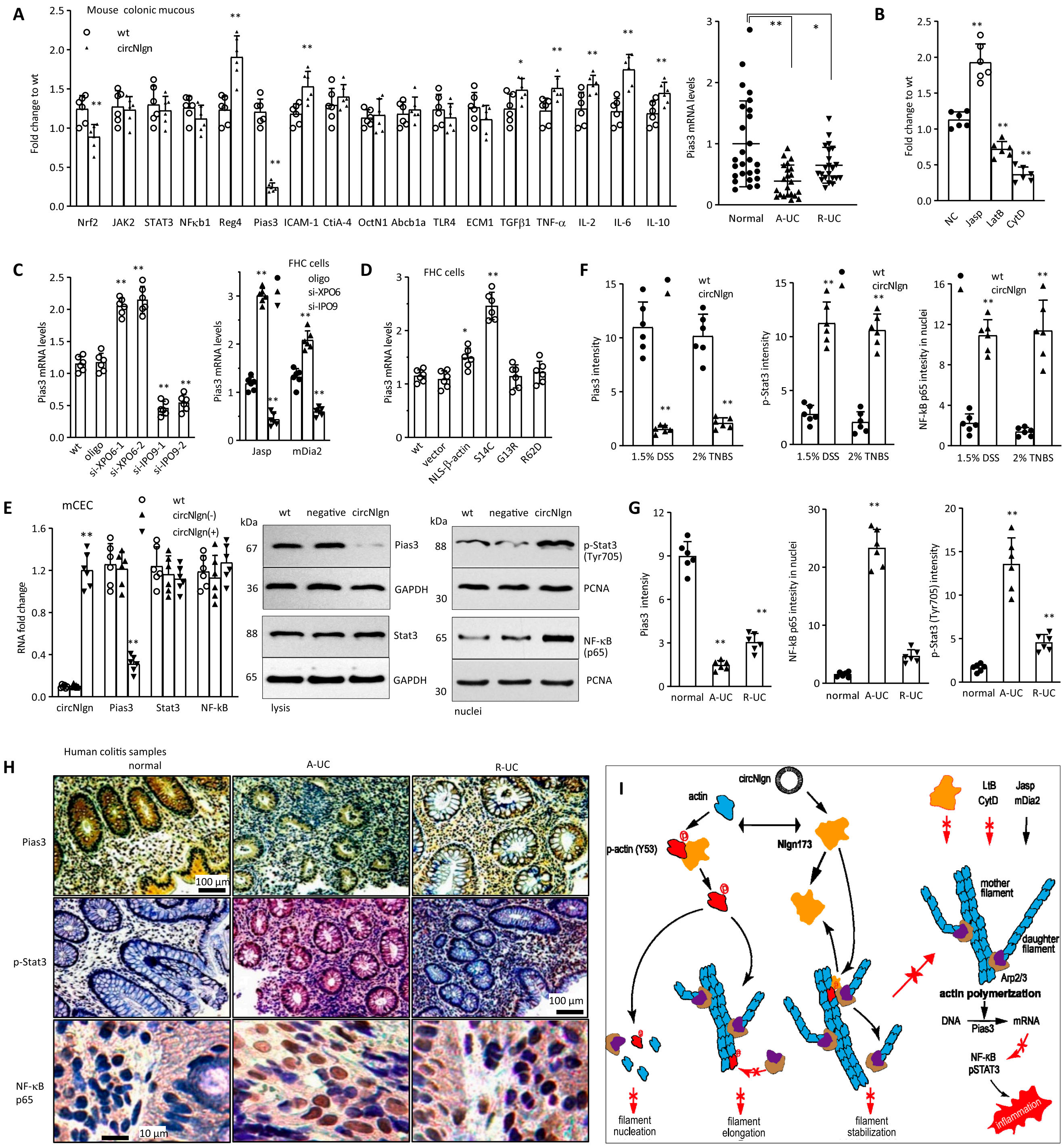

Fig. 9. Nlgn173 decreases PIAS3 expression but activates STAT3 and NF-κB. (A) Left: Mouse colonic mucosa was lysed and subjected to RT-PCR to assess mRNA levels of 17 colitis-related genes. **P* < 0.05, ***P* < 0.01 versus WT (*n* = 6). Right: Human colonic mucosa with A-UC and R-UC was lysed and subjected to RT-PCR. Both A-UC and R-UC mucosa expressed lower levels of PIAS3 than the normal tissues. ***P* < 0.01 versus normal (normal, *n* = 26; A-UC and R-UC, *n* = 22). (B) mCEC cells were cultured in 0.1 μM Jasp, 0.05 μM LatB," and 1 μM CytD for 24 h, followed by RT-PCR. mCEC cells showed increased PIAS3 levels when treated with Jasp but decreased PIAS3 levels when treated with LatB and CytD. **P* < 0.05, ***P* < 0.01 versus NC (*n* = 6). (C) Left: mCEC cells were transfected with XPO6 and IPO9 siRNAs and cultured in 0.1 μM Jasp for 24 h. RT-PCR showed that silencing XPO6 up-regulated, while silencing IPO9 down-regulated expression of PIAS3. Right: Colonic epithelial cell line FHC was transfected with XPO6 and IPO9 siRNAs and cultured in 0.1 μM Jasp for 24 h or cotransfected with mDia2. RT-PCR showed that silencing XPO6 up-regulated, while silencing IPO9 down-regulated expression of PIAS3. ***P* < 0.01 versus oligo (*n* = 6). (D) FHC cells were transfected with control vector, YFP-NLS-β-actin (NLS-β-actin), YFP-NLS-β-actin S14C (S14C), YFP-NLS-β-actin G13R (G13R), and NLS-β-actin R62D (R62D) mCherry. Expression of S14C increased PIAS3 levels. ***P* < 0.01 versus vector (*n* = 6). (E) Left: mCEC cells were isolated from WT and circNlgn-transgenic mice. circNlgn(+) eCEC cells showed high levels of circNlgn and decreased PIAS3. ***P* < 0.01 versus WT (*n* = 6). Right: Western blot showed decreased expression of PIAS3 but activation of STAT3 and NFκB. (F) ImageJ analysis showed that circNlgn-transgenic mouse colon tissues expressed low levels of PIAS3, and activation of STAT3 and NF-κB-p65. ***P* < 0.01 versus WT (*n* = 6). (G) Typical images showing immunohistochemical staining of PIAS3, p-STAT3, and NF-κB-p65 in human colonic mucosa of A-UC and R-UC. (H) ImageJ analysis showed that A-UC tissues expressed lower levels of PIAS3, high levels of p-STAT3, and high nuclear levels of NF-κB-p65. ***P* < 0.01 versus normal (*n* = 6). (I) A diagram showing that circNlgn promoted A-UC via suppressing nuclear actin polymerization.

Nuclear actin is also involved in DNA repair, where it is accumulated at sites of DNA damage that plays crucial roles in cell apoptosis [32]. It helps to recruit DNA repair factors and stabilize the repair complexes [33–36]. Disruption of nuclear F-actin impairs the repair process of DNA damage. Thus, nuclear actin, by forming nuclear F-actin and regulating nuclear organization, plays crucial roles in maintaining genome stability. Our study showed that the newly identified nuclear protein Nlgn173 encoded by the circRNA circNlgn inhibited the formation of F-actin in the nuclei. This has resulted in enhancement of colitis development and progression. Silencing expression of the circRNA circNlgn/Nlgn173 reversed the processes. Since circNlgn/Nlgn173 was highly up-regulated in the mouse colitis model and in patients with colitis, targeting circNlgn/Nlgn173 may be of potential application for intervention of the disease. CircRNAs have been reported to play crucial roles in regulating progression of different diseases [37–41]. Many cardio-specific circRNAs are known to modulate cardiac disorders including cardiac hypertrophy, cardiomyopathy, remodeling, artery diseases, and myocardial infarction [42–44]. CircRNAs may function through different mechanisms including protein translation [45]. A number of approaches have been developed to target circRNAs aiming to modulate disease progression [46–49]. In current study, we used mixmer to block circNlgn translation. This method would serve as a novel approach to target protein translation from circRNAs.

Similar to its presence in the cytoplasm, there are 2 forms of nuclear actin, namely, G-actin and F-actin. Nuclear actin polymerization refers to the process by which G-actin forms F-actin. This process plays essential roles in tissue repair by regulating gene expression involved in different cellular processes including cell migration, proliferation, differentiation, and tissue repair. Our recent study showed that nuclear actin polymerization regulated cellular epithelial–mesenchymal transition [25]. Here, we showed that the protein Nlgn173 encoded by circNlgn interacted with actin and inhibited the formation of F-actin. The binding of Nlgn173 to actin blocked the accessibility of Arp2/3, leading to inhibition of actin polymerization.

Arp2/3 complex is one of the key players in actin polymerization that binds to the sides of pre-existing actin filaments and facilitate the formation of new filaments [50]. The activated Arp2/3 complex can also bind G-actin to initiate the formation of filament branches. It is known that the activity of the complex can be modulated by various factors, including actin-binding proteins, molecules in the related signaling pathways, and posttranslational modifications such as phosphorylation [21,51,52]. Phosphorylation is a crucial posttranslational modification that plays a critical role in regulating many cellular processes including actin polymerization [53,54]. Phosphorylation of actin can regulate the stability of actin filaments, enhancing the overall structure and organization of the actin cytoskeleton. In our study, we found that Nlgn173 regulated actin Tyr-53 phosphorylation and repressed actin binding to the Arp2/3 complex. Tyr-53 phosphorylation could inhibit nucleation and actin filament elongation [24]. This is in agreement with other reports that phosphorylation of profilin, a protein that enhances actin filament involving interaction with the Arp2/3 complex, can also influence actin polymerization [55,56]. Phosphorylation of the actin-binding proteins can modify their interactions with actin, leading to alterations in actin filament assembly. While increased levels of Nlgn173 promoted actin phosphorylation and silencing Nlgn173 expression decreased actin phosphorylation, it is not clear how it occurred: it appeared that the interaction with Nlgn173 presented actin to be phosphorylated by related kinases. Due to the different environments, the mechanism of actin polymerization may be different between nuclei and cytosol. The specific interaction of Nlgn173 and actin may serve as the molecular basis for understanding actin polymerization in the nuclei.

Another important aspect of nuclear actin polymerization may be its impact on inflammation. Some studies report that nuclear F-actin plays roles in regulating expression of inflammation-associated genes. This could be due to the involvement of F-actin in modulating chromatin structure and gene expression through binding to various chromatin binding proteins, transcription factors, and RNA polymerases. It is reported that disruption of F-actin assembly in the nuclei decreased expression of proinflammatory cytokines such as interleukin-6 and tumor necrosis factor α (TNFα), suggesting that nuclear F-actin may promote inflammation [57]. However, other studies showed that nuclear F-actin may have anti-inflammatory effects: increasing nuclear F-actin levels decreased the expression of adhesion molecules and proinflammatory cytokines, suggesting decreased inflammation by nuclear F-actin [58–60]. Nevertheless, the exact effect of nuclear actin polymerization on proinflammatory gene expression associated with colitis development is not well understood. It may vary depending on the specific context of colitis.

Colitis is a chronic inflammatory disease that affects the colon and rectum. While the exact causes of colitis are not fully understood, it is believed to result from an abnormal immune response. Abnormal immune response can affect both A-UC and R-UC, but its impact on A-UC and R-UC may be different. In A-UC, the primary goal is to reduce inflammation and promote healing of the intestinal lining by inducing remission and alleviating symptoms. In R-UC, the disease often flares up after a period of remission, and management of the disease involves maintenance medications to prevent relapses that keep R-UC in remission. Thus, the effects of different factors on colitis progression may be different at the cellular and molecular levels. For instance, cell proliferation may be an essential step in A-UC recovery but may not be necessary in R-UC. Epithelial–mesenchymal transition may help in the recovery of A-UC but not in R-UC. Our study discovered that decreased nuclear actin polymerization promoted A-UC progression but had less effect on R-UC. This suggests that nuclear actin polymerization and its associated inflammatory molecules are helpful in recovery of A-UC but not in R-UC. This is in agreement with previous studies showing that disruption of nuclear actin polymerization can lead to an increase in the expression of proinflammatory genes that contributes to the development of colitis [5,61,62]. Additionally, nuclear actin polymerization may serve as an intestinal barrier for the maintenance of intestinal structure, preventing harmful substances entering the body. Decreased nuclear actin polymerization in A-UC patients and in the mouse colitis model disrupted the intestinal homeostasis and lead to increased intestinal permeability and repressed intestinal mucosal repairing in A-UC. Strategies such as circNlgn knockdown targeting nuclear actin polymerization of colonic epithelium may explore a new avenue for A-UC clinical intervention.

Materials and Methods

The general methods were performed as previously described [63–67]. The details for these sections are provided in the Supplementary Materials.

Human colon specimens

The study was conducted following the guidelines of The Ethics Code of the World Medical Association (Declaration of Helsinki). Patients included in this study provided formal informed consent before enrollment. For Fig. 1A to C, colon samples were obtained from 30 patients with A-UC and 30 individuals with records of R-UC by mucosal biopsy. Normal colons without detectable colitis were collected from 30 individuals who did not have UC. All the above colon biopsy samples were collected from sigmoid colon. In Fig. 1D, human colonic mucosa was collected from inflamed and unaffected areas of 18 cases of A-UC cases subjected to colon surgery due to colitis. The protocol was reviewed and approved by the Ethics Committee in The Second Affiliated Hospital of Guangzhou Medical University (2022-KY-ks-08).

At the time of surgery or biopsy, colonic mucosal tissue was removed, divided into portions, and processed as follows: the first fragment was fixed in formaldehyde (4%) for 2 to 3 d and then embedded in wax. The second part was collected in cryovials, snap-frozen in liquid nitrogen, stored at −80 °C, and used for RNA or protein isolation. The remaining fragment was embedded for frozen sections.

Constructs, siRNAs, and primers

The plasmids of circNlgn, linNlgn (the translation fragment expressed by the pcDNA3.1 plasmid that does not form circRNA), circNlgn-mut (circ-mut, circNlgn containing a point mutation to disrupt protein translation), circNlgn164 (circ-164, circNlgn containing a point mutation to avoid addition of the 9 amino acids as a result of back-splicing), and circNlgn precursor were generated by Gene Universal. The vector plasmid contains a Bluescript backbone, a human cytomegalovirus (CMV) promoter that drives expression of green fluorescent protein, and a second cytomegalovirus promoter that drives the circRNA-forming fragments or a nonrelated sequence serving as a mock control. The plasmid containing full-length human Nlgn gene was obtained from Addgene. The sequences of the primers and siRNAs used are listed in the Tables S1 and S2.

F-actin/G-actin fractionation

We used the F-actin/G-actin in vivo assay kit (BK037) to isolate F- and G-actin fractions. Briefly, cells or isolated nuclei were lysed with F-actin stabilization buffer 2 (LAS2), homogenized with 25-G syringe 20 times, and incubated at 37 °C for 10 min. After centrifuged (2,000 rpm, 5 min) to pellet the unbroken cells, the supernatant was centrifuged again (100,000xg) at 37 °C for 1 h. After centrifugation, F-actin and its binding proteins were in the pellet, while G-actin stayed in the supernatant.

Identification of actin-binding proteins

An actin-binding protein biochem kit (BK001) was used to identify actin-binding proteins. Briefly, 40 μl of G-actin and F-actin were prepared as the manual described. Tested protein (10 μl, 2 μM) was added to the mixture of F/G-actin and incubated at room temperature for 30 min. After centrifuged at 150,000xg for 1.5 h at 24 °C, the pellet contained F-actin and its binding proteins, while the supernatant contained G-actin, which could be detected by Western blot analysis. Whether the supernatant contained protein bound to G-actin or not should be confirmed by following actin precipitation assay in "Identification of G-actin-binding proteins".

Identification of G-actin-binding proteins

G-actin-binding proteins were identified by immunoprecipitating G-actin with a monoclonal antibody against actin. To elaborate, magnetic beads (100 μl) were washed in phosphate buffered saline with Tween 20 (PBS-T), followed by incubation with antibody against actin (5 μg) at room temperature for 10 min. The antibody-conjugated beads were then washed with PBS-T 3 times and incubated with LAS2-lysed G-actin extracts for 1 h. The magnetic beads were washed 3 times with PBS-T and resuspended in 2× Laemmli buffer (0.125 M tris-HCl, 4% SDS, 20% glycerol, 10% 2-mercaptoethanol, and 0.004% bromophenol blue, pH 6.8), followed by Western blotting.

Identification of F-actin-binding proteins

F-actin-binding proteins were confirmed through the immunoprecipitation of F-actin with a monoclonal antibody against actin. In this process, the actin antibody-conjugated magnetic beads were incubated with LAS2-lysed F-actin extracts for 1 h, washed 3 times with PBS-T, and then resuspended in 2× Laemmli buffer, followed by Western blot analysis.

Phalloidin was used to immunoprecipitate F-actin and its binding proteins. Briefly, cells or tissues were resuspended in LAS2 (500 μl) and incubated with 20 μl of biotinylated-phalloidin (B7474) at 37 °C for 1 h. Then, 50 μl Dynabeads MyOne Streptavidin C1 (65002) was added to the mixture and incubated at 37 °C for 30 min. The beads were washed with LAS2, and the pull-down proteins were assayed by Western blot.

Actin polymerization assay

The actin polymerization was conducted using an actin polymerization biochemistry kit (BK 003). To begin, pyrene actin was dissolved in G-buffer containing adenosine triphosphate at 0.4 mg/ml and incubated on ice for 1 h. After centrifuged (14,000 rpm) at 4°C for 30 min, the supernatant was placed in a 96-well plate with 200 μl per well. Meantime, the test cells or tissues were lysed in 20 mM Hepes with 20 mM NaCl. The mixture was subjected to centrifugation at 150,000×g at 4°C for 1 h. Subsequently, 20 μl of the supernatant that contained G-actin was added in each well. Actin polymerization occurred when the buffer was added to the wells. Multiscan Spectrum Reader (BioTek Synergy H1) was used to detect actin polymerization for 120 cycles with 60-s interval time.

Analysis of nuclear F/G-actin in colonic mucosa

Colon sections were de-paraffinized with xylene and ethanol. The sections were then washed with tris-buffered saline (TBS) containing 0.025% Triton X-100 and blocked with 10% goat serum and incubated with 1:3,000 Alexa Fluor 488–conjugated Deoxyribonuclease I (D12371) in TBS that contained 10% goat serum at 4 °C overnight. After being washed with TBS, the sections were incubated with 1:100 diluted Alexa Fluor 555 Phalloidin (A34055) and DAPI for 30 min.

The images of the staining sections were captured using Nikon N-SIM S confocal laser scanning microscopy. The intensity of Phalloidin (F-actin)/Deoxyribonuclease I staining within the nucleus was analyzed using ImageJ. Phalloidin/

Deoxyribonuclease I staining areas overlapping with 4′,6-diamidino-2-phenylindole (DAPI) were defined as nuclear F-actin/G-actin-stained. The average value of 5 cells from each image represented the intensity of F-actin/G-actin. Phalloidin/Deoxyribonuclease I staining around the edge of the nucleus was excluded. Only the staining away from the nuclear edge was counted as nuclear F-actin/G-actin-positive staining.

circNlgn-transgenic mice

When performing mouse experiments, we followed guidelines approved by the Animal Care Committee of Sunnybrook Research Institute (protocol ID: AUP#22-244). The transgenic mice expressing circNlgn were generated in C57BL/6J by pronuclear microinjection of DNA fragment expressing circNlgn, performed by the Toronto Centre for Phenogenomics. All transgenic mice were ear-tagged and processed to genotyping after weaning. The primer sequences used for genotyping are listed in Table S1.

Induction of acute colitis model

Colitis was induced by administering DSS (1.5% to 3%, molecular weight: 36,000 to 50,000, MP Biomedicals, Solon, OH, USA) into mice for 7 d followed by a return to DSS-free water for 3 d. TNBS (Sigma-Aldrich, St. Louis, MO, USA) colitis was induced by a single colonic enema (2.0% to 2.5% in 100 μl of 50% ethanol).

Histological damage score

Colonic tissue sections stained with H&E were used for histological assessment of colitis. Two slides were scored for each experimental group by 3 observers blinded to the treatment groups, using previously described criteria [68]: 0 showed no signs of inflammation; 1 indicated very low level of leukocyte infiltration; 2 represented low level of leukocyte infiltration; 3 stood for high level of leukocyte infiltration, high vascular density, and thickening of the colon wall; and 4 showed transmural infiltration, loss of goblet cells, high vascular density, and thickening of the colon wall.

Statistical analysis

Data were provided as mean (bar) with SD (whisker). For multiple group assays, a one-way analysis of variance (ANOVA) was conducted, followed by a Bonferroni post hoc test for one independent variable, and a 2-way ANOVA followed by a Bonferroni correction for 2 independent variables. A 2-tailed unpaired Student *t* test was performed to assess the differences between 2 groups with a single independent factor. All in vitro experiments were repeated at least 3 times, unless otherwise specified. Kaplan–Meier survival test was used to analyze the survival difference among groups. Prism 8 (GraphPad Software, La Jolla, CA) was used for the statistical analyses, with differences considered statistically significant when the nominalized *P* value was less than 0.05.

Ethical Approval

All animal experiments were conducted in accordance with the relevant guidelines and regulations approved by the Animal Care Committee of Sunnybrook Research Institute. The approval numbers for animal experiments are AUP#2004-244 to 2022-224 (renewed every year).

Acknowledgments

Funding: This work was supported by the grants from Canadian Institutes of Health Research (PJT-155962 and PJT-166107) to B.B.Y.
Author contributions: B.B.Y. supervised the project. W.W.D. designed the experiments. W.W.D., C.Z., H. Yang., S.W., Y.C., F.L., K.Y.D., H. Yuan, E.X.C., T.Y., and J.Q.. performed the experiments. S.W. performed computational analysis of circRNA interaction. W.W.D. and B.B.Y. analyzed the data. W.W.D. and B.B.Y. wrote the paper.
Competing interests: The authors declare that they have no competing interests.

Data Availability

The data underlying this article will be shared on reasonable request to the corresponding author.

Supplementary Materials

Supplementary Materials and Methods
Figs. S1 to S17
Tables S1 and S2

References

1. Ray K. Inflammation: Colitis, microbiota and malnutrition. *Nat Rev Gastroenterol Hepatol*. 2012;9(9):490.
2. Lima SF, Gogokhia L, Viladomiu M, Chou L, Putzel G, Jin WB, Pires S, Guo CJ, Gerardin Y, Crawford CV, et al. Transferable immunoglobulin a-coated Odoribacter splanchnicus in responders to fecal microbiota transplantation for ulcerative colitis limits colonic inflammation. *Gastroenterology*. 2022;162(1):166–178.
3. Davrandi M, Harris S, Smith PJ, Murray CD, Lowe DM. The relationship between mucosal microbiota, colitis, and systemic inflammation in chronic granulomatous disorder. *J Clin Immunol*. 2022;42(2):312–324.
4. Tang X, Wang W, Hong G, Duan C, Zhu S, Tian Y, Han C, Qian W, Lin R, Hou X. Gut microbiota-mediated lysophosphatidylcholine generation promotes colitis in intestinal epithelium-specific fut2 deficiency. *J Biomed Sci*. 2021;28(1):20.
5. Lechuga S, Naydenov NG, Feygin A, Cruise M, Ervasti JM, Ivanov AI. Loss of beta-cytoplasmic actin in the intestinal epithelium increases gut barrier permeability in vivo and exaggerates the severity of experimental colitis. *Front Cell Dev Biol*. 2020;8:Article 588836.
6. Rodriguez-Boulan E, Macara IG. Organization and execution of the epithelial polarity programme. *Nat Rev Mol Cell Biol*. 2014;15(4):225–242.
7. Pollard TD, Cooper JA. Actin, a central player in cell shape and movement. *Science*. 2009;326(5957):1208–1212.
8. Geiger B, Spatz JP, Bershadsky AD. Environmental sensing through focal adhesions. *Nat Rev Mol Cell Biol*. 2009;10 (1):21–33.
9. Kapoor P, Shen X. Mechanisms of nuclear actin in chromatin-remodeling complexes. *Trends Cell Biol*. 2014;24(4):238–246.
10. Posern G, Sotiropoulos A, Treisman R. Mutant actins demonstrate a role for unpolymerized actin in control of transcription by serum response factor. *Mol Biol Cell*. 2002;13(12):4167–4178.

11. Chen M, Shen X. Nuclear actin and actin-related proteins in chromatin dynamics. *Curr Opin Cell Biol*. 2007;19(3): 326–330.
12. Sokolova M, Moore HM, Prajapati B, Dopie J, Merilainen L, Honkanen M, Matos RC, Poukkula M, Hietakangas V, Vartiainen MK. Nuclear actin is required for transcription during Drosophila oogenesis. *iScience*. 2018;9:63–70.
13. Caridi CP, D'Agostino C, Ryu T, Zapotoczny G, Delabaere L, Li X, Khodaverdian VY, Amaral N, Lin E, Rau AR, et al. Nuclear f-actin and myosins drive relocalization of heterochromatic breaks. *Nature*. 2018;559(7712):54–60.
14. Schrank BR, Aparicio T, Li Y, Chang W, Chait BT, Gundersen GG, Gottesman ME, Gautier J. Nuclear ARP2/3 drives DNA break clustering for homology-directed repair. *Nature*. 2018;559(7712):61–66.
15. Plessner M, Grosse R. Dynamizing nuclear actin filaments. *Curr Opin Cell Biol*. 2019;56:1–6.
16. Viita T, Kyheroinen S, Prajapati B, Virtanen J, Frilander MJ, Varjosalo M, Vartiainen MK. Nuclear actin interactome analysis links actin to KAT14 histone acetyl transferase and mrna splicing. *J Cell Sci*. 2019;132(8):Article jcs226852.
17. Le HQ, Ghatak S, Yeung CY, Tellkamp F, Gunschmann C, Dieterich C, Yeroslaviz A, Habermann B, Pombo A, Niessen CM, et al. Mechanical regulation of transcription controls polycomb-mediated gene silencing during lineage commitment. *Nat Cell Biol*. 2016;18(8):864–875.
18. Figard L, Zheng L, Biel N, Xue Z, Seede H, Coleman S, Golding I, Sokac AM. Cofilin-mediated actin stress response is maladaptive in heat-stressed embryos. *Cell Rep*. 2019;26(13):3493–3501.
19. Du WW, Xu J, Yang W, Wu N, Li F, Zhou L, Wang S, Li X, He AT, Du KY, et al. A neuroligin isoform translated by circNlgn contributes to cardiac remodeling. *Circ Res*. 2021;129(5):568–582.
20. Xu J, Du WW, Wu N, Li F, Li X, Xie Y, Wang S, Yang BB. The circular RNA circNlgnmediates doxorubicin-inducedcardiac remodeling and fibrosis. *Mol Ther Nucleic Acids*. 2022;28: 175–189.
21. Rocca DL, Martin S, Jenkins EL, Hanley JG. Inhibition of Arp2/3-mediated actin polymerization by PICK1 regulates neuronal morphology and AMPA receptor endocytosis. *Nat Cell Biol*. 2008;10(3):259–271.
22. Thiam HR, Vargas P, Carpi N, Crespo CL, Raab M, Terriac E, King MC, Jacobelli J, Alberts AS, Stradal T, et al. Perinuclear ARP2/3-driven actin polymerization enables nuclear deformation to facilitate cell migration through complex environments. *Nat Commun*. 2016;7:10997.
23. Chen X, Li Y, Guo M, Xu B, Ma Y, Zhu H, Feng XQ. Polymerization force-regulated actin filament-ARP2/3 complex interaction dominates self-adaptive cell migrations. *Proc Natl Acad Sci USA*. 2023;120(36):Article e2306512120.
24. Liu X, Shu S, Hong MS, Levine RL, Korn ED. Phosphorylation of actin Tyr-53 inhibits filament nucleation and elongation and destabilizes filaments. *Proc Natl Acad Sci USA*. 2006;103(37):13694–13699.
25. Du WW, Qadir J, Du KY, Chen Y, Wu N, Yang BB. Nuclear actin polymerization regulates cell epithelial-mesenchymal transition. *Adv Sci*. 2023;10(28):Article e2300425.
26. Ma J, Yang Y, Fu Y, Guo F, Zhang X, Xiao S, Zhu W, Huang Z, Zhang J, Chen J. Pias3-mediated feedback loops promote chronic colitis-associated malignant transformation. *Theranostics*. 2018;8(11):3022–3037.
27. Goldstein L, Ko C, Errick J. Nuclear actin: An apparent association with condensed chromatin. *Cell Biol Int Rep*. 1977;1(6):511–515.
28. Wang Y, Sherrard A, Zhao B, Melak M, Trautwein J, Kleinschnitz EM, Tsopoulidis N, Fackler OT, Schwan C, Grosse R. GPCR-induced calcium transients trigger nuclear actin assembly for chromatin dynamics. *Nat Commun*. 2019;10(1):5271.
29. Knoll KR, Eustermann S, Niebauer V, Oberbeckmann E, Stoehr G, Schall K, Tosi A, Schwarz M, Buchfellner A, Korber P, et al. The nuclear actin-containing arp8 module is a linker DNA sensor driving ino80 chromatin remodeling. *Nat Struct Mol Biol*. 2018;25(9):823–832.
30. Baarlink C, Plessner M, Sherrard A, Morita K, Misu S, Virant D, Kleinschnitz EM, Harniman R, Alibhai D, Baumeister S, et al. A transient pool of nuclear f-actin at mitotic exit controls chromatin organization. *Nat Cell Biol*. 2017;19(12):1389–1399.
31. Szerlong H, Hinata K, Viswanathan R, Erdjument-Bromage H, Tempst P, Cairns BR. The HSA domain binds nuclear actin-related proteins to regulate chromatin-remodeling ATPases. *Nat Struct Mol Biol*. 2008;15(5):469–476.
32. Ma Y, Cao L, Kawabata T, Yoshino T, Yang BB, Okada S. Cupric nitrilotriacetate induces oxidative DNA damage and apoptosis in human leukemia HL-60 cells. *Free Radic Biol Med*. 1998;25(4-5):568–575.
33. Szerlong H, Saha A, Cairns BR. The nuclear actin-related proteins Arp7 and Arp9: A dimeric module that cooperates with architectural proteins for chromatin remodeling. *EMBO J*. 2003;22(12):3175–3187.
34. Stanczyk P, Tatekoshi Y, Shapiro JS, Nayudu K, Chen Y, Zilber Z, Schipma M, De Jesus A, Mahmoodzadeh A, Akrami A, et al. DNA damage and nuclear morphological changes in cardiac hypertrophy are mediated by SNRK through actin depolymerization. *Circulation*. 2023;148(20):1582–1592.
35. Zagelbaum J, Schooley A, Zhao J, Schrank BR, Callen E, Zha S, Gottesman ME, Nussenzweig A, Rabadan R, Dekker J, et al. Multiscale reorganization of the genome following DNA damage facilitates chromosome translocations via nuclear actin polymerization. *Nat Struct Mol Biol*. 2023;30(1):99–106.
36. Hurst V, Shimada K, Gasser SM. Nuclear actin and actin-binding proteins in DNA repair. *Trends Cell Biol*. 2019;29(6):462–476.
37. Du WW, Yang W, Li X, Fang L, Wu N, Li F, Chen Y, He Q, Liu E, Yang Z, et al. The circular RNA circSKA3 binds integrin β1 to induce invadopodium formation enhancing breast cancer invasion. *Mol Ther*. 2020;28:1287–1298.
38. Qadir J, Li F, Yang BB. Circular RNAs modulate hippo-yap signaling: Functional mechanisms in cancer. *Theranostics*. 2022;12(9):4269–4287.
39. Yang Q, Li F, He AT, Yang BB. Circular RNAs: Expression, localization, and therapeutic potentials. *Mol Ther*. 2021;29(5):1683–1702.
40. Li F, Yang Q, He AT, Yang BB. Circular RNAs in cancer: Limitations in functional studies and diagnostic potential. *Semin Cancer Biol*. 2021;75:49–61.
41. Qadir J, Wen SY, Yuan H, Yang BB. CircRNAs regulate the crosstalk between inflammation and tumorigenesis: The bilateral association and molecular mechanisms. *Mol Ther*. 2023;31(6):1514–1532.
42. Wang L, Yu P, Wang J, Xu G, Wang T, Feng J, Bei Y, Xu J, Wang H, Das S, et al. Downregulation of circ-ZNF609 promotes

heart repair by modulating rna N^6-methyladenosine-modified yap expression. *Research*. 2022;2022:9825916.
43. Wang L, Xu GE, Spanos M, Li G, Lei Z, Sluijter JPG, Xiao J. Circular RNAs in cardiovascular diseases: Regulation and therapeutic applications. *Research*. 2023;6:0038.
44. Wu N, Xu J, Du WW, Li X, Awan FM, Li F, Misir S, Eshaghi E, Lyu J, Zhou L, et al. Yap circular RNA, circYap, attenuates cardiac fibrosis via binding with tropomyosin-4 and gamma-actin decreasing actin polymerization. *Mol Ther*. 2021;29(3):1138–1150.
45. Wen SY, Qadir J, Yang BB. Circular RNA translation: Novel protein isoforms and clinical significance. *Trends Mol Med*. 2022;28(5):405–420.
46. Wu N, Qadir J, Yang BB. CircRNA perspective: New strategies for rna therapy. *Trends Mol Med*. 2022;28(4):343–344.
47. Ma J, Du WW, Zeng K, Wu N, Fang L, Lyu J, Yee AJ, Yang BB. An antisense circular RNA circSCRIB enhances cancer progression by suppressing parental gene splicing and translation. *Mol Ther*. 2021;29(9):2754–2768.
48. Ren S, Huang M, Bai R, Chen L, Yang J, Zhang J, Guo W, Ji W, Chen Y. Efficient modulation of exon skipping via antisense circular RNAs. *Research*. 2023;6:0045.
49. Wu N, Li F, Yang W, Du WW, Awan FM, Zhang C, Lyu J, Misir S, Zeng K, Eshaghi E, et al. Silencing mouse circular RNA circSlc8a1 by circular antisense cA-circSlc8a1 induces cardiac hepatopathy. *Mol Ther*. 2023;31(6):1688–1704.
50. To C, Shilton BH, Di Guglielmo GM. Synthetic triterpenoids target the Arp2/3 complex and inhibit branched actin polymerization. *J Biol Chem*. 2010;285(36):27944–27957.
51. Bogucka-Janczi K, Harms G, Coissieux MM, Bentires-Alj M, Thiede B, Rajalingam K. ERK3/MAPK6 dictates CDC42/RAC1 activity and ARP2/3-dependent actin polymerization. *elife*. 2023;12:Article e85167.
52. Zhao K, Wang D, Zhao X, Wang C, Gao Y, Liu K, Wang F, Wu X, Wang X, Sun L, et al. WDR63 inhibits Arp2/3-dependent actin polymerization and mediates the function of p53 in suppressing metastasis. *EMBO Rep*. 2020;21(4):Article e49269.
53. Kim Y, Sung JY, Ceglia I, Lee KW, Ahn JH, Halford JM, Kim AM, Kwak SP, Park JB, Ho Ryu S, et al. Phosphorylation of wave1 regulates actin polymerization and dendritic spine morphology. *Nature*. 2006;442(7104):814–817.
54. Eiseler T, Hausser A, De Kimpe L, Van Lint J, Pfizenmaier K. Protein kinase D controls actin polymerization and cell motility through phosphorylation of cortactin. *J Biol Chem*. 2010;285(4):18672–18683.
55. Yu M, Le S, Efremov AK, Zeng X, Bershadsky A, Yan J. Effects of mechanical stimuli on profilin- and formin-mediated actin polymerization. *Nano Lett*. 2018;18(8):5239–5247.
56. Liu X, Qu X, Jiang Y, Chang M, Zhang R, Wu Y, Fu Y, Huang S. Profilin regulates apical actin polymerization to control polarized pollen tube growth. *Mol Plant*. 2015;8(12): 1694–1709.
57. Phusuntornsakul P, Jitpukdeebodintra S, Pavasant P, Leethanakul C. Vibration activates the actin/NF-κB axis and upregulates IL-6 and IL-8 expression in human periodontal ligament cells. *Cell Biol Int*. 2020;44:661–670.
58. Tonsawan P, Dylewski J, Lewis L, Blaine J. Knockout of the neonatal Fc receptor in cultured podocytes alters IL-6 signaling and the actin cytoskeleton. *Am J Physiol Cell Physiol*. 2019;317:C1048–C1060.
59. Liang H, Yu F, Tong Z, Yuan B, Wang C. Vibration activates the actin/NF-κB axis and upregulates IL-6 and IL-8 expression in human periodontal ligament cells. *Mol Biol Rep*. 2013;40(2):507–514.
60. Georgieva E, Leber SL, Wex C, Garbers C. Perturbation of the actin cytoskeleton in human hepatoma cells influences interleukin-6 (IL-6) signaling, but not soluble IL-6 receptor generation or NF-κB activation. *Int J Mol Sci*. 2021;22(13):7171.
61. Ma Y, Yue J, Zhang Y, Shi C, Odenwald M, Liang WG, Wei Q, Goel A, Gou X, Zhang J, et al. ACF7 regulates inflammatory colitis and intestinal wound response by orchestrating tight junction dynamics. *Nat Commun*. 2017;8:15375.
62. Zhang C, Chen H, He Q, Luo Y, He A, Tao A, Yan J. Fibrinogen/AKT/microfilament axis promotes colitis by enhancing vascular permeability. *Cell Mol Gastroenterol Hepatol*. 2021;11:683–696.
63. LaPierre DP, Lee DY, Li SZ, Xie YZ, Zhong L, Sheng W, Deng Z, Yang BB. The ability of versican to simultaneously cause apoptotic resistance and sensitivity. *Cancer Res*. 2007;67(10):4742–4750.
64. Xu J, Bai XH, Lodyga M, Han B, Xiao H, Keshavjee S, Hu J, Zhang H, Yang BB, Liu M. Xb130, a novel adaptor protein for signal transduction. *J Biol Chem*. 2007;282(22):16401–16412.
65. Rutnam ZJ, Yang BB. The non-coding 3' UTR of CD44 induces metastasis by regulating extracellular matrix functions. *J Cell Sci*. 2012;125(Pt 8):2075–2085.
66. Xu G, Zhong Y, Munir S, Yang BB, Tsang BK, Peng C. Nodal induces apoptosis and inhibits proliferation in human epithelial ovarian cancer cells via activin receptor-like kinase 7. *J Clin Endocrinol Metab*. 2004;89(11):5523–5534.
67. Tian L, Song Z, Shao W, Du WW, Zhao LR, Zeng K, Yang BB, Jin T. Curcumin represses mouse 3T3-L1 cell adipogenic differentiation via inhibiting mir-17-5p and stimulating the Wnt signalling pathway effector Tcf7l2. *Cell Death Dis*. 2017;8(1):Article e2559.
68. Wang H, Chao K, Ng SC, Bai AH, Yu Q, Yu J, Li M, Cui Y, Chen M, Hu JF, et al. Pro-inflammatory miR-223 mediates the cross-talk between the IL23 pathway and the intestinal barrier in inflammatory bowel disease. *Genome Biol*. 2016;17:58.
