# Supplementary material for: Aggravated Ulcerative Colitis via circNlgn-Mediated Suppression of Nuclear Actin Polymerization
Source: Research (Wash D C). 2024 Aug 23;7:0441. doi: 10.34133/research.0441 (PMC11342054; doi:10.34133/research.0441)
Supplement: Supplementary 1 — Supplementary Materials and Methods Figs. S1 to S17 Tables S1 and S2 [file research.0441.f1.zip › colistis -Supplementary -July 3-2024.pdf]

Supplementary Materials

1. Supplementary Material and Methods (pages 1-3)
2. Supplementary Tables (page 4)
3. Supplementary Figures (pages 5-21)

1. Supplementary Material and Methods

Materials

Polyclonal antibody against Nlgn173 junction peptide (GYRPAANWI) was obtained from Genscript, and purified using histidine-tagged Nlgn173 bound Ni-NTA resins in our lab. Monoclonal antibody against Nlgn (N-terminal, SC-365087) was purchased from Santa cruz, and monoclonal antibody against Nlgn (C-terminal, #819001) was obtained from Biolegends (San Diego, CA, USA). Monoclonal antibodies against β-catenin (#8480), actin (#4968), PCNA (#13110), Tubulin (#2148) and GAPDH (#2118) were purchased from cell signaling (Danvers, MA, USA). Dextran sodium sulfate (DSS, MW: 36,000–50,000) was obatined from MP Biomedicals (Solon, OH, USA). Monoclonal antibodies against Arp2 (A8876) and Arp3 (A4514) were purchased from ABclone (Woburn, MA, USA). Alexa Fluor 555 Phalloidin (A34055), Alexa Fluor 488 Deoxyribonuclease I (D12371), 2,4,6-trinitrobenzene sulfonic acid (TNBS), 96-well ELISA high binding plate (MSEHNFX) and Western blot detection kit (C72652) were purchased from Millipore Sigma (Oakville, ON, Canada). Human actin protein (APHL99), Arp2/3 protein (RP01), WASP protein VCA domain (VCA, VCG03), G-actin/F-actin in vivo assay kit (BK037), actin polymerization biochem kit (BK003) and monoclonal antibody against Actin (AAN02) were obtained from Cytoskeleton (Denver, CO, USA). Polyclonal antibody against Actin (101273) were purchased from Sino Biological. polyclonal antibody against p-tyrosine-53 actin was purchased from ECM Bioscience (Versailles, KY, USA). Biotin-XX Phalloidin (B7474) and Dynabeads MyOne Streptavidin C1(65002) were obtained from Thermo Fisher Scientific (Waltham, MA, USA). RNA extract (7326820), RT (1725151) and PCR (1708880) kits, Horseradish peroxidase-conjugated goat anti-rabbit and anti-mouse IgG were from Bio-Rad (Hercules, CA, USA). Multi-Analyte ELIS Array Kits for cytokines were obtained from Qiagen (MEN 004A, Hilden, Germany).

Subcellular fraction

Cultured cells or tissues were harvested and resuspended in 500 µl fractionation buffer (20 mM HEPES pH 7.4, 250mM sucrose, 10 mM KCl, 2 mM MgCl2, 1 mM EDTA, 1 mM EGTA, and 1x Roche protease inhibitor cocktail). After homogenization by passing through a 25-G needle using a 2 ml syringe, the mixture was incubated on ice for 30 minutes. Following centrifugation at 720×g for 10 minutes, the pellet contained the nuclear fraction, and the supernatant contained the cytosol. The nuclear pellet was resuspended in 500 µl fractionation buffer and centrifuged at 720×g for 5 minutes after washing three times. The extracted nuclear pellet was used for experiments or stored frozen at -80° C.

Immunoprecipitation assays

Bio-Rad magnetic beads were used for immunoprecipitation assays. Briefly, 100 µl magnetic beads were washed in PBS-T (PBS + 0.1% Tween 20) and incubated with 5 µg primary antibody at room temperature for 20 min. Cells or tissues were lysed, and incubated with antibody-containing beads for 1.5 h. The magnetic beads were washed 3 times with PBS-T and resuspended in 2× Laemmli buffer (0.125 M Tris-HCl, 4% SDS, 20% glycerol, 10% 2-mercaptoethanol, 0.004% bromphenol blue, pH 6.8), followed by Western blot analysis.

Solid phase microplate protein binding assay

To assess the interaction among F-actin, Arp2/3 and Nlgn173, a solid phase microplate protein binding assay was as previously described [1]. Briefly, 100 µL Buffer A (100 mM KCl, 3 mM MgCl2, and 10 mM PIPES, pH 7.0) with 4 µg/ml Arp2/3 complex was used to coated added to each well of a 96-well ELISA high binding plate (MSEHNFX, Sigma) and incubated overnight at 4 ° C. The plates were then washed with Buffer A+ 0.1% Tween-20, followed by blocking (5 % non-fat dry milk/Buffer A) for 1 h. After wash, 100 µL blocking buffer with prepared F-actin (20 µg/ml) and Nlgn173 (4 µg/ml) were loaded and incubated for 1 h at 37 ° C. After washed, mouse monoclonal antibodies against actin (1:500-2000) were added and incubated for 2 h, followed by goat anti-mouse second antibody (HRP,1:4000) for 2h. The plates were washed and incubated with TMB Substrate Solution (Thermo Scientific) for 30 min. The reaction was stopped with 2 M H2SO4, and read the optical density at 450 nm.

Western blotting

Cells or tissues were lysed and subjected to sodium dodecyl sulfate-polyacrylamide gel electrophoresis (SDS-PAGE) containing 5-12% acrylamide. Transblotting was processed onto a nitrocellulose membrane in 1 x Tris/glycine buffer containing 20% methanol at 70-V at 4° C for 1.5 -2 h. The membrane was blocked in a washing buffer (10 mM Tris-Cl, pH 8.0, 150 mM NaCl, 0.05% Tween-20) containing 5% non-fat dry milk powder for 30 min, and then incubated with primary antibodies at 4° C overnight. The membranes were washed with above washing buffer 3 ×25 min and then incubated with secondary antibodies for 1.5 h. After washing with washing buffer 3×25 min, the bound antibodies were visualized with an ECL detection kit.

RT-PCR and real-time PCR

To analyze gene expression, tissues or cells were lysed, and total RNA was extracted with the RNA extract kit (Cat# 7326820, Bio-Rad). Real-time PCR was performed with SYBR Green PCR Kit (Cat# 1725120, Bio-Rad) using 2 µl cDNA as a template with two primers. Thermocycler conditions were 36 cycles of denaturation at 95 ^{o}C for 15 seconds, annealing 56 ^{o}C for 10 seconds and extension step of 72 ^{o}C for 5 seconds. The ΔΔCT method was used to quantify all relative mRNA levels using small nuclear RNA U6 as the reference and internal control.

Immunohistochemistry staining (IHC)
Colon sections were de-paraffinized with xylene and ethanol and then boiled in a pressure cooker. After washing with Tris-Buffered-Saline (TBS) containing 0.025% Triton X-100, the sections were blocked with 10% goat serum and incubated with primary antibody in TBS containing 10% goat serum at 4 ° C overnight. The sections were washed with TBS and labeled with biotinylated secondary antibody, followed by avidin conjugated horseradish peroxidase provided by the Vectastain ABC kit (Cat# PK-6100, Vector Laboratories). DAB staining (Cat# PK-4100, Vector Laboratories) was performed, followed by Mayer's Hematoxylin for counter staining. The representative images/figures were selected based on their quality and accurate representation of similarity with the average value of each experimental group. ImageJ was used to analyze the images.

Fluorescence in situ hybridization (FISH)
The frozen colon sections were permeabilized with 0.02% Triton X-100 for 15 min. Then, 40 nM Cy5 or FITC-labeled DNA oligo probes were applied in a hybridization buffer (Ambion), and in situ hybridization was carried out for 3 hours at 52° C. This was followed by a series of washes with saline-sodium citrate (SSC) buffers. Subsequently, the slides were subjected to further immunofluorescence staining. DAPI was used to stain DNA. Images of the staining samples were performed using Nikon N-SIM S confocal laser scanning microscopy. The representative images/figures were selected based on their quality and accurate representation of similarity with the average value of each experimental group. ImageJ was used to analyze the images.

Immunofluorescence staining
For immunofluorescence staining, colon sections were de-paraffinized with xylene and ethanol, washed with PBS, and blocked with 10% goat serum for 30 min. the sections were incubated with primary antibody in TBS containing 10% goat serum at 4 ° C overnight. The slides were washed and incubated with Alexa Fluor 488, 555 or 647 second antibodies at room temperature for 2 h. DAPI was used to stain DNA. Images of the staining samples were performed using Nikon N-SIM S confocal laser scanning microscopy. The representative images/figures were selected based on their quality and accurate representation of similarity with the average value of each experimental group. ImageJ was used to analyze the images.

Enzyme-linked immunosorbent assay (ELISA)
To detect cytokine levels secreted by the colon, Multi-Analyte ELIS Array Kits for cytokines (MEN 004A, Qiagen) were employed. In brief, 50 μL assay buffer and 50 μL tested sample was added to each well of a 96-well ELISA high binding plate coated with indicated polyclonal cytokine antibodies and incubated at room temperature for 2 h. After washed, Detection Antibodies (monoclonal antibodies against above cytokines) were added and incubated for 1 h, followed by second antibody (HRP, 1:4000) for 2 h. The plates were washed and incubated with 100 μl of dilute Avidin-HRP for 30 min. After incubated with 100 μl the Development Solution for 15 min, 100 μl of Stop Solution was added to each well, and optical density of samples were read at 450 nm. Standard curves were drawn with standard antigens and OD values were then interpolated to determine sample protein concentrations.

Delivery plasmids, siRNAs or mixmer with nanoparticles
Plasmids, siRNAs or mixmer were conjugated with mPEG (Cat# PG1-TH-2k-1, Nanocs) and AUNP (Cat# CG-10-20, Cytodiagnostics) that formed complexes before injection. Synthesis of the delivered complexes (plasmid/siRNAs/mixmer-PEG-Au NP) was performed as previously described. The complexes (plasmid, 50 μg/mouse; siRNAs/mixmer, 5 μg/mouse) were injected intraperitoneally two days before DSS or TNBS treatment, and injected every two days until euthanized.

Isolation of primary mouse colon epithelial cell
Primary mouse colonic epithelial cells (mCECs) were isolated as previously described [2]. Briefly, Mouse colonic mucosa was dissected, cut into 3 pieces of 2 cm long and washed with Hank's Balanced Salt Solution (HBSS) containing penicillin and streptomycin. The washed tissues were suspended in 40 ml HBSS, inverted vigorously 15 times, and allowed to settle for 2 min. The supernatant was removed and the settled contents were washed an additional 5 times. The settled contents were minced and suspended in 40 ml of the HBSS. The suspension was passed over a 1000 μm^2 mesh filter. Remaining tissue was digested in 40 ml of a Dulbecco's Modified Eagle Medium with 4.5 g/l glucose and L-glutamine, without sodium pyruvate (DMEM) containing 75 U/ml collagenase type XI, 20 μg /l dispase neutral protease II, 0.5 mM DTT, and 0.5 % FBS. The digestion mixture was shaken at 180 rpm 37 ° C for 2 h, and passed over a 1000 μm^2 filter. The tissue fragments atop the filter were washed with 25 ml DMEM growth media (DMEM, 8.5 g/l sodium pyruvate, 2.5 % FBS, 0.25 U/ml insulin, 100 U penicillin, 100 μg/ml streptomycin, 5 μg/ml transferrin, and 10 ng/ml epidermal growth factor) containing 2% D-sorbitol (S-DMEM). The effluent containing proliferative crypt structures was centrifuged at 200×g for 5 min at 4 ° C. The remaining pellet was suspended in S-DMEM. This process was repeated four times. After washing, the crypts were suspended in DMEM growth media, plated at 2 % gelatin coated culture dish and incubated at 37 ° C.

Histological damage score
Hematoxylin and eosin stained colon sections were used for histological assessment of colitis. Two slides for each experimental group were scored by three observers blinded to the treatment groups, using previously described criteria [3]: 0, no signs of inflammation; 1, very low level of leukocyte infiltration; 2, low level of leukocyte infiltration; 3, high level of leukocyte infiltration, high vascular density, thickening of the colon wall; 4, transmural infiltration, loss of goblet cells, high vascular density, thickening of the colon wall.

Epithelial permeability assay

On euthanizing day after DSS or TNBS treatment, mice were orally gavaged with FITC-dextran (600 mg/kg body). Four hours following gavage, mice were anesthetized, the blood was collected via cardiac puncture, and allowed to be kept in room temperature for 1h. After centrifuged at 3000 RPM for 10 min at 4°C, the collected supernatant was read on a spectrophotometer at 485/535 nm.

Colonic mucosa culture

Freshly obtained colonic mucosa (2 cm in length) was washed with HBSS containing penicillin and streptomycin, and cultured in 1 mL DMEM medium containing 10% at 37° C with 5% CO2 for 24 h. The cultured medium was then harvested, followed by ELISA to detect cytokine levels secreted by colonic mucosa.

Disease activity index

The mice were checked each day for morbidity, and colitis severity was monitored using the disease activity index (DAI), which includes evaluation of weight loss, stool consistency, and presence of fecal blood[4]. DAI was calculated for each mouse daily based on body weight loss, bleeding and stool consistency. A score of 1–4 was given for each parameter, with a maximum DAI score of 12. Score 0: no weight loss, normal stool, no blood; score 1: 1–3% weight loss; score 2: 3–6% weight loss, loose stool, blood visible in stool; score 3: 6–9% weight loss; score 4: 49% weight loss, diarrhea, gross bleeding. Gross bleeding was defined as fresh blood on fur around the anus or with extensive blood in the stool. Loose stool was defined as the formation of a stool that readily becomes paste, and diarrhea was defined as no stool formation.

References

1. Biesiadecki, B.J. & Jin, J.P. A high-throughput solid-phase microplate protein-binding assay to investigate interactions between myofilament proteins. *J Biomed Biotechnol* **2011**, 421701 (2011).
2. Thomson, P.J., Soames, J.V., Booth, C. & O'Shea, J.A. Epithelial cell proliferative activity and oral cancer progression. *Cell Prolif* **35 Suppl 1**, 110-120 (2002).
3. Wang, H. *et al.* Pro-inflammatory miR-223 mediates the cross-talk between the IL23 pathway and the intestinal barrier in inflammatory bowel disease. *Genome biology* **17**, 58 (2016).
4. Lin, W. *et al.* Raf kinase inhibitor protein mediates intestinal epithelial cell apoptosis and promotes IBDs in humans and mice. *Gut* **66**, 597-610 (2017).

2. Supplementary Tables

Supplementary Table S1. Primers used in the study

Primer Name	Sequence	Source / Repository
cirNlg genotying-1F	5’ ctcgagacacttagccgtgttctt	Eurofins Genomics LLC
cirNlg genotying-1R	5’ tataaatcaaatccagttagc	Eurofins Genomics LLC
cirNlg genotying-2F	5’ggatccctacctcttctggtggcc	Eurofins Genomics LLC
cirNlg genotying-2R	5’ actaataacttggatgtggtttca	Eurofins Genomics LLC
humu.cirNlgn-precu	5’ ggatcccctggcctcccggagctg	Eurofins Genomics LLC
humu.circNlgn-precu	5’ gcagcatcttcaatcatcactatt	Eurofins Genomics LLC
humu.cirNlgn-R1	5’ actaataacttggatgtggtttca	Eurofins Genomics LLC
humu.cirNlgn-F1	5’ tcccgactgaggatggatatagac	Eurofins Genomics LLC
humu.cirNlgn-R2	5’ gcagcatcttcaatcatcactatt	Eurofins Genomics LLC
humu.cirNlgn-F2	5’ tcccgactgaggatggatatagac	Eurofins Genomics LLC
humu.cirNlgn-R3	5’ tataaatcaaatccagttagc	Eurofins Genomics LLC
humu.cirNlgn-F3	5’ gtgcaagaccagagcgaagactg	Eurofins Genomics LLC
hu.Nlgn-mRNA-F1	5’ ctgtgaagcttgaggccactcaag	Eurofins Genomics LLC
hu.Nlgn-pre-mRNA-F	5’ actaataacttggatgtggtttca	Eurofins Genomics LLC
hu.Nlgn-pre-mRNA-R	5’ ccatgcatactaccagtacacatg	Eurofins Genomics LLC
mu.Nlgn-mRNA-F	5’ gactgtattgcccttggaatgagg	Eurofins Genomics LLC
mu.Nlgn-mRNA-R	5’ catcactattcatcaggacatctc	Eurofins Genomics LLC
mu.Nlgn-pre-mRNA-F	5’ catctggttatcacttatgtctgt	Eurofins Genomics LLC
mu.Nlgn-pre-mRNA-R	5’ catcactattcatcaggacatctc	Eurofins Genomics LLC
mu.IL-1β-F	5’ gaaatgccaccttttgacagtg	Eurofins Genomics LLC
mu.IL-1β-R	5’ tggatgctctcatcaggacag	Eurofins Genomics LLC
mu-IL-2-F	5’ tgagcaggatggagaattacagg	Eurofins Genomics LLC
mu-IL-2-R	5’ gtccaagttcatcttctaggcac	Eurofins Genomics LLC
mu-IL-6-F	5’ ttccatccagttgccttcttg	Eurofins Genomics LLC
mu-IL-6-R	5’ gggagtggtatcctctgtgaagtc	Eurofins Genomics LLC
mu.TNFα-F	5’ tggatctcaa agacaaccaa ctag	Eurofins Genomics LLC
mu.TNFα-R	5’ ggcaggggctcttgacggcagaga	Eurofins Genomics LLC
mu.G-CSF-F	5’ atggctcaactttctgcccag	Eurofins Genomics LLC
mu.G-CSF-R	5’ ctgacagtgaccaggggaac	Eurofins Genomics LLC

Supplementary Table S2. siRNAs used in the study

siRNA Name	Sequence	Source / Repository
hu.cirNlgn1	5’ ccgacugaggauggauauauu	Gene Universal
hu.cirNlgn2	5’ cccgacugaggauggauauuu	Gene Universal
mu.cirNlg1	5’ cccaacugaagauggauauuu	Gene Universal
mu.cirNlg2	5’ ccaacugaagauggauauauu	Gene Universal
hu.mu.LmnB-1	5’ gcagccuggagacggagaauu	Gene Universal
hu.mu.LmnB-2	5’ ggcugggagaugaucagaauu	Gene Universal

3. Supplementary Figures

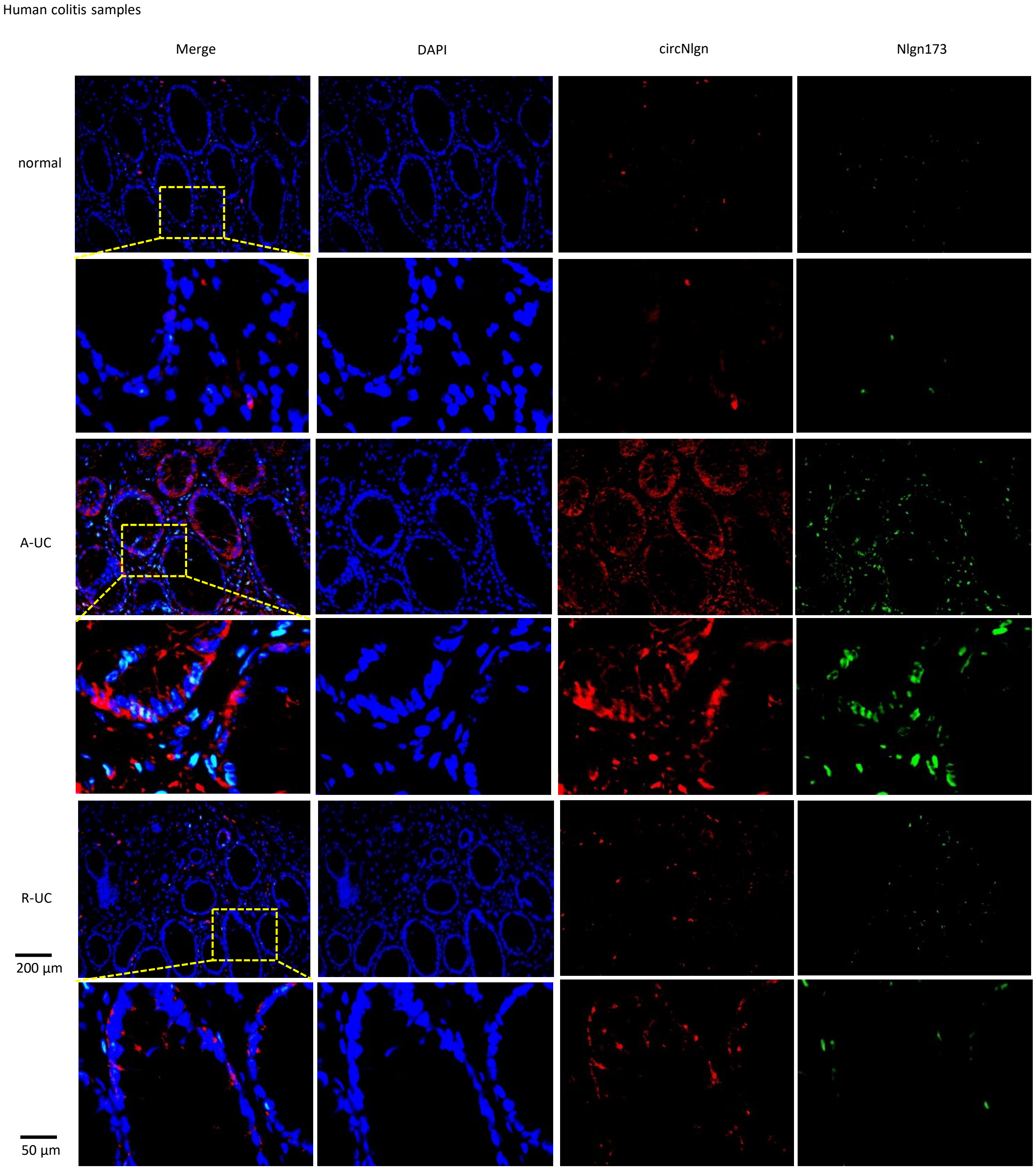

Fig S1. Increased Nlgn173 Protein Expression in Human Colon Mucus (A-UC). In situ hybridization immunofluorescence staining showed circNlgn and Nlgn173 protein levels in human colon mucus with colitis, which were high in acute ulcerative colitis (A-UC).

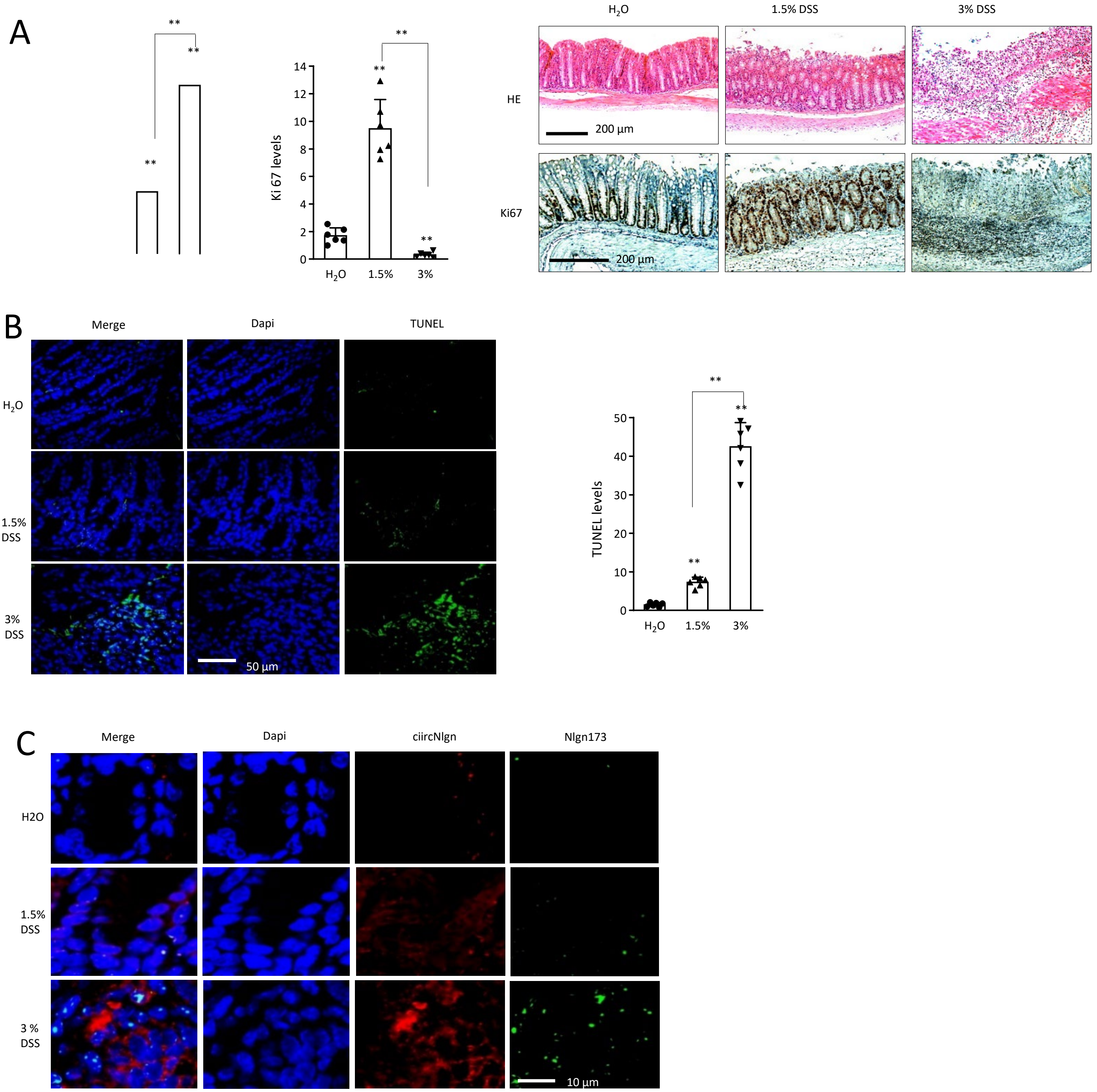

Fig S2, Assessment of Colon Damage, Cellular Proliferation, and Apoptosis in Response to DSS Treatment

A. Left, Histological damage score analysis of HE staining revealed that DSS treatment induced damage to colon mucosa, and the damage was dose-dependent. ***p<0.01* versus H_2O group (*n=6*).

Middle, ImageJ analysis indicated that mildly damaged colon mucosa exhibited increased Ki67 (1.5 % DSS group), while severely damaged colon mucosa showed significantly decreased Ki67 expression (3 % DSS group). ***p<0.01* versus H2O group (*n=6*).

Right, upper, Representative images of HE staining of treated mouse colon sections.

Right, lower, Typical Ki67 staining images of treated mouse colon section.

B. Left, C57BL/6J mice were exposed to 1.5 % or 3 % DSS for 7 days, followed by a return to tap water for 3 days. The colon tissue sections were subjected to TUNEL staining, revealing increased apoptosis in the DSS-treated tissues.

Right, ImageJ analysis showed that DSS treatment significantly elevated TUNEL-positive cells in colon mucosa, especially in the 3% DSS-treated mice. ***p<0.01* versus H2O group (*n=6*).

C. Representative images of in situ hybridization staining demonstrated the expression of circNlgn (red) and Nlgn173 (green) in the mouse colon mucosa, with DAPI used to stain nuclei (blue).

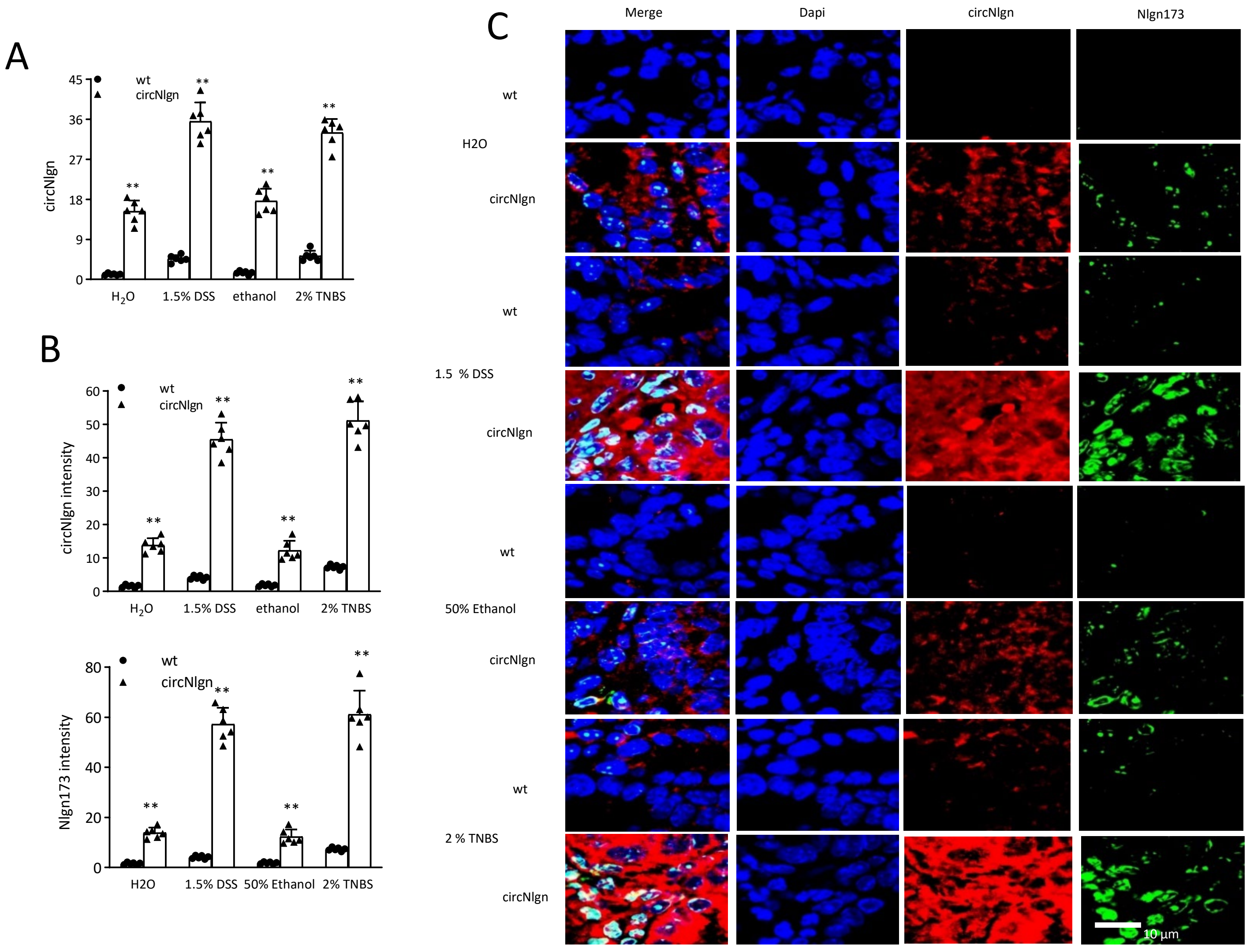

Fig S3. circNlgn enhanced colitis development.

A. RT-PCR revealed significantly higher levels of circNlgn expression in circNlgn(+) colon mucosa compared to wild-type (wt) mice. $^{**}p<0.01$ versus wt ($n=6$).

B. Left, ImageJ analysis of in situ hybridization staining showed that circNlgn(+) colon mucosa expressed much higher levels of circNlgn than wt. $^{**}p<0.01$ versus wt ($n=6$).

Right, ImageJ analysis of immunofluorescence staining showed substantially higher Nlgn173 protein levels in circNlgn(+) colon mucosa compared to wt. $^{**}p<0.01$ versus wt ($n=6$).

C. Typical images showing the expression of circNlgn (red) and Nlgn173 (green) in mouse colon mucosa from wt or circNlgn(+) mice, with or without DSS or TNBS treatment.

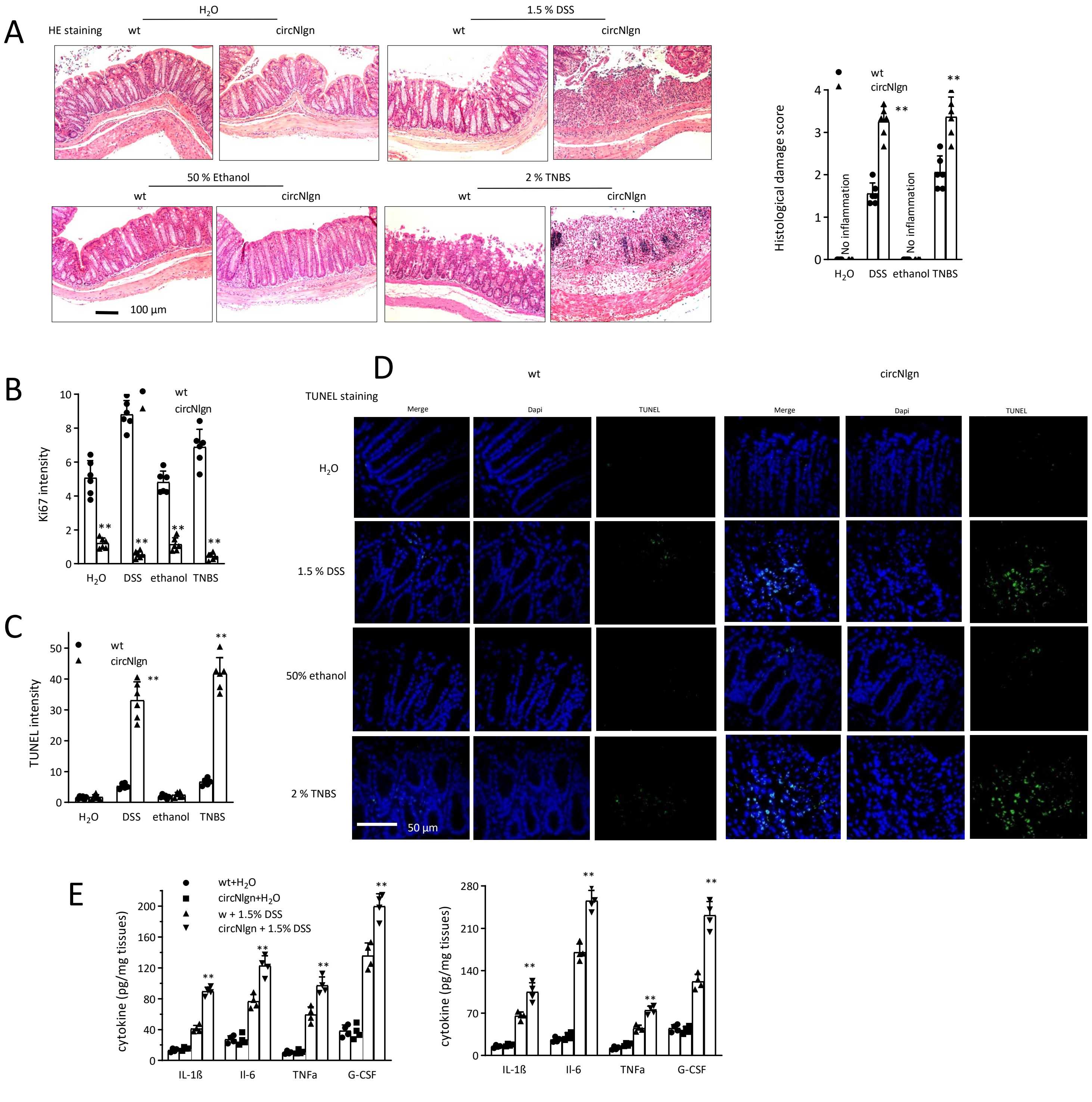

Fig S4. circNlgn expression enhanced apoptosis.

A. Left, Typical images of HE staining of mouse colon sections of above mice. Right, The graph displayed that colon sections from circNlgn(+) mice exhibited higher histological damage scores than wt mice after DSS or TNBS treatment.

B. ImageJ analysis of immunofluorescence staining revealed that circNlgn(+) colon mucosa expressed lower Ki67 levels compared to wt after DSS or TNBS treatment.

C. ImageJ analysis of immunofluorescence staining indicated that circNlgn(+) colon mucosa had a higher intensity of TUNEL staining following DSS or TNBS treatment.

D. Typical images of TUNEL staining of mouse colon mucosa sections.

E. Colon mucosa was cultured in DMEM for 24 hours, and the supernatant was subjected to ELISA assays, demonstrating that circNlgn(+) mouse mucosa secreted significantly higher levels of IL-1β, IL-6, TNFα, and G-CSF after DSS (left) or TNBS treatment (right).

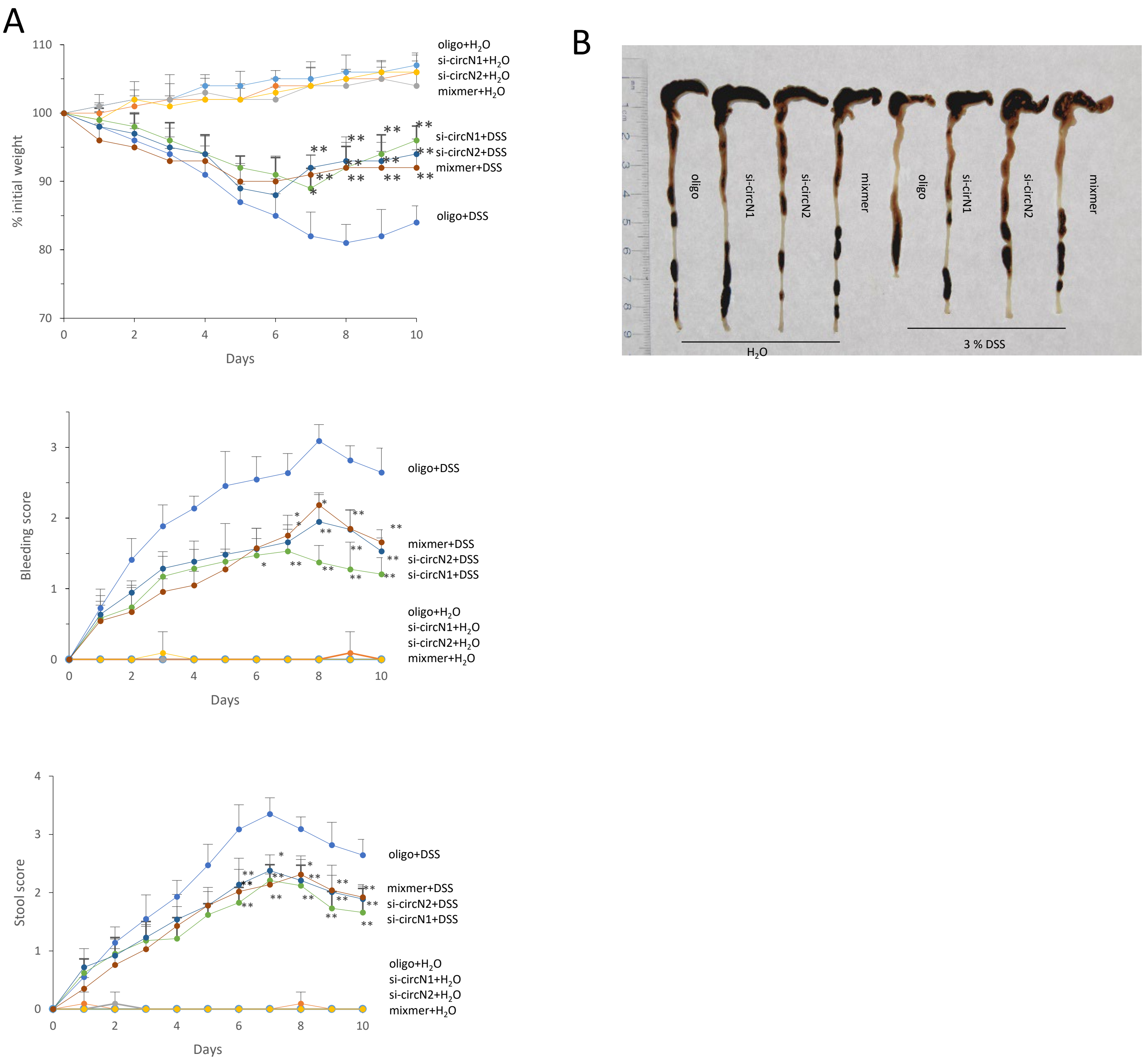

Fig S5. Silencing circNlgn or blocking circNlgn translation decreased colitis symptoms.

A. Upper, Mice were administrated with 3 % DSS, and the body weight was assessed daily until euthanized. Silencing circNlgn with siRNAs or blocking circNlgn translation with a mixmer mitigated DSS enhanced body weight losing. ***p<0.05,* ***p<0.01* versus oligo (*n=10*).

Middle, The graph showed that silencing circNlgn with siRNAs or blocked circNlgn translation with a mixmer mitigated DSS induced bleeding score increase. ***p<0.05,* ***p<0.01* versus oligo (*n=10*).

Lower, The graph showed that silencing circNlgn with siRNAs or blocked circNlgn translation with a mixmer mitigated DSS induced stool score increase. ***p<0.05,* ***p<0.01* versus oligo (*n=10*).

B. Silencing circNlgn with siRNAs or blocked circNlgn translation with a mixmer prevented the shortened colon length induced by DSS treatment. A typical image showed the colon length of mice in the various groups with or without DSS treatment.

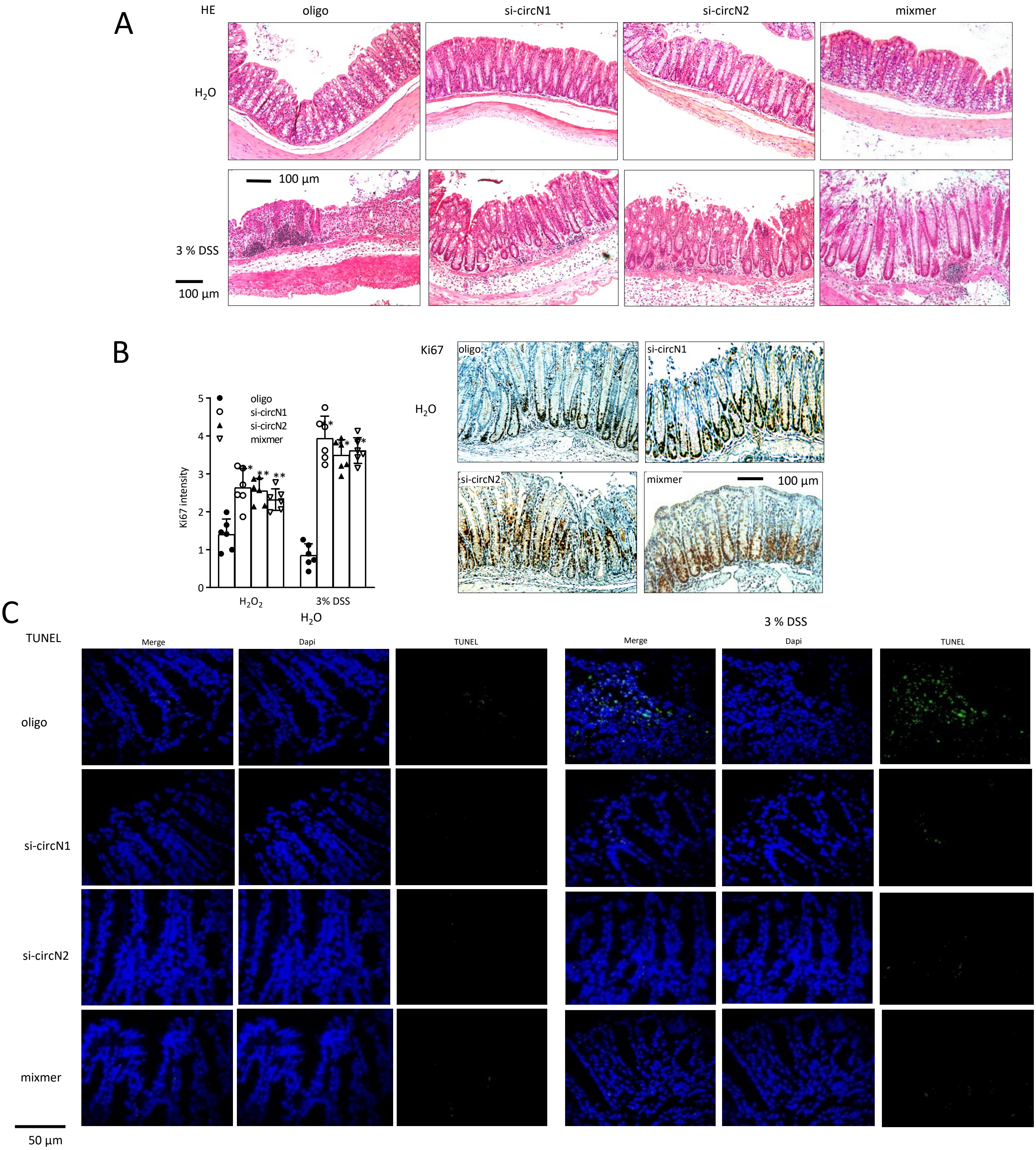

Fig S6. Silencing circNlgn or blocking circNlgn translation mitigated colitis.

A. The colon sections from mice that received circNlgn siRNAs or mixmers exhibited lower histological damage scores than the control mice after DSS treatment. Typical images of HE staining of mouse colon sections.

B. Left, ImageJ analysis of immunofluorescence staining revealed that mice receiving circNlgn siRNAs or mixmers had higher Ki67 levels in the colon mucosa than control mice after DSS treatment. ***$p<0.01$* versus oligo (*$n=6$*). Right, Typical images of Ki67 IHC staining of untreated mouse colon mucosa.

C. TUNEL staining showed that mice receiving circNlgn siRNAs or mixmers displayed lower TUNEL intensity than control mice after DSS treatment. Typical images of TUNEL staining of mouse colon mucosa.

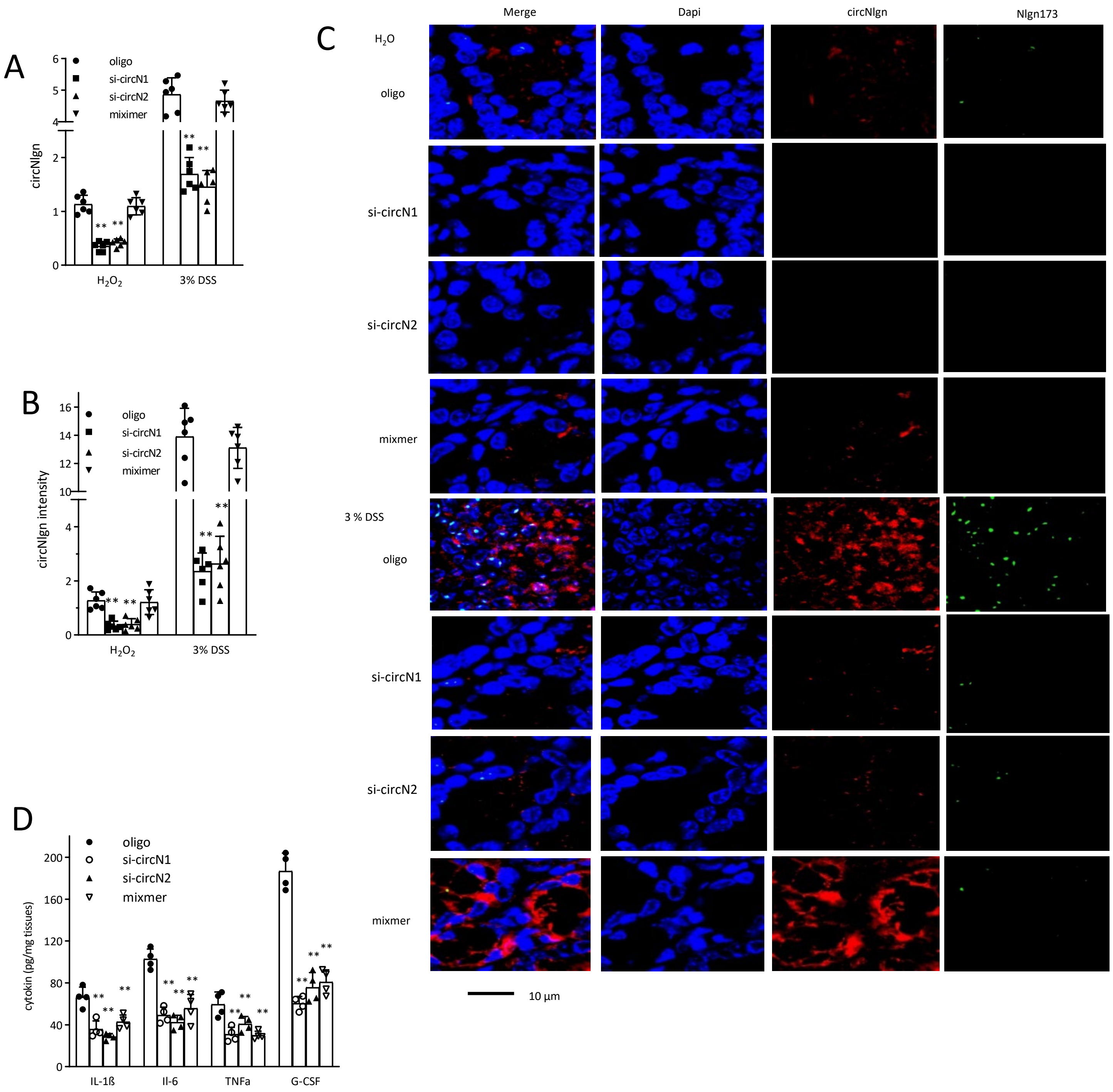

Fig S7. Silencing circNlgn or blocking circNlgn translation decreased cytokine expression.

A. RT-PCR showed that colonic mucosa from mice receiving circNlgn siRNAs expressed lower levels of circNlgn than those receiving oligos. **$p<0.01$ versus oligo ($n=6$).

B. Left, ImageJ analysis of in situ hybridization staining showed that colonic mucosa from mice receiving circNlgn siRNAs expressed lower levels of circNlgn than those receiving oligos. **$p<0.01$ versus oligo ($n=6$).

C. ImageJ analysis of immunofluorescence staining showed that colonic mucosa from mice receiving circNlgn siRNAs or mixmers expressed lower levels of Nlgn173 protein than those receiving oligos, both with or without DSS treatment. **$p<0.01$ versus oligo ($n=6$).

D. Colonic mucosa was cultured in DMEM for 24 h, and the supernatant were subjected to ELISA assays, showing that mice receiving circNlgn siRNAs or mixmers secreted significantly lower levels of IL-1β, IL-6, TNFα, and G-CSF after DSS treatment. **$p<0.01$ versus oligo ($n=4$).

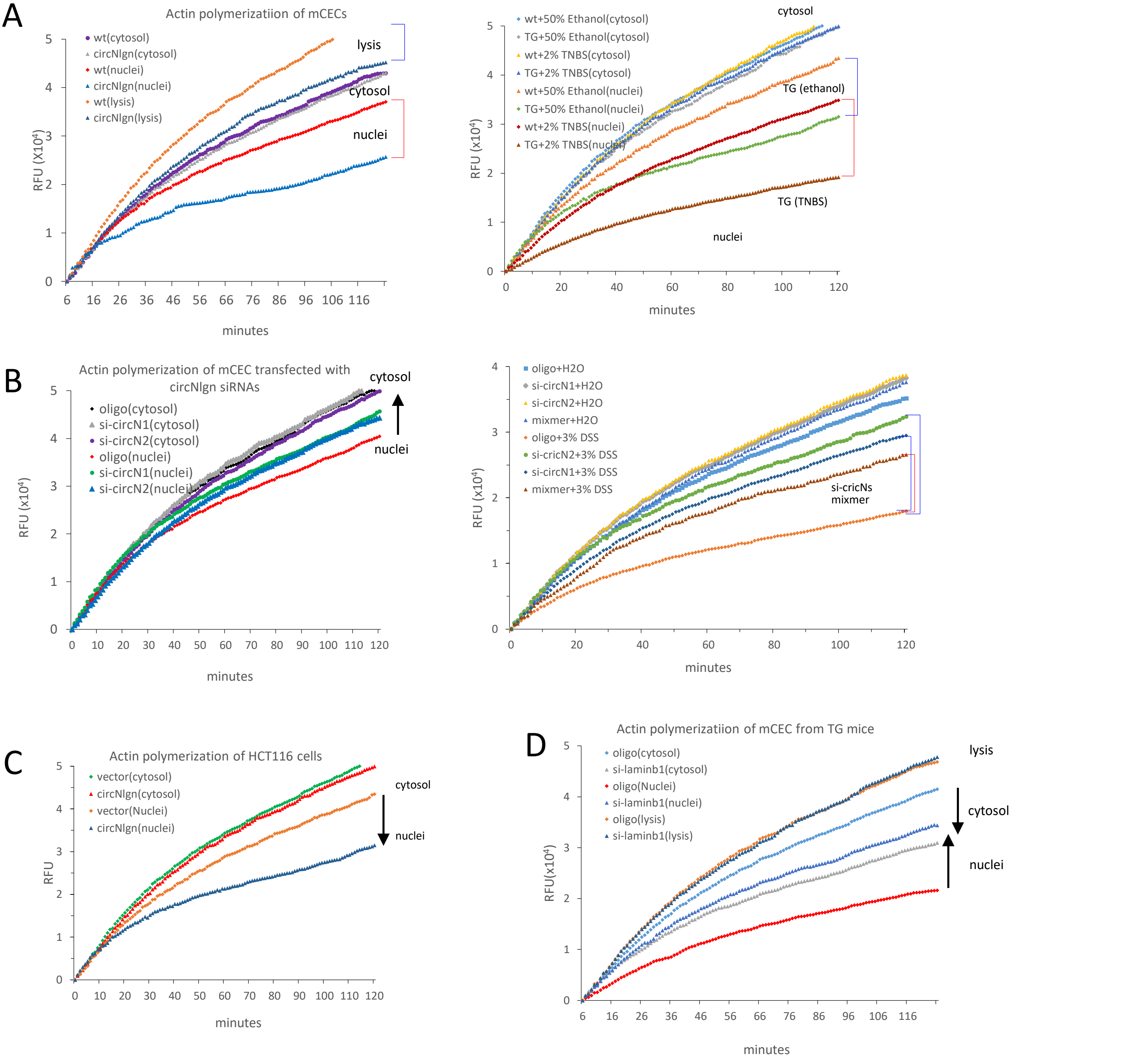

Fig S8. Nlgn173 repressed nuclear actin polymerization.

A. Left, mCECs isolated from wt and circNlgn(+) mice treated with 1.5% DSS were collected and processed to actin polymerization assay. Total lysate and nuclear lysate of circNlgn(+) mucosa suppressed actin polymerization.

Right, Colonic mucosa from wt and circNlgn(+) mice treated with 2% TNBS was processed to actin polymerization assay. Lysate of nuclear extract from circNlgn(+) mucosa showed suppression of actin polymerization.

B. Left, Colonic mucosa from circNlgn siRNA- and mixmer-delivered mice treated with or without 1.5% DSS was processed to actin polymerization assay. The nuclear extract of circNlgn siRNAs and mixmer-delivered mouse mucosa displayed increased actin polymerization compared with oligo control. Right, mCECs isolated from circNlgn siRNA- and mixmer-delivered mice treated with or without 1.5% DSS were processed to actin polymerization assay. The nuclear extract of mCECs treated with the siRNAs and mixmer displayed increased actin polymerization.

C. HCT116 cells transfected with circNlgn were subjected to subcellular fractionation and actin polymerization assay. The nuclear extracts from the circNlgn-transfected cells decreased actin polymerization.

D. mCECs isolated from wt and circNlgn(+) mice, and transfected with LaminB1 siRNAs were subjected to subcellular fractionation. The nuclear extract from the cells transfected with the siRNAs showed increased actin polymerization, but the cytosol had opposite effect.

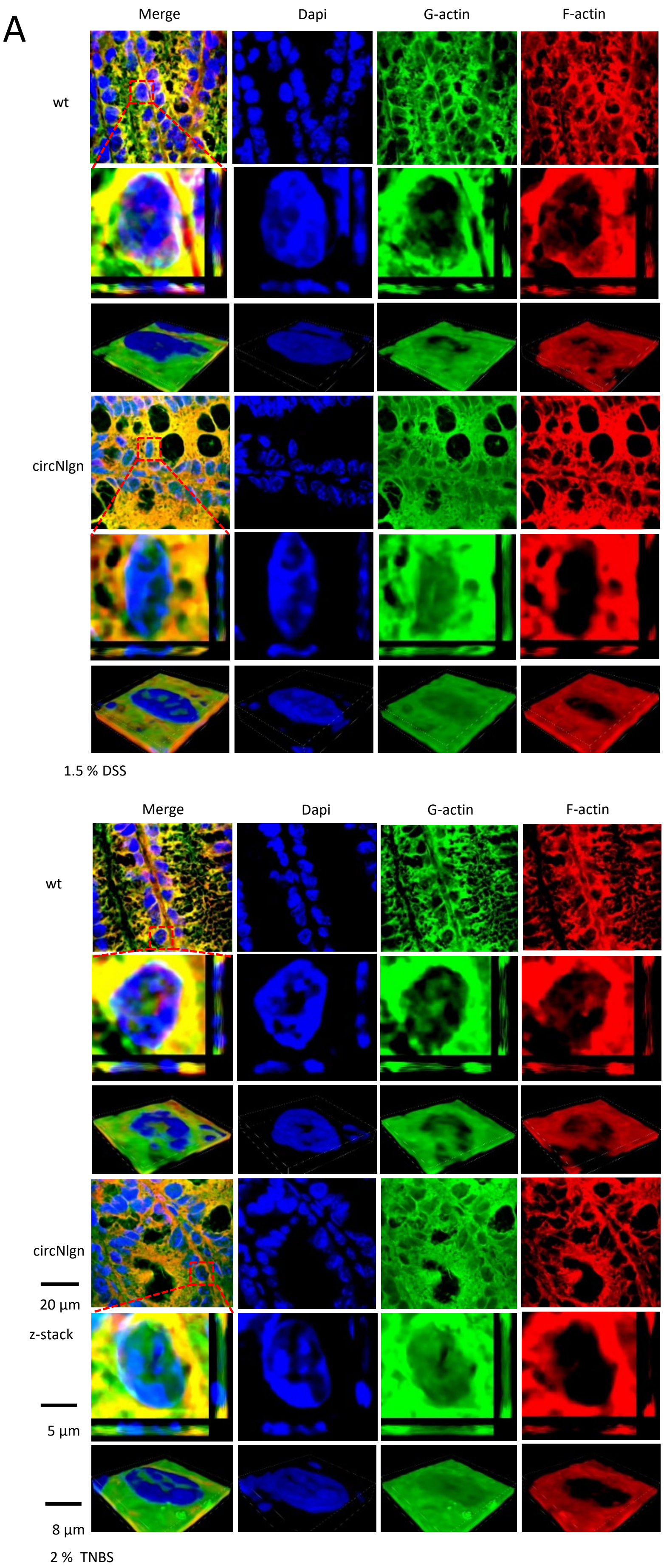

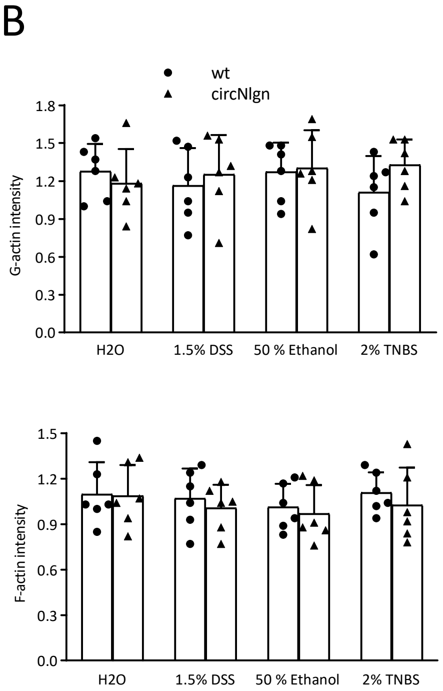

Fig S9. Levels of nuclear G-actin and F-actin affected by circNlgn expression.

A. Typical z-stack images (xy, xz and yz projection and orthogonal view) showed nuclear F-actin (Phalloidin staining, red) and G-actin (Deoxyribonuclease I staining, green) of circNlgn transgenic mouse colon mucus after 1.5 % DSS (upper) and 2 % TNBS (lower) treatment.

B. Image J analysis displayed the intensity of G-actin (upper) and F-actin (lower) in wt and circNlgn(+) mouse mucosa treated with 1.5% DSS and 2% TNBS (n=6).

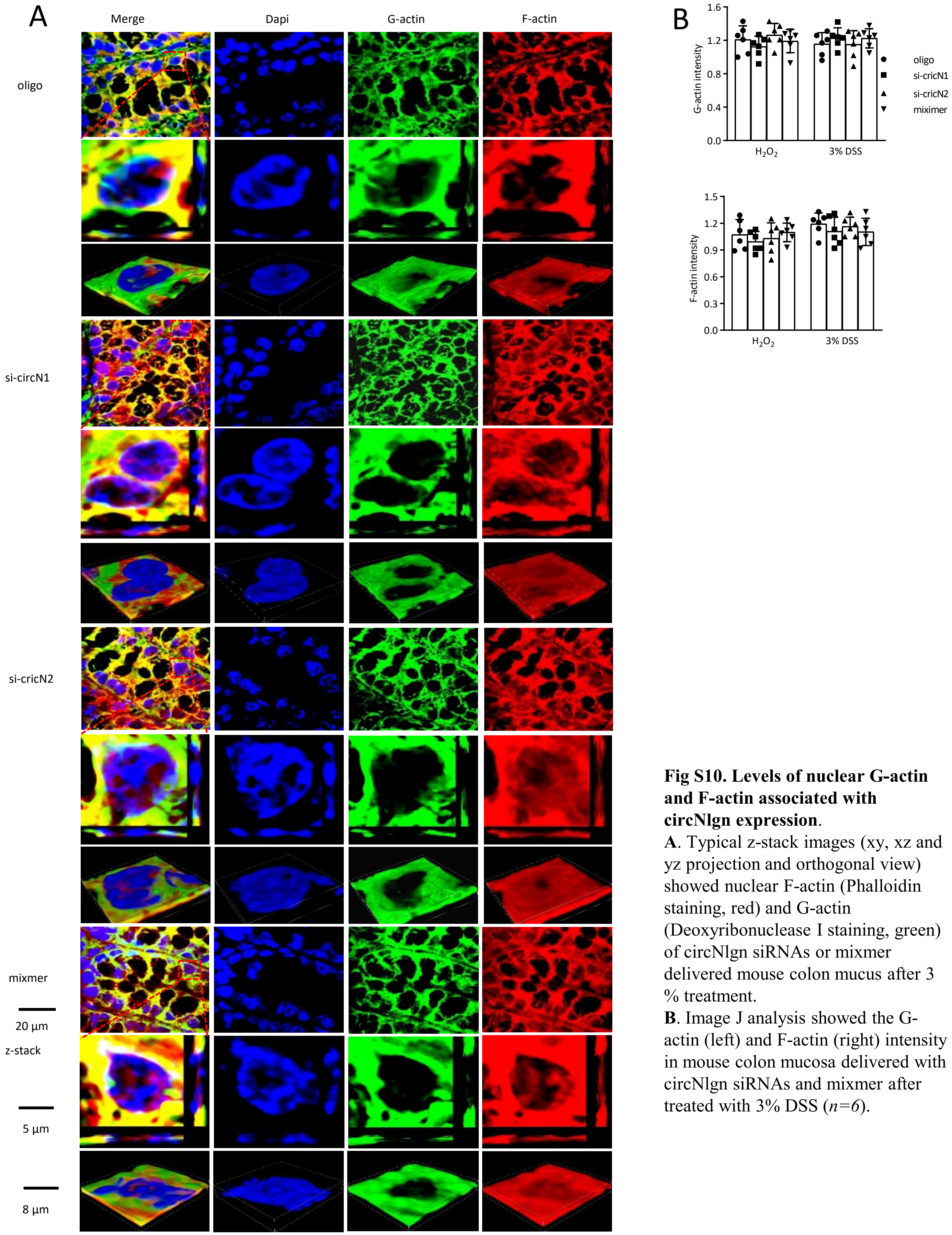

Fig S10. Levels of nuclear G-actin and F-actin associated with circNlgn expression.

A. Typical z-stack images (xy, xz and yz projection and orthogonal view) showed nuclear F-actin (Phalloidin staining, red) and G-actin (Deoxyribonuclease I staining, green) of circNlgn siRNAs or mixmer delivered mouse colon mucus after 3 % treatment.

B. Image J analysis showed the G-actin (left) and F-actin (right) intensity in mouse colon mucosa delivered with circNlgn siRNAs and mixmer after treated with 3% DSS (*n=6*).

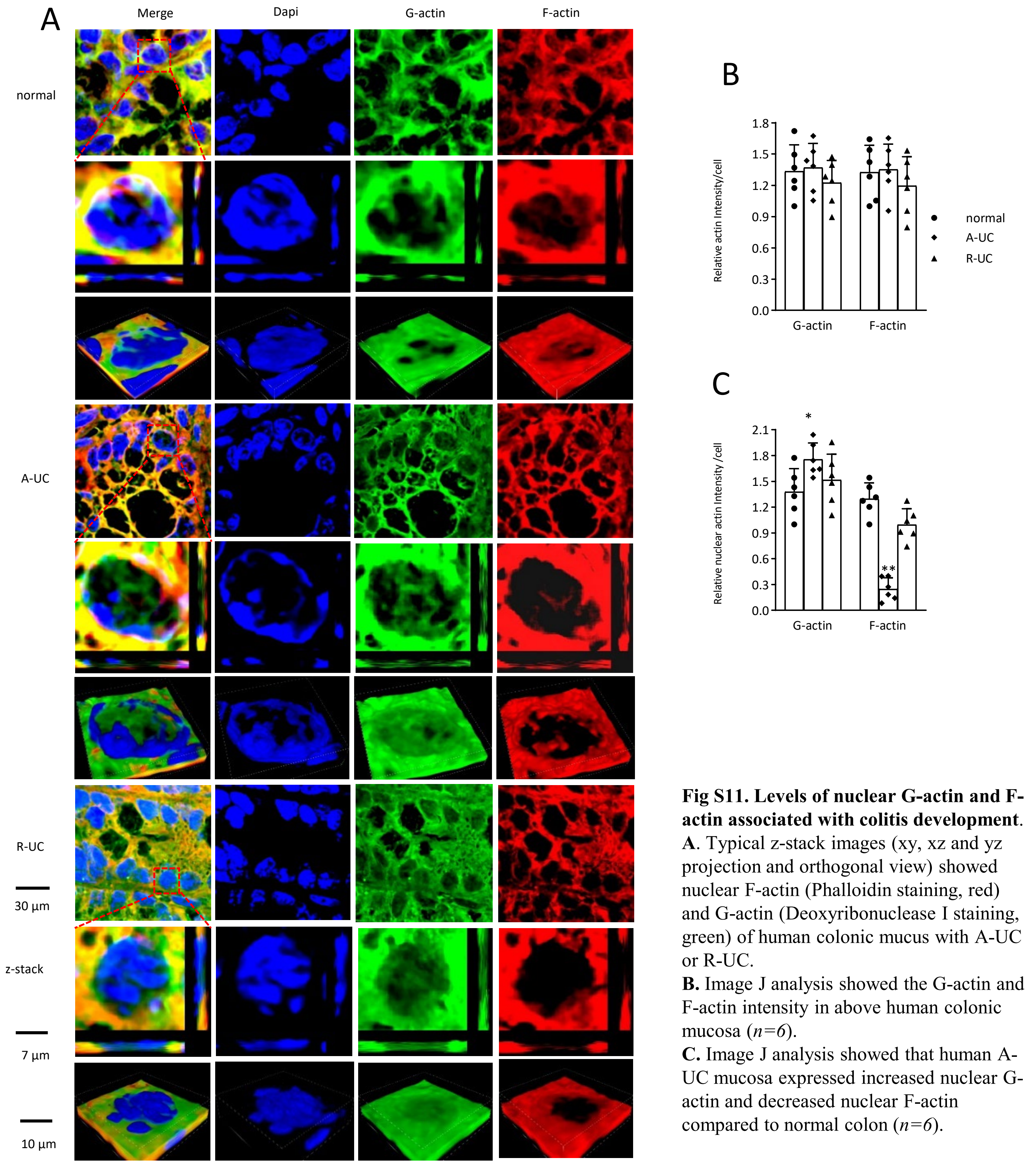

Fig S11. Levels of nuclear G-actin and F-actin associated with colitis development.
A. Typical z-stack images (xy, xz and yz projection and orthogonal view) showed nuclear F-actin (Phalloidin staining, red) and G-actin (Deoxyribonuclease I staining, green) of human colonic mucus with A-UC or R-UC.
B. Image J analysis showed the G-actin and F-actin intensity in above human colonic mucosa (*n*=*6*).
C. Image J analysis showed that human A-UC mucosa expressed increased nuclear G-actin and decreased nuclear F-actin compared to normal colon (*n*=*6*).

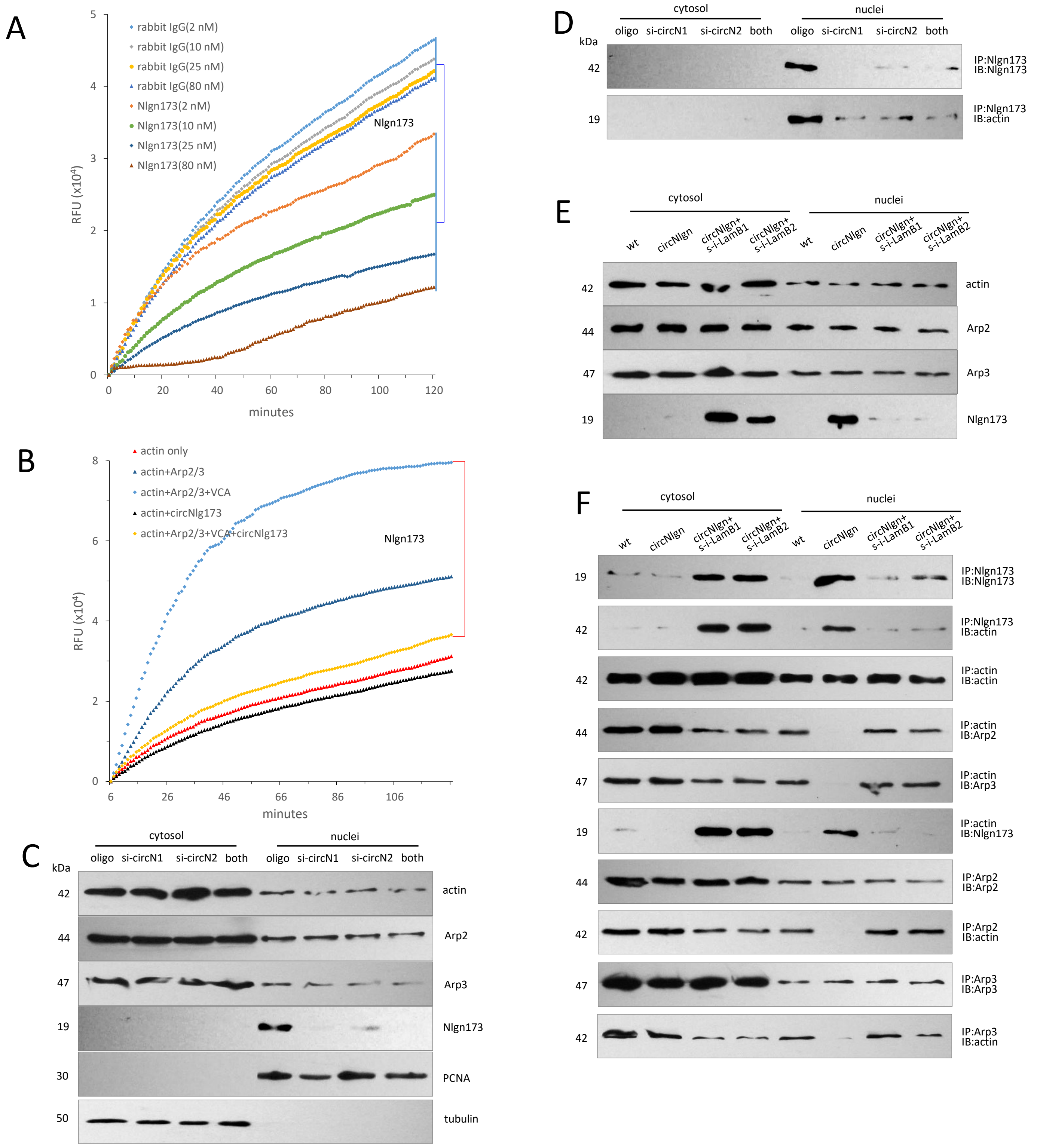

Fig S12. Effect of Nlgn173 on Arp2/3-mediated nuclear actin polymerization.

A. The purified Nlgn173 repressed actin polymerization in a dose-dependent manner.

B. The purified Nlgn173 repressed Arp2/3 dependent actin polymerization.

C. mCECs were transfected with circNlgn siRNAs and cultured with 100 μM NE for 24 h. The cells were subjected to subcellular fractionation and Western blot. Nlgn173 was mainly detected in nuclei.

D. Immunoprecipitation with an antibody against Nlgn173 precipitated Nlgn173 that pulled down actin in the nuclei.

E. mCECs from wt or circNlgn(+) mice were transfected with LaminB1 siRNAs and subjected to subcellular fractionation and Western blot. Silencing LaminB1 blocked Nlgn173 translocation from cytosol to the nucleus.

F. Immunoprecipitation with antibody against Nlgn173 co-precipitated actin in the nucleus of circNlgn(+) mCESs and actin in the cytosol of LaminB1 siRNA-transfected circNlgn(+) mCESs. Actin precipitation pulled down Arp2/3 and Nlgn173. Arp2/3 precipitation pulled down actin. Silencing LaminB1 decreased Nlgn173 binding to actin in nuclei but increased its binding to actin in the cytosol. Silencing LaminB1 increased Arp2/3 binding to actin in nuclei but decreased its binding to actin in the cytosol.

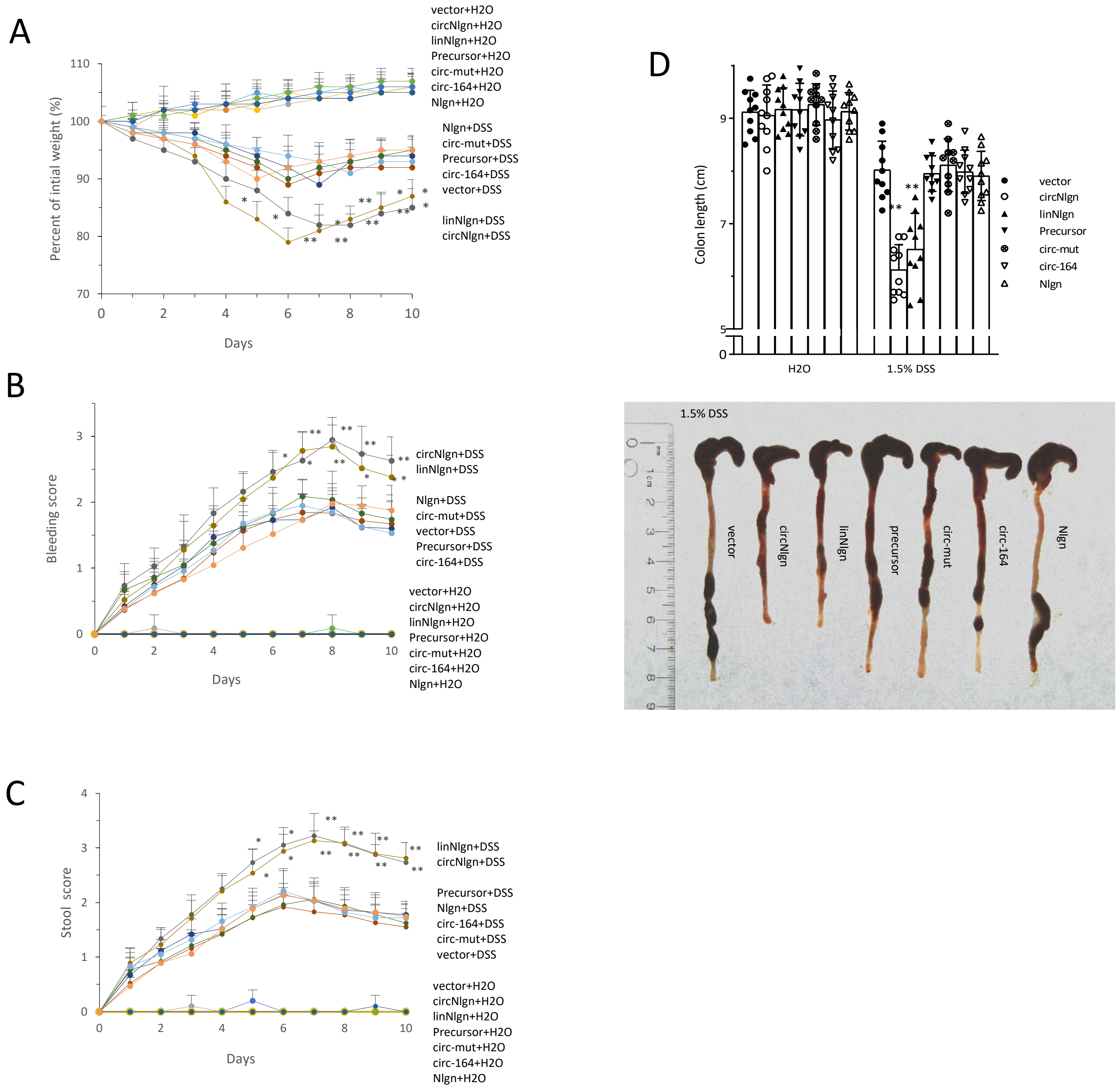

Fig S13. Colitis symptoms induced by the circNlgn translated protein.

A. Mice delivered with the above plasmids were administered 1.5% DSS, and body weight was assessed daily until euthanization. Delivery of circNlgn or linNlgn enhanced body weight loss induced by DSS treatment. ***p<0.05,* ***p<0.01* versus vector (*n=10*).

B. The graph showed that delivery of circNlgn or linNlgn increased mouse bleeding score after DSS treatment. ***p<0.05,* ***p<0.01* versus vector (*n=10*).

C. The graph showed that delivery of circNlgn or linNlgn increased the mouse stool score after DSS treatment. ***p<0.05,* ***p<0.01* versus vector (*n=10*).

D. Upper, the graph showed that mice delivered with circNlgn or linNlgn had shorter colon than control mice after DSS treatment. ***p<0.01* versus vector (*n=10*).

Lower, A representative image shows the colon length of the above group of mice with or without DSS treatment.

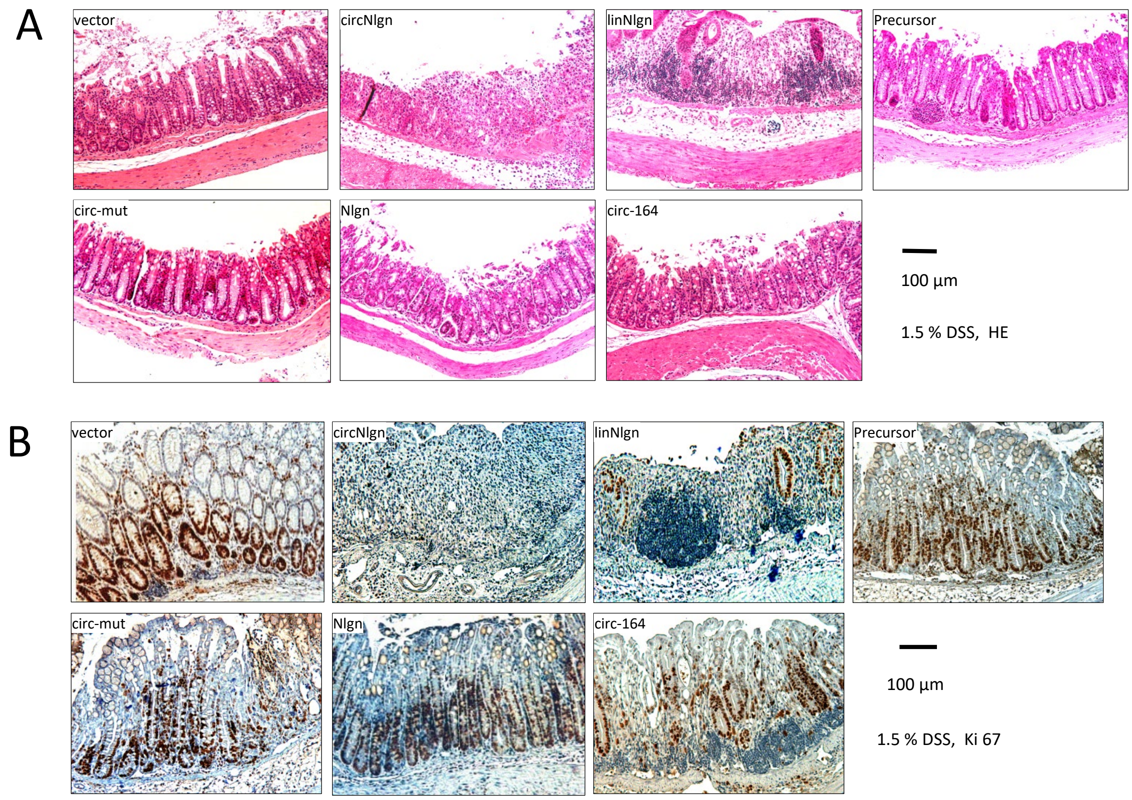

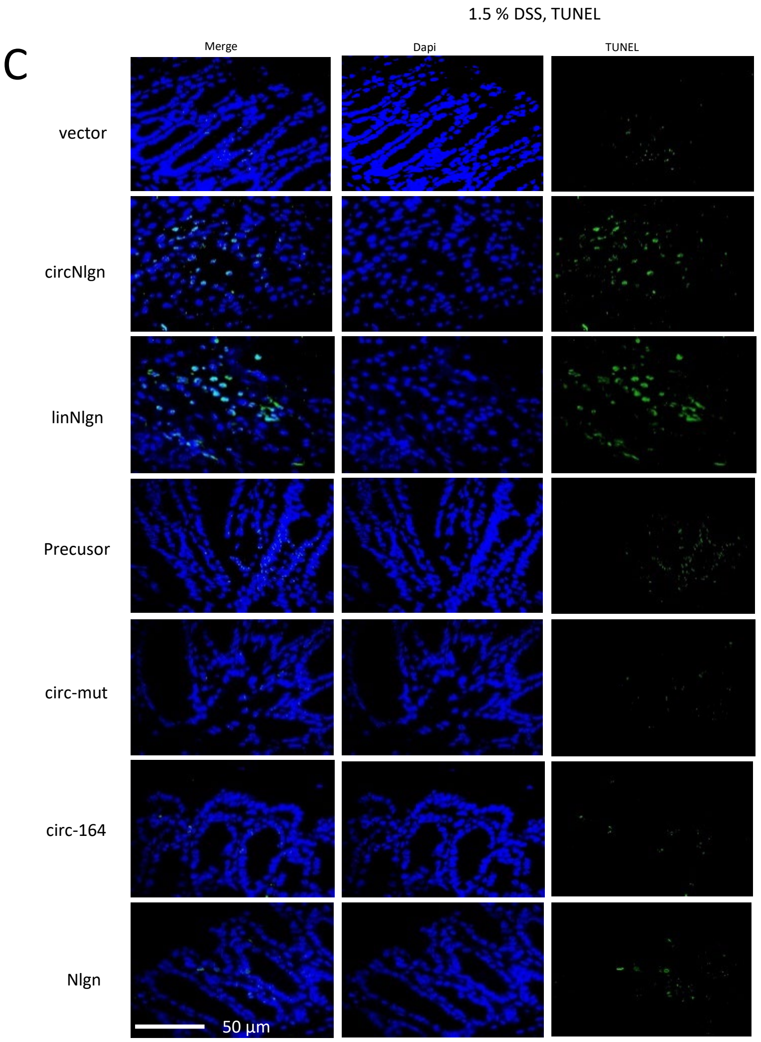

Fig S14. Colitis tissues affected by the circNlgn translated protein.

A. The graph showed that circNlgn or linNlgn delivered mouse colon sections displayed higher histological damage score than control mice after DSS treatment. Typical images of HE staining of mouse colon sections of above treated mice.

B. Typical images of Ki67 IHC staining of above treated mouse colon mucosa.

C. Typical images of TUNEL staining of above treated mouse colon mucosa.

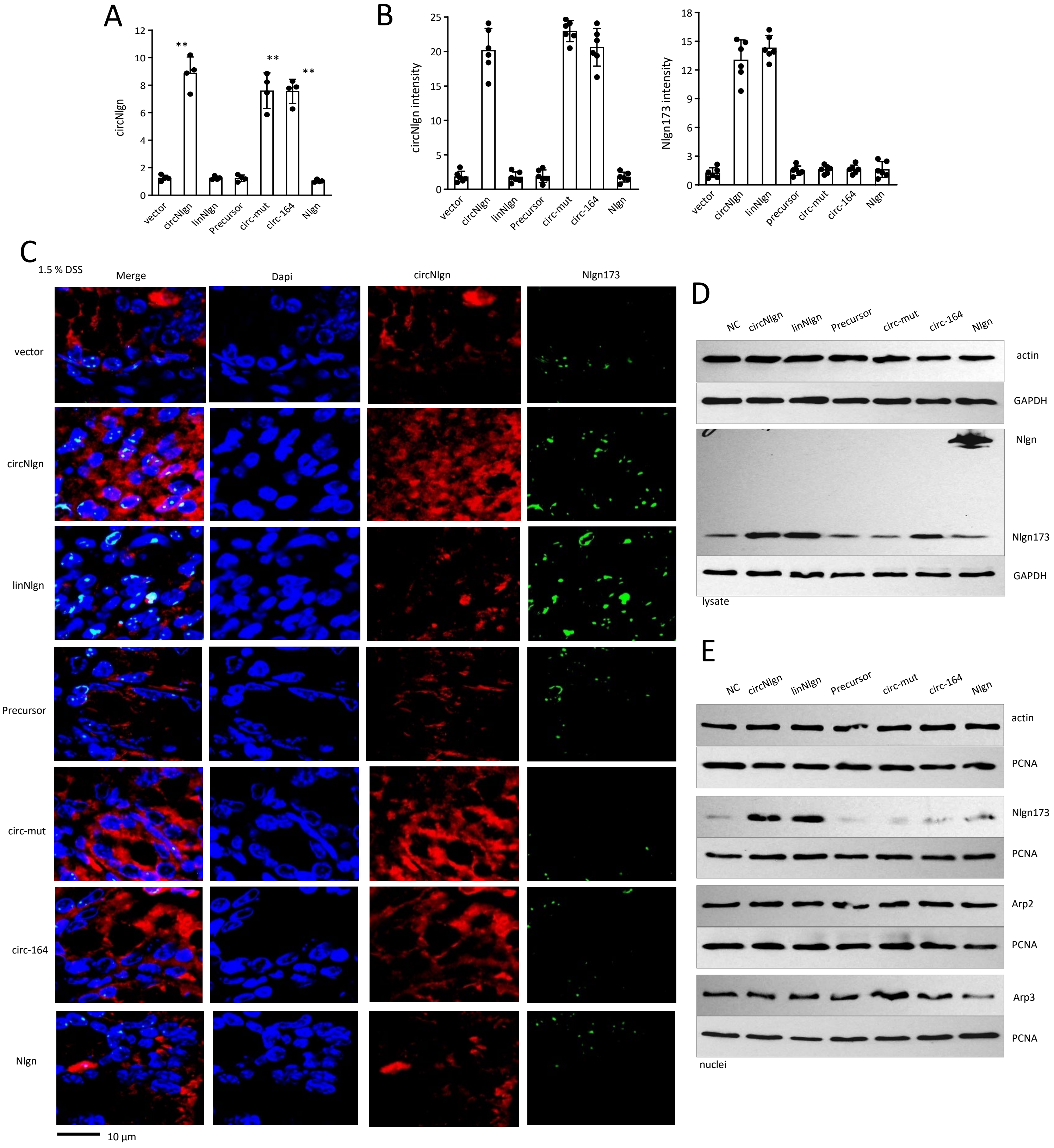

Fig S15. Expression of circNlgn, actin, and Arp2/3 in vivo.
A. RT-PCR showed that circNlgn or linNlgn delivered mouse mucosa expressed higher levels of circNlgn than the control. ***$p<0.01$* versus vector (*n=6*). **B.** Left, ImageJ analysis of in situ hybridization staining showed that circNlgn delivered mouse mucosa expressed higher levels of circNlgn than control. Right, ImageJ analysis of immunofluorescence staining showed that circNlgn or linNlgn delivered mouse mucosa expressed higher levels of Nlgn173 than the control. ***$p<0.01$* versus vector (*n=6*). **C.** Typical images of circNlgn (red) and Nlgn173 (green) staining of the above-treated mouse colon mucosa. **D.** mCECs were isolated from 1.5% DSS treated wild-type mice delivered with control vector, circNlgn, linNlgn, precursor, circ-mut, circ-164, and Nlgn, and subjected to Western blot with an antibody against Nlgn (N-T). **E.** mCECs were subjected to subcellular fractionation and Western blot, showing that circNlgn or linNlgn delivered mCECs presented increased Nlgn173 levels in nuclei.

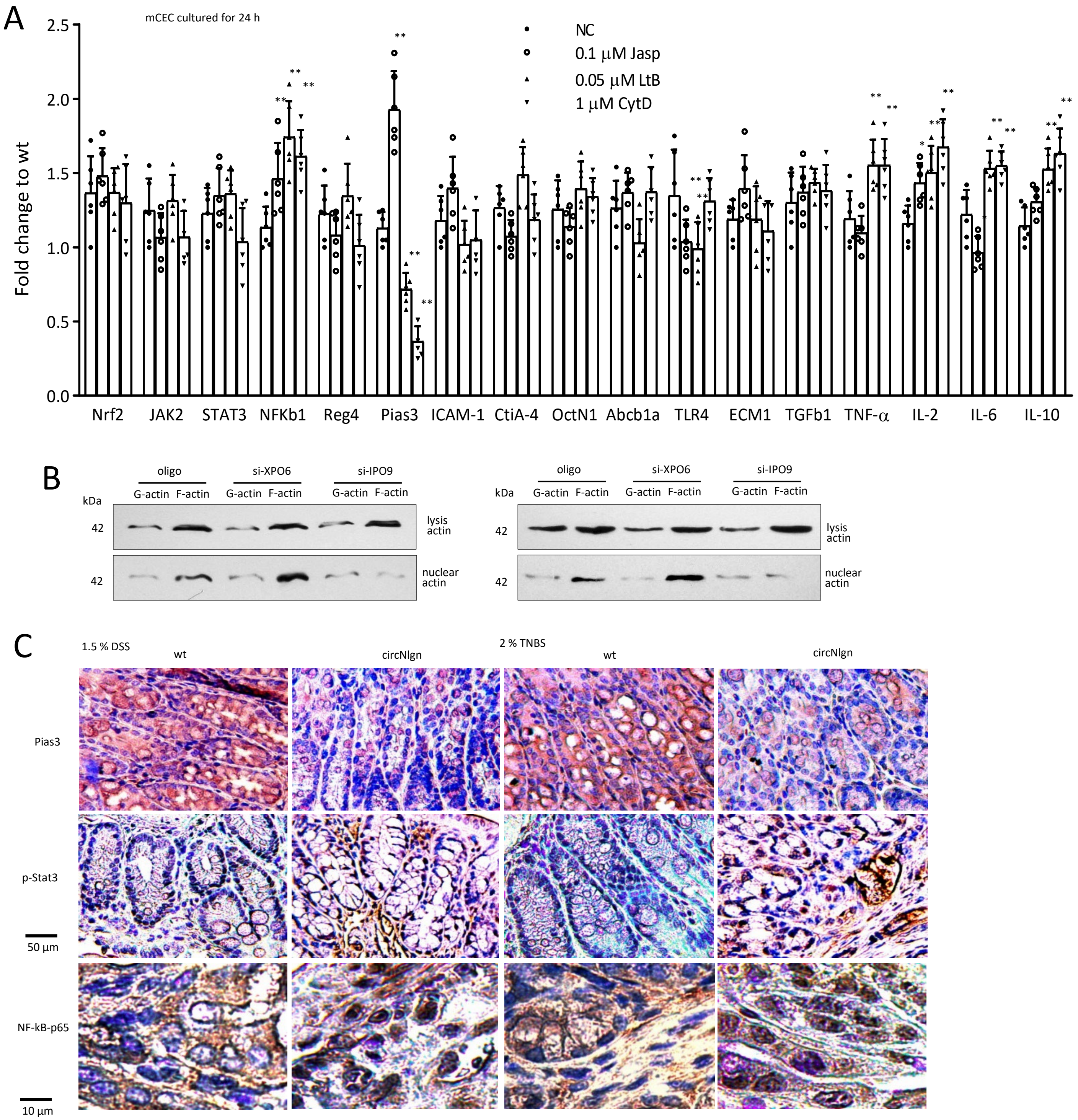

Fig S16. Modulation of Pias3 expression and inflammatory signaling by actin-targeting compounds.

A. mCEC cells were cultured in 0.1 μM jasplakinolide (Jasp), 0.05 μM latrunculin B (LtB) or 1 μM cytochalasin-D (cytD) for 24 h, and subjected to RT-PCR. mCEC cells showed increased Pias3 when treated with Jasp, and expressed decreased pias3 when treated with LtB or CytD. *$p<0.05$, **$p<0.01$* versus NC (*n=6*).

B. Left, FHC cells was transfected with XPO6 or IPO9 siRNAs and cultured in 0.1 μM Jasp for 24 h and subjected to actin fractionation. Western blot showed that silencing XPO6 increased and silencing IPO9 decreased nuclear F-actin levels. Silencing XPO6/IPO9 didn't change actin dynamics in total cell lysate. Right, FHC cells were transfected with XPO6 or IPO9 siRNAs and co-transfected with mDia2. Western blot showed that silencing XPO6 increased and silencing IPO9 decreased nuclear F-actin levels. Silencing XPO6/IPO9 didn't change actin dynamics in total cell lysate.

C. Representative images displayed IHC staining of Pias3, p-Stat3 and NF-kB p65 in the colon mucus of circNlgn transgenic mice after treatment with 1.5% DSS or 2% TNBS.

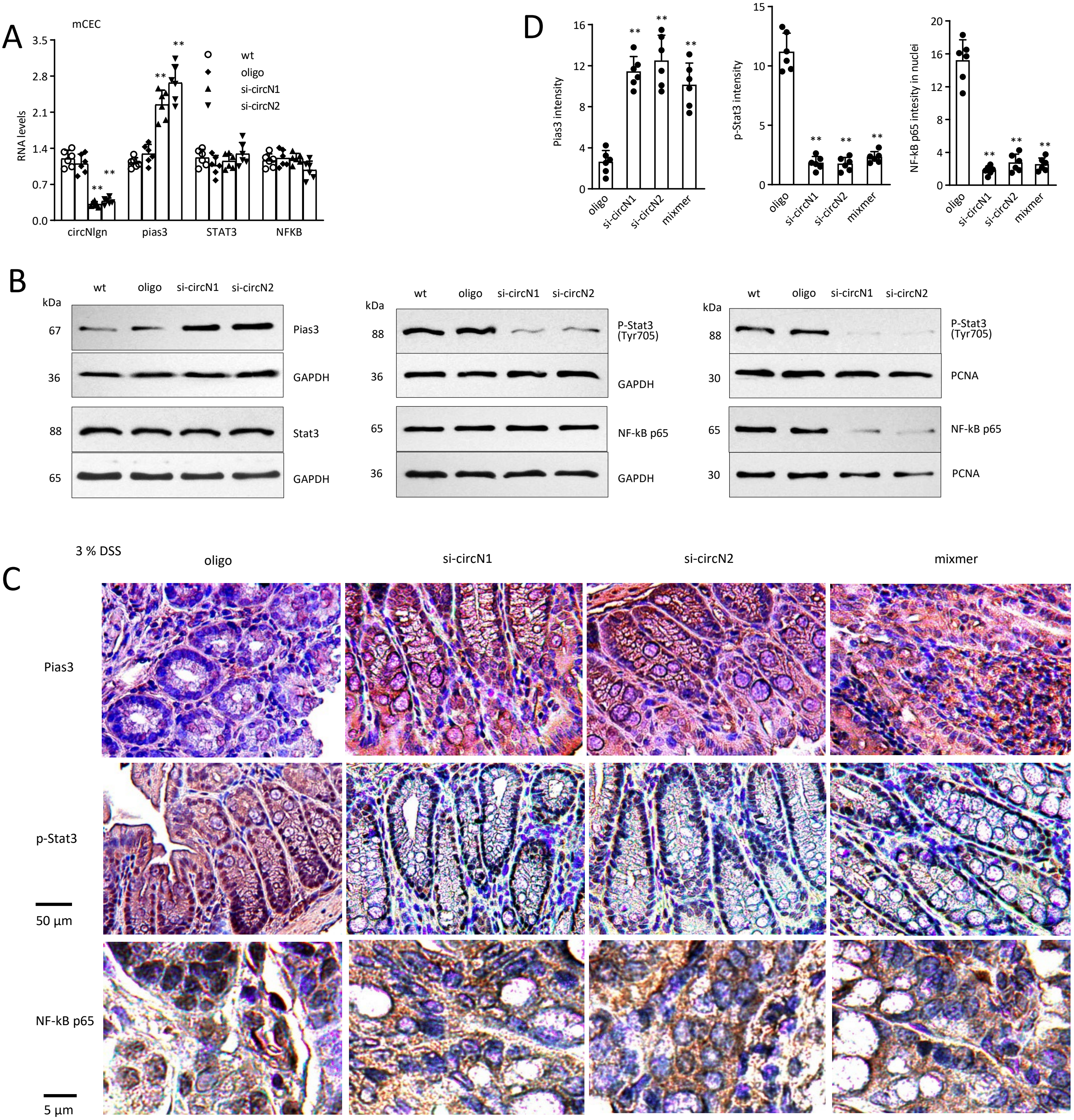

Fig S17. Effect of silencing circNlgn on inflammatory signaling.

A. mCEC cells were transfected with circNlgn siRNAs. Silencing circNlgn with siRNAs enhanced expression levels of Pias3 of mCEC cells. ***p<0.01* versus oligo (*n=6)*.

B. Western blot showing expression of Pias3, p-Stat3 and NF-kB p65 in mouse colon mucus receiving circNlgn siRNAs or mixmer after treated with 3 % DSS.

C. Typical images showed IHC staining of Pias3, p-Stat3 and NF-kB p65 in mouse colon mucus receiving circNlgn siRNAs or mixmer after treated with 3 % DSS.

D. ImageJ analysis demonstrated that mice treated with circNlgn siRNAs or mixmer displayed increased Pias3 levels and decreased p-Stat3 in the colon mucus, and decreased NF-kB p65 in cell nuclei. ***p<0.01* versus oligo (*n=6)*.
