# Supplementary material for: Aggravated Ulcerative Colitis via circNlgn-Mediated Suppression of Nuclear Actin Polymerization
Source: Research (Wash D C). 2024 Aug 23;7:0441. doi: 10.34133/research.0441 (PMC11342054; doi:10.34133/research.0441)
Supplement: Supplementary 1 — Supplementary Materials and Methods Figs. S1 to S17 Tables S1 and S2 [file research.0441.f1.zip › Colitis-Original gel blots-July 2, 2023.pdf]

Original Western blots

Fig 1G

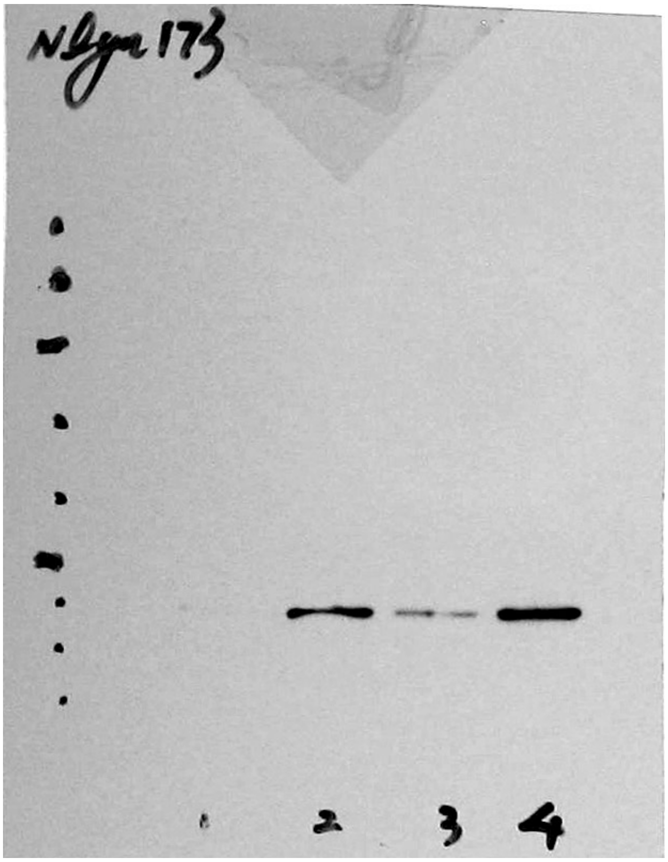

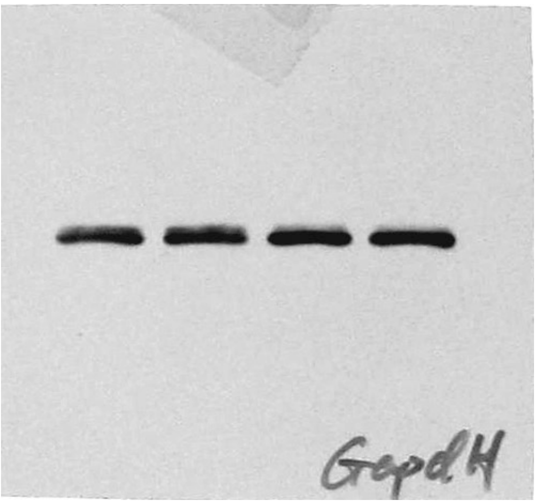

Fig 5C

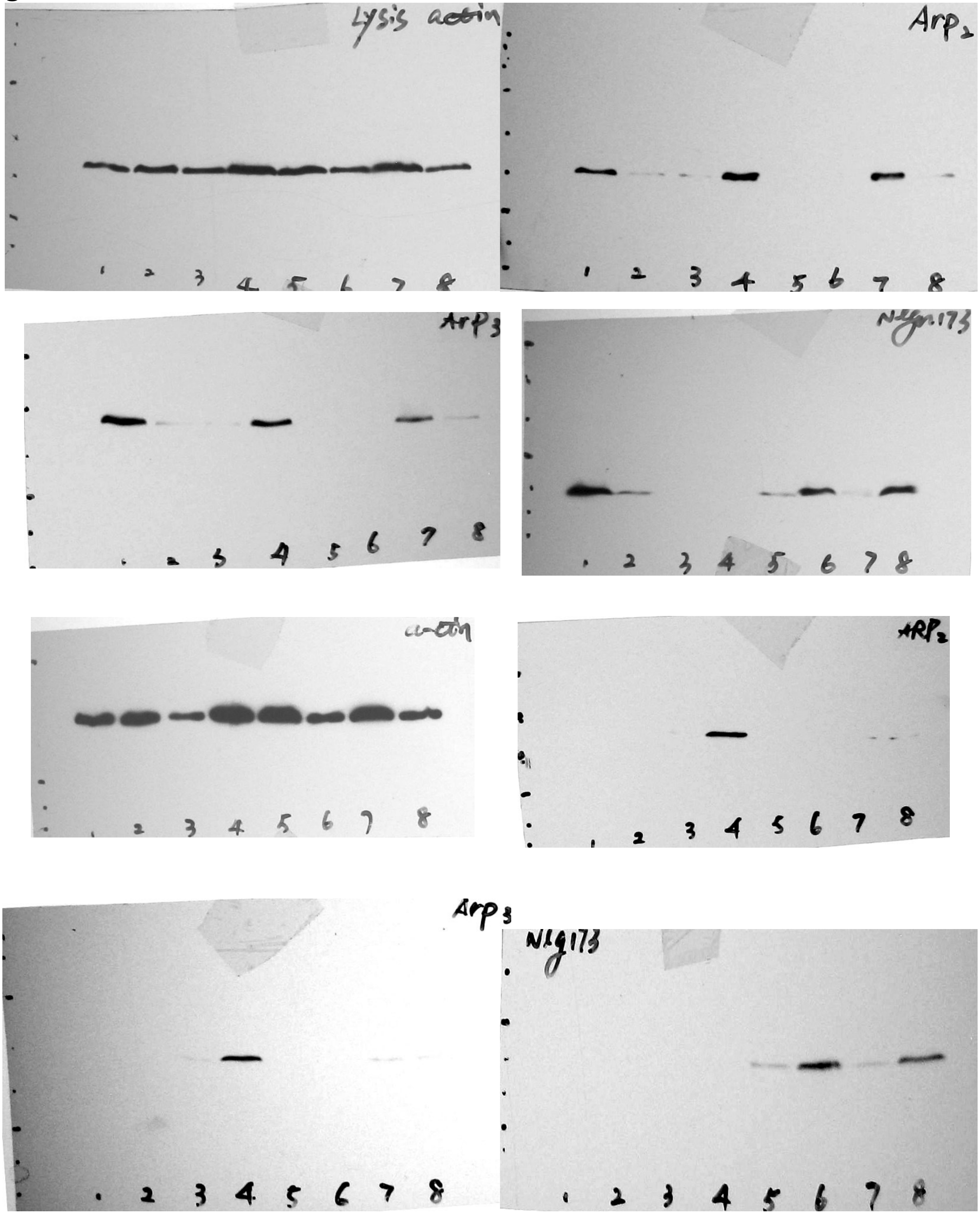

Lysis actin
1 2 3 4 5 6 7 8
Arp2
1 2 3 4 5 6 7 8
Arp3
1 2 3 4 5 6 7 8
1 2 3 4 5 6 7 8
actin
1 2 3 4 5 6 7 8
ARP2
1 2 3 4 5 6 7 8
Arp3
1 2 3 4 5 6 7 8
1 2 3 4 5 6 7 8

Fig 5D

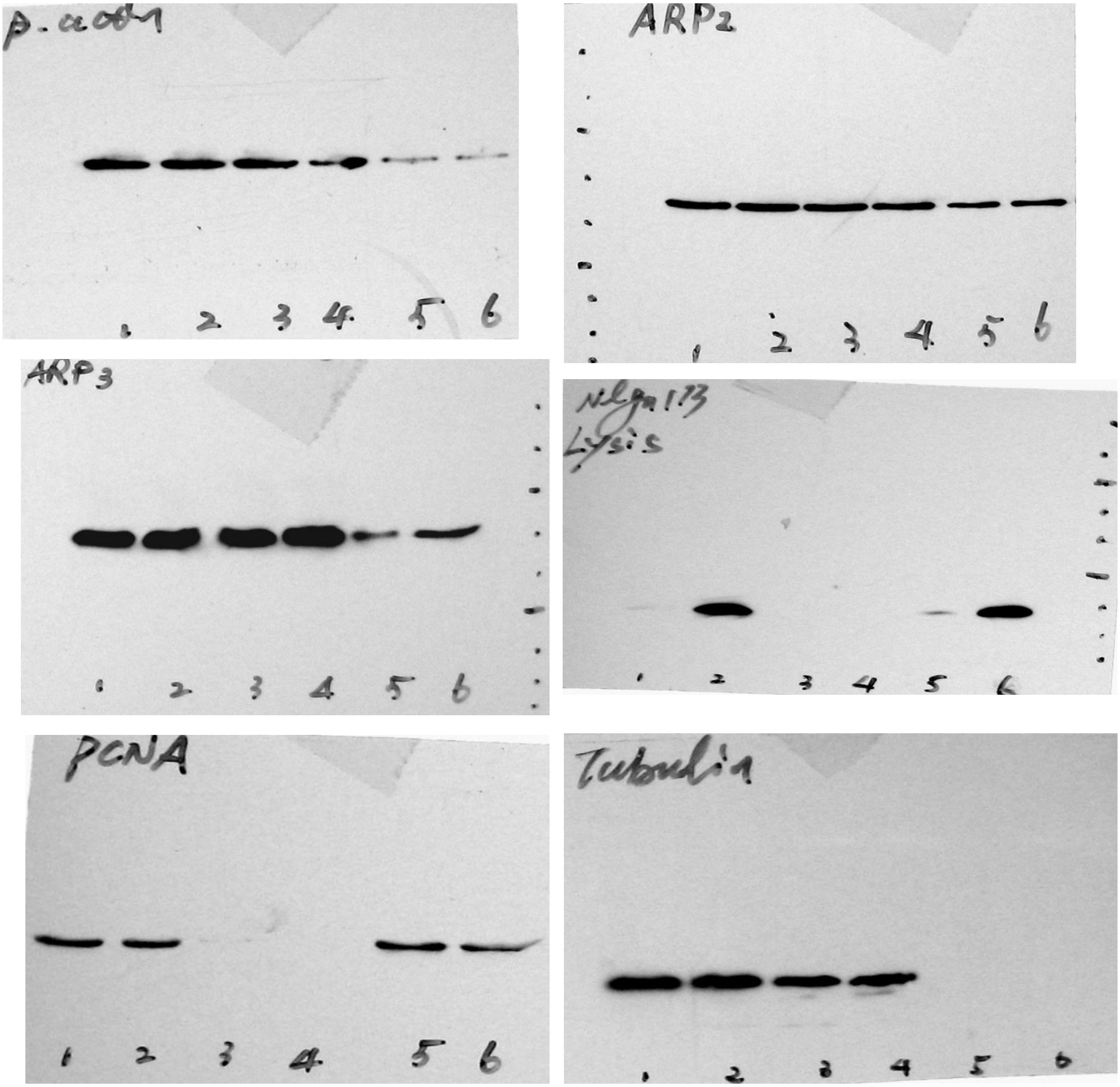
β-actin
1 2 3 4 5 6
ARP2
1 2 3 4 5 6
ARP3
1 2 3 4 5 6
Lysis
1 2 3 4 5 6
PCNA
1 2 3 4 5 6
Tubulin
1 2 3 4 5 6

Fig 5D

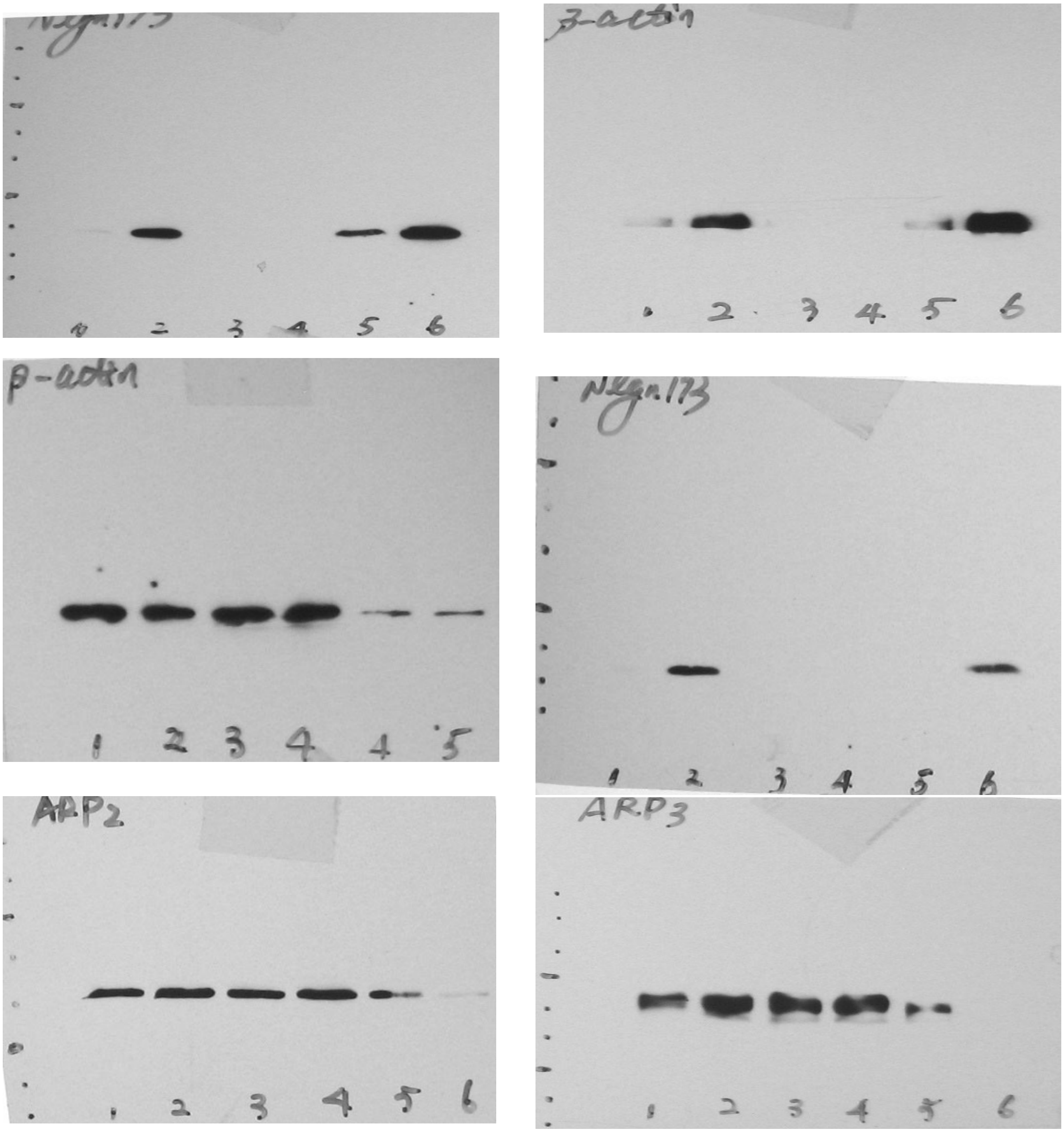

Fig 5D

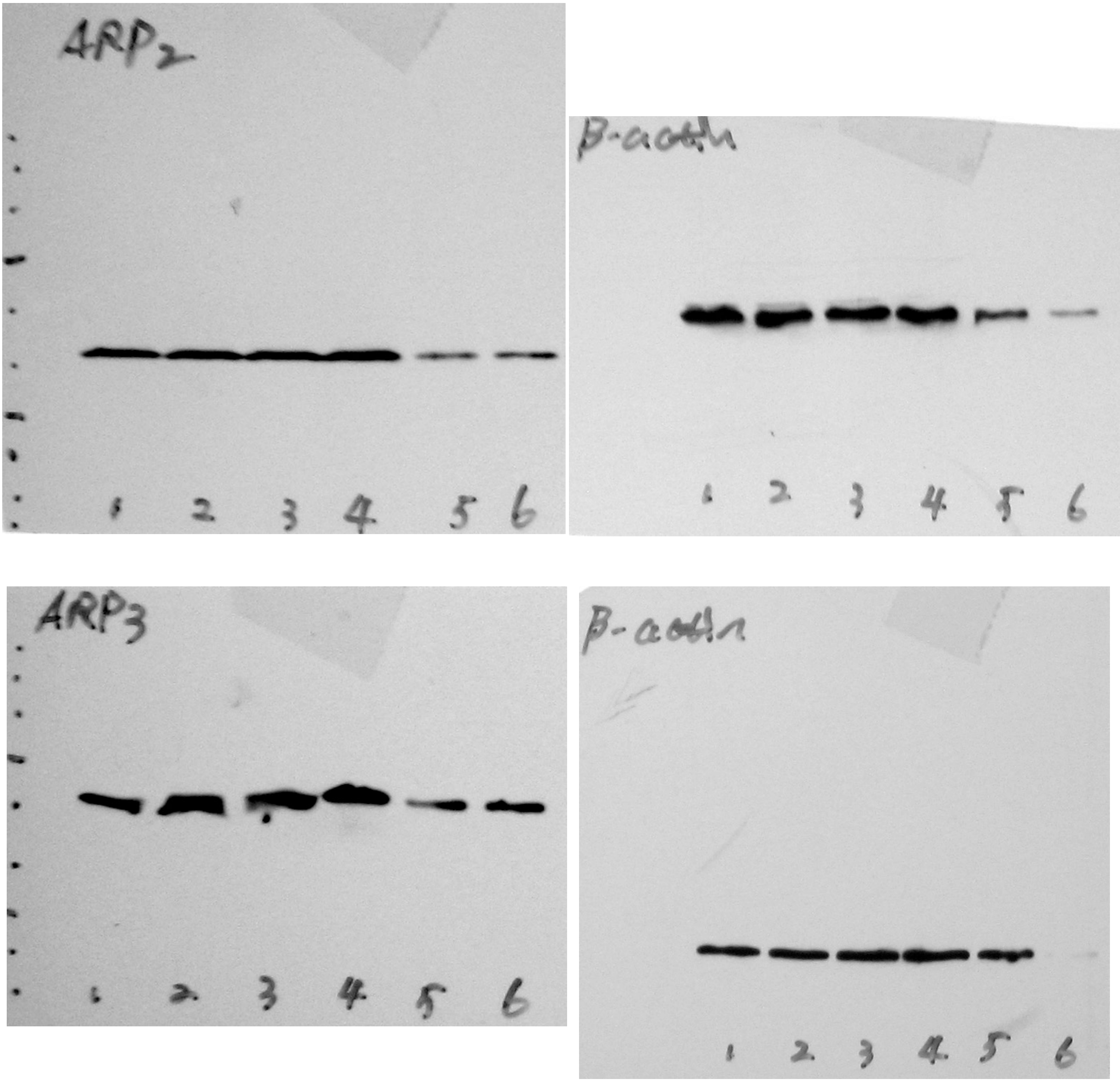

Fig 5E

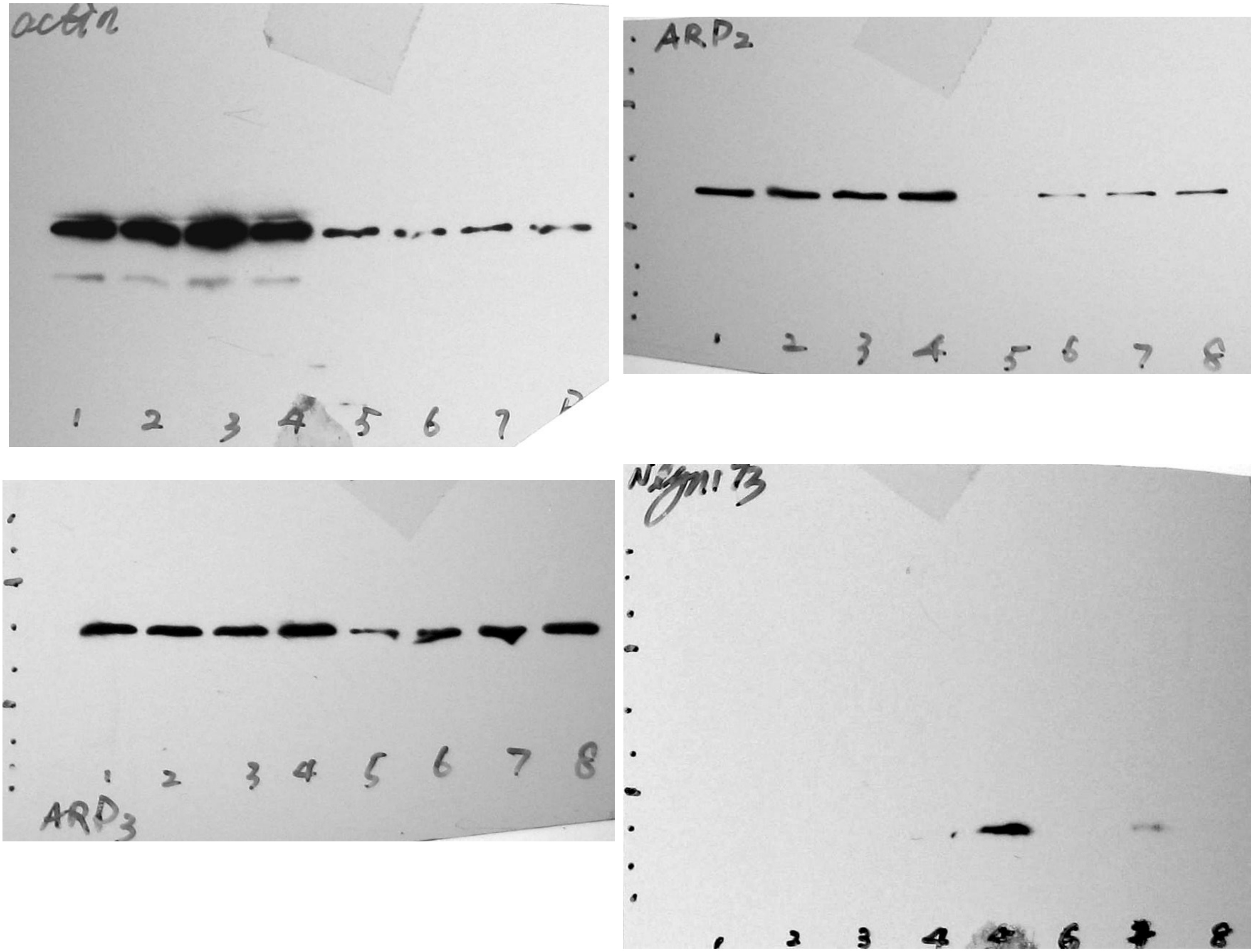

Fig 5E

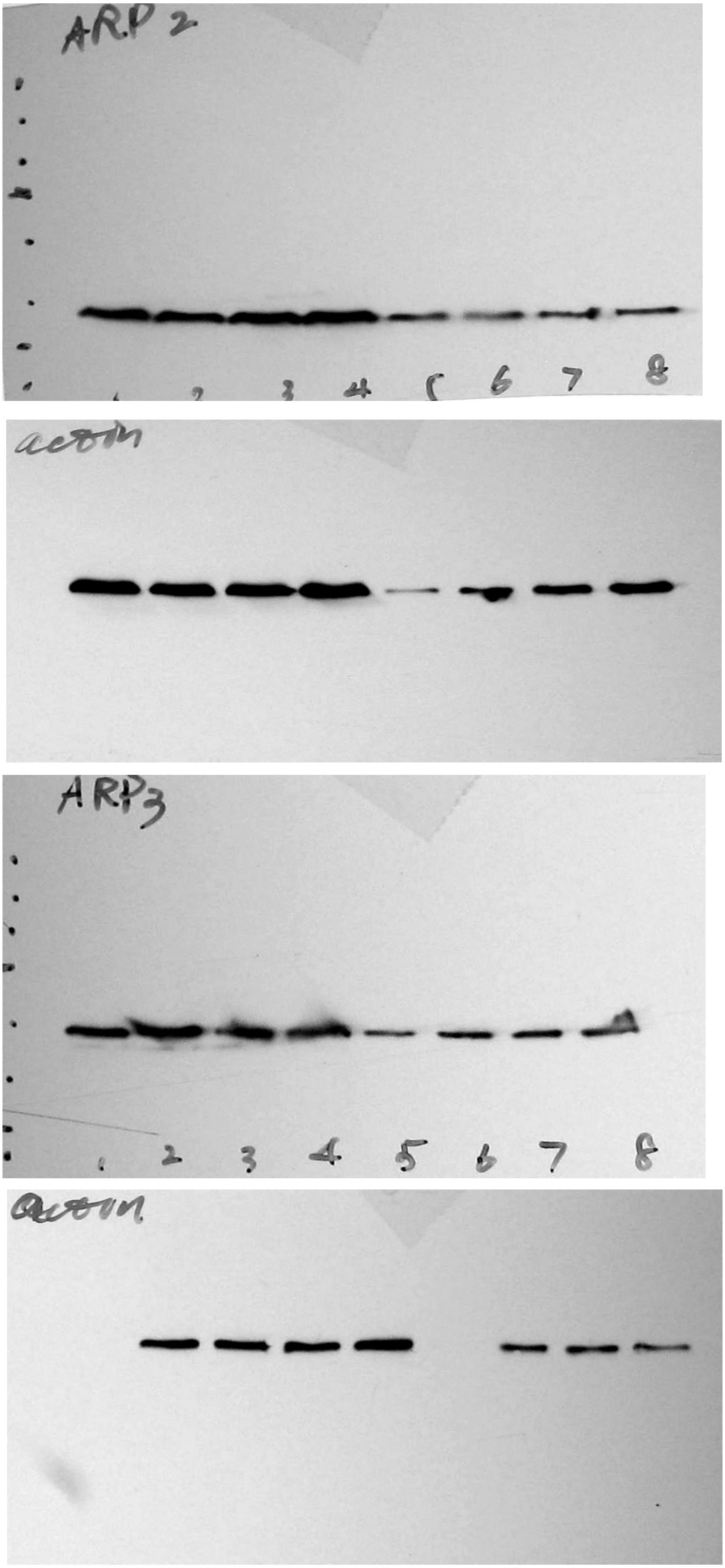

Fig 6A

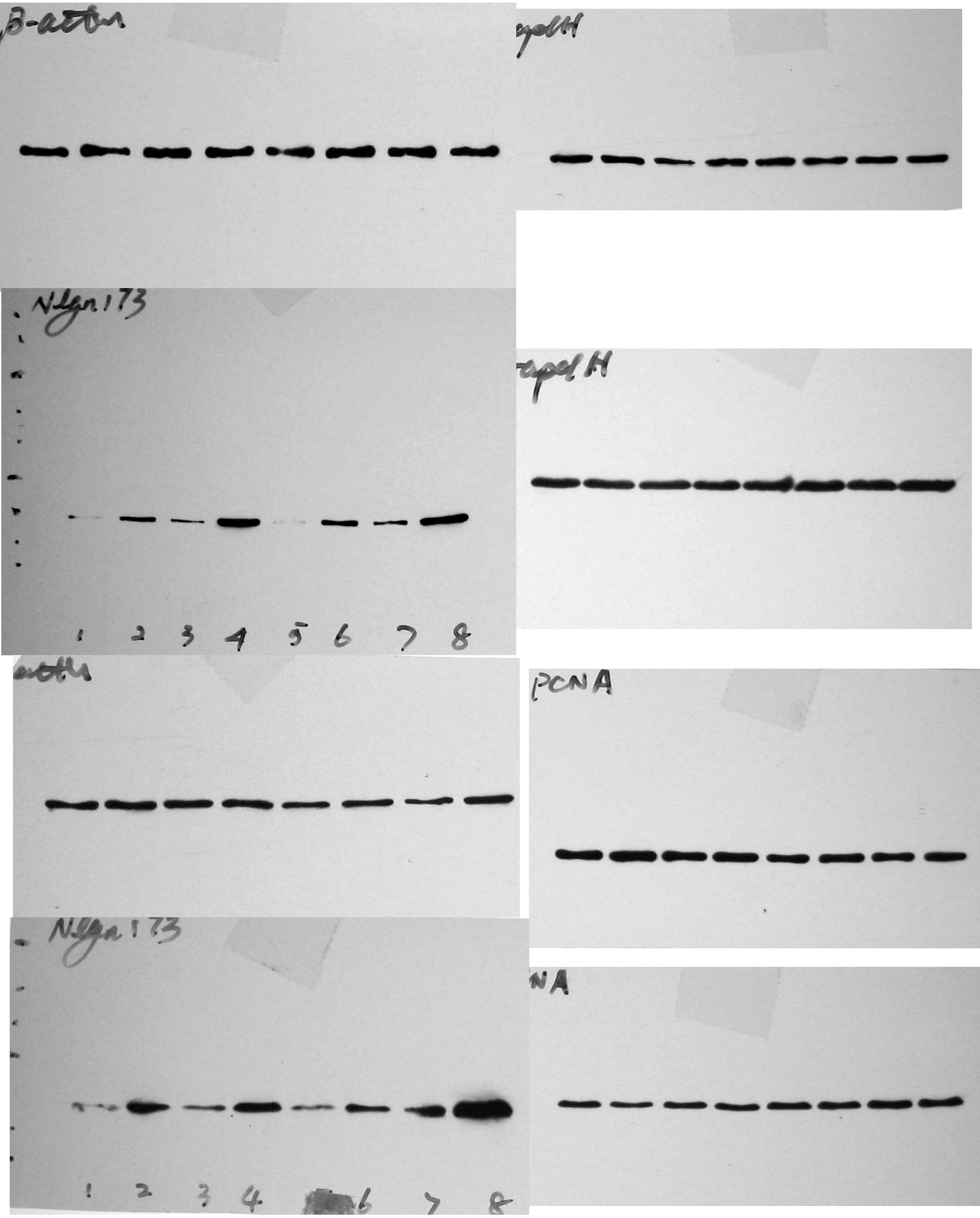

Fig 6A

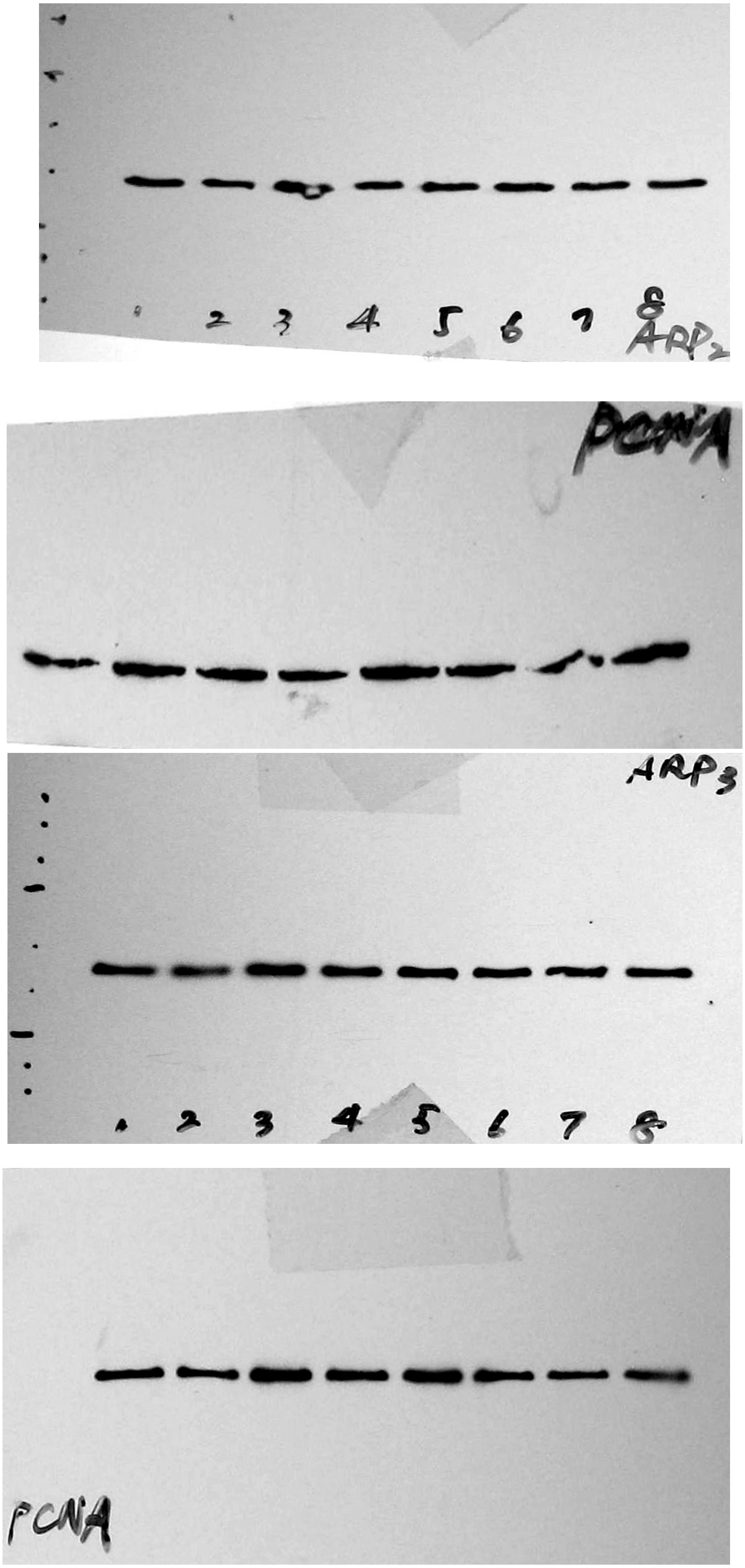

Fig 6B

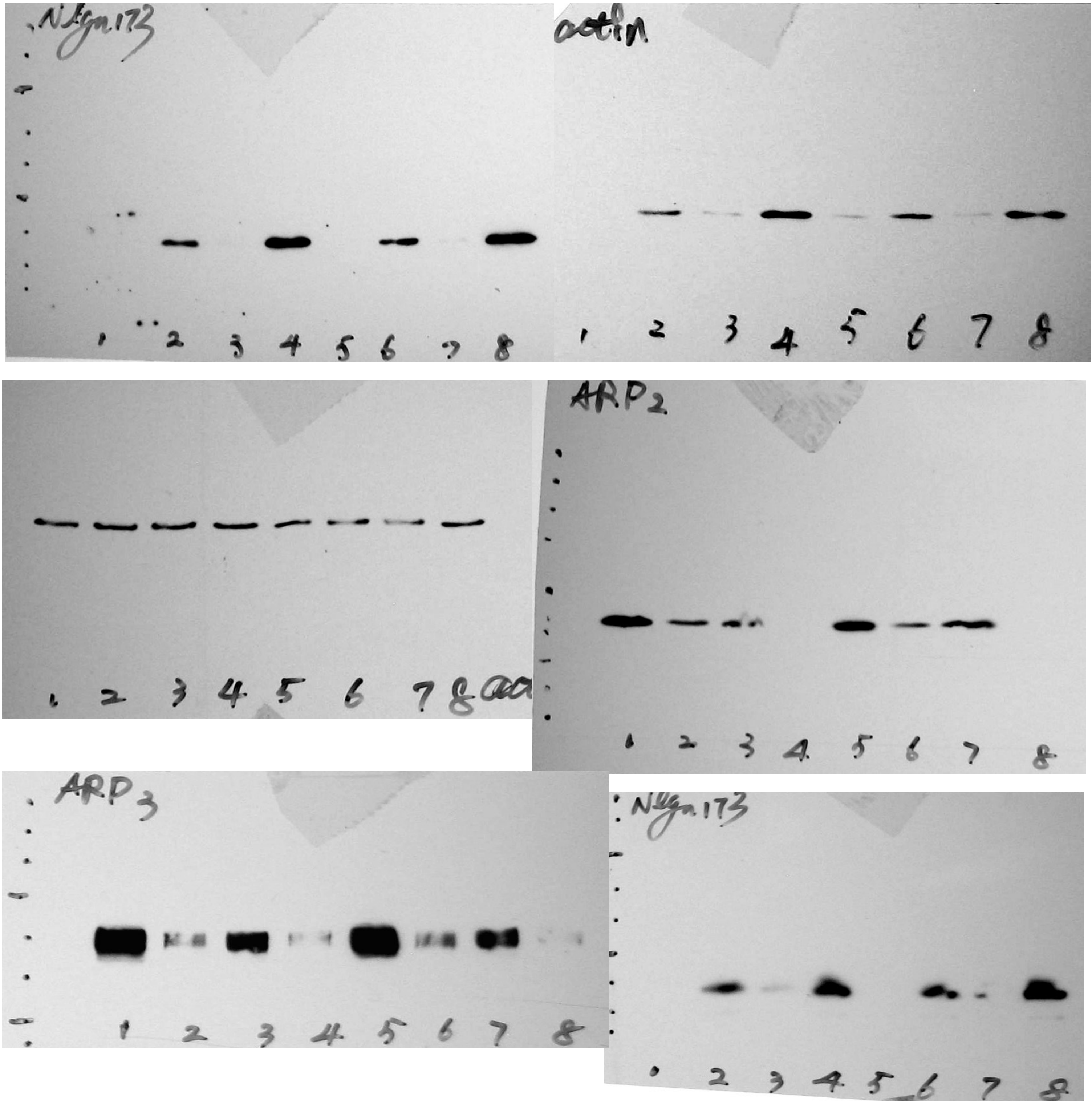

Fig 6B

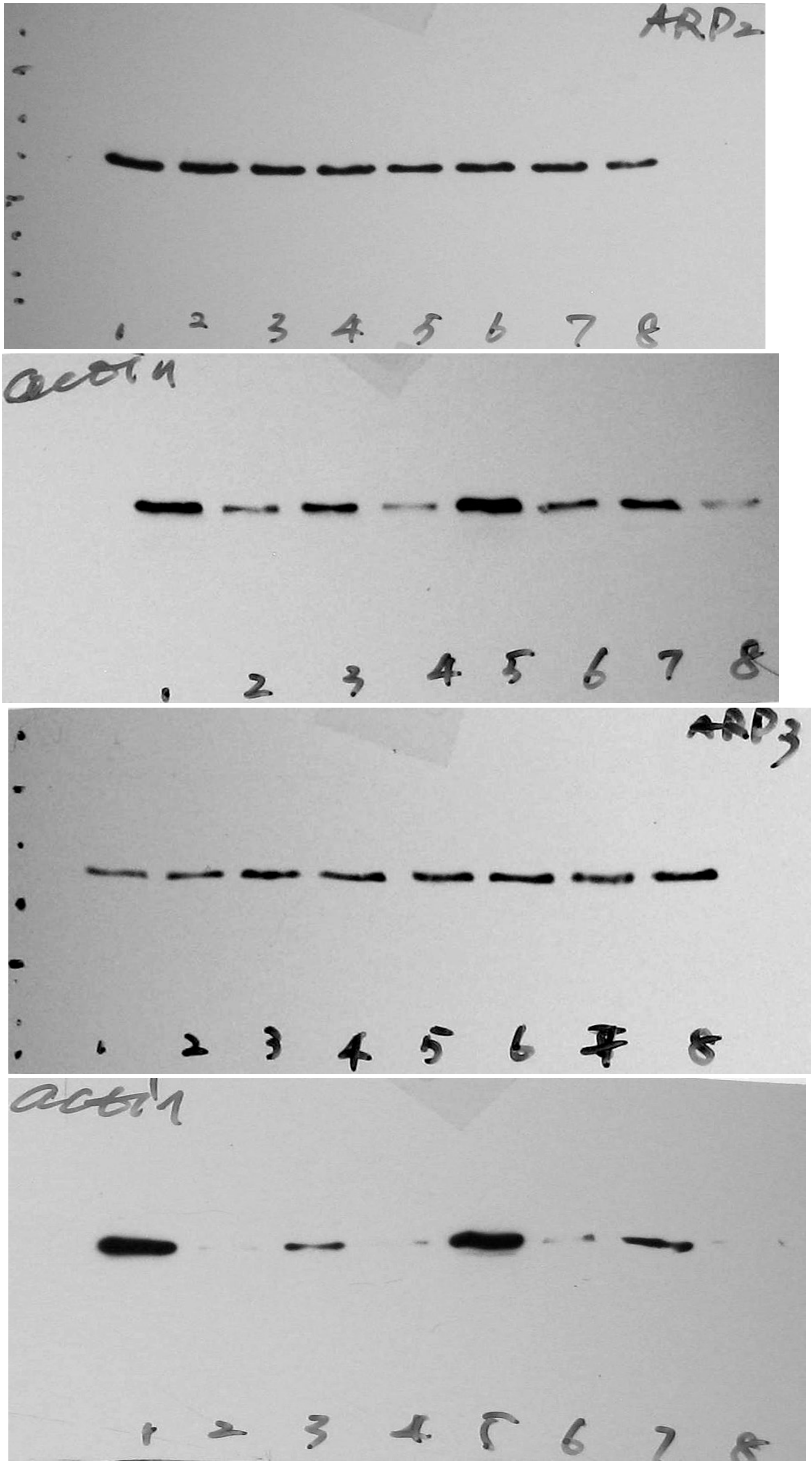
ARP2
1 2 3 4 5 6 7 8
actin
1 2 3 4 5 6 7 8
ARP3
1 2 3 4 5 6 7 8
actin
1 2 3 4 5 6 7 8

Fig 6C

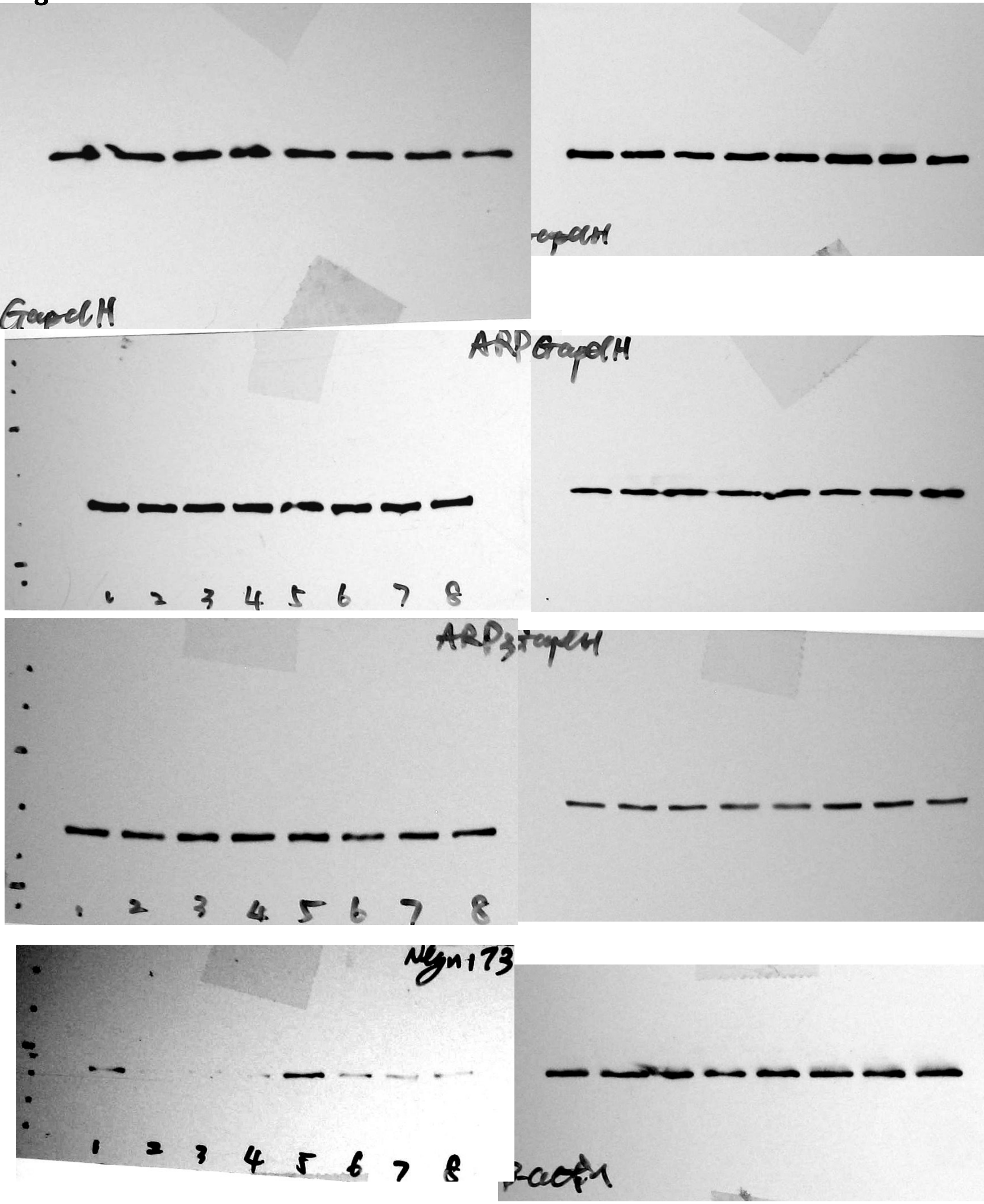

Fig 6C

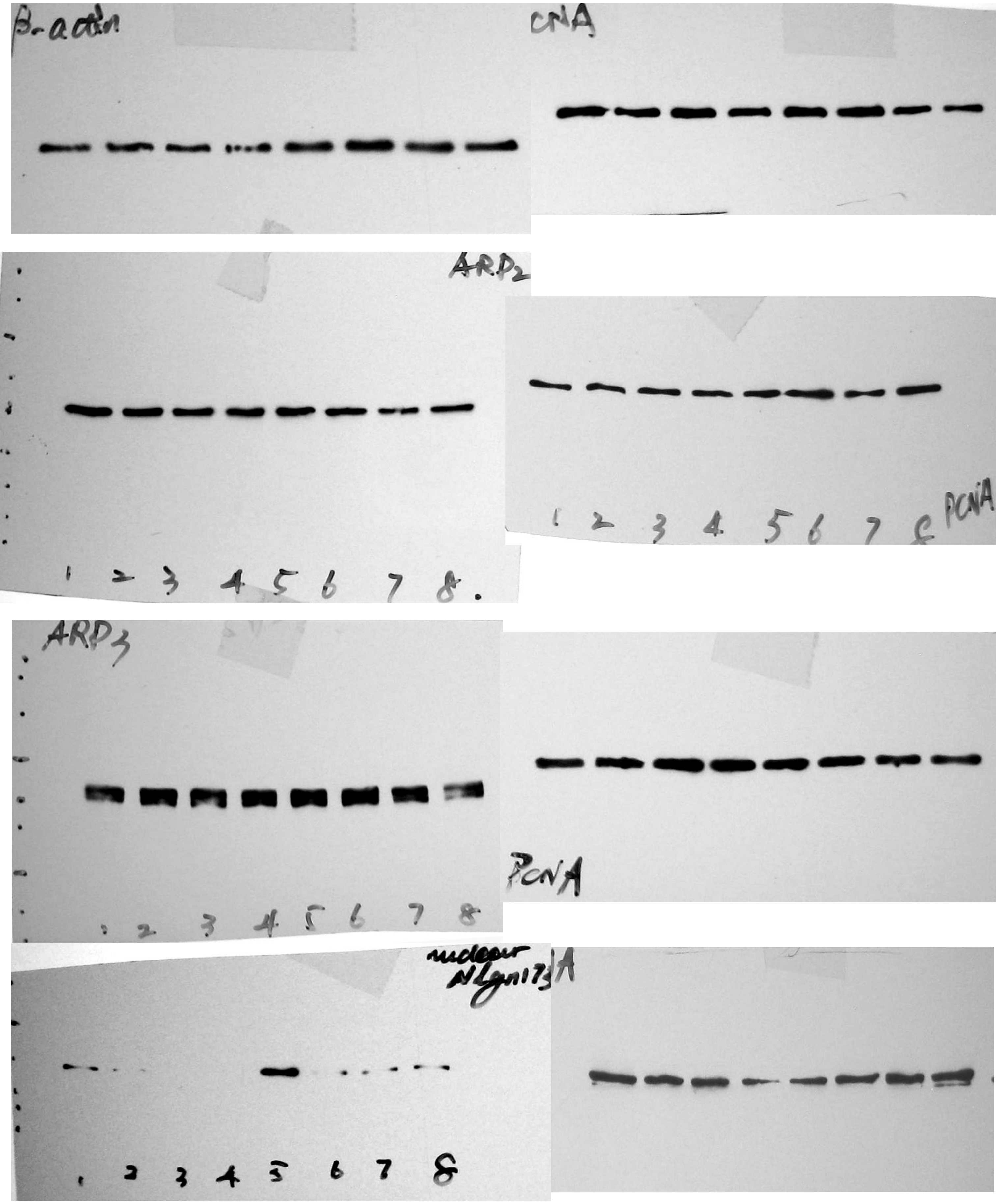
β-actin
ARP2
1 2 3 4 5 6 7 8
PCNA
1 2 3 4 5 6 7 8
ARP3
1 2 3 4 5 6 7 8
PCNA
1 2 3 4 5 6 7 8

Fig 6D

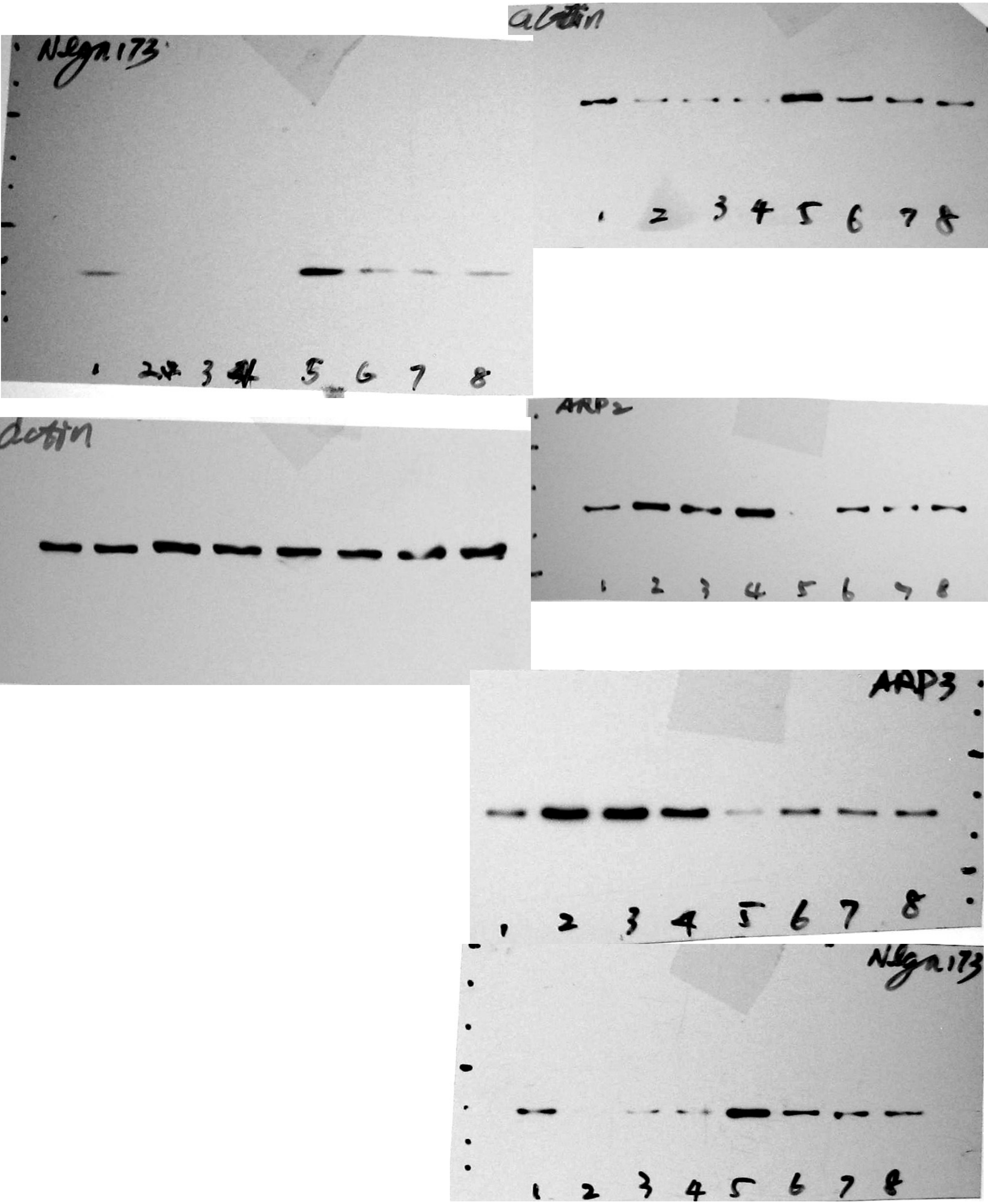

Fig 6D

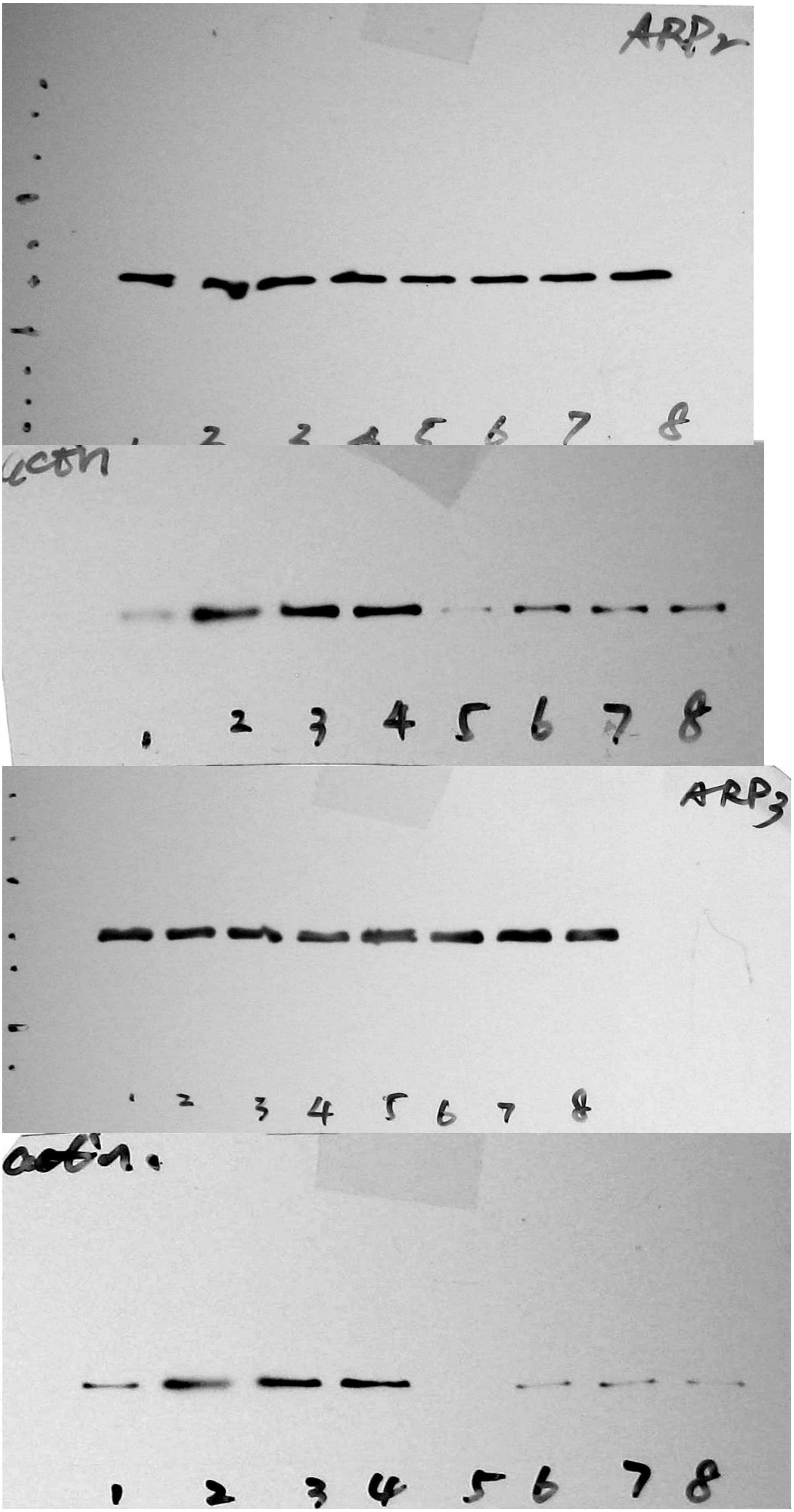

Fig 7H

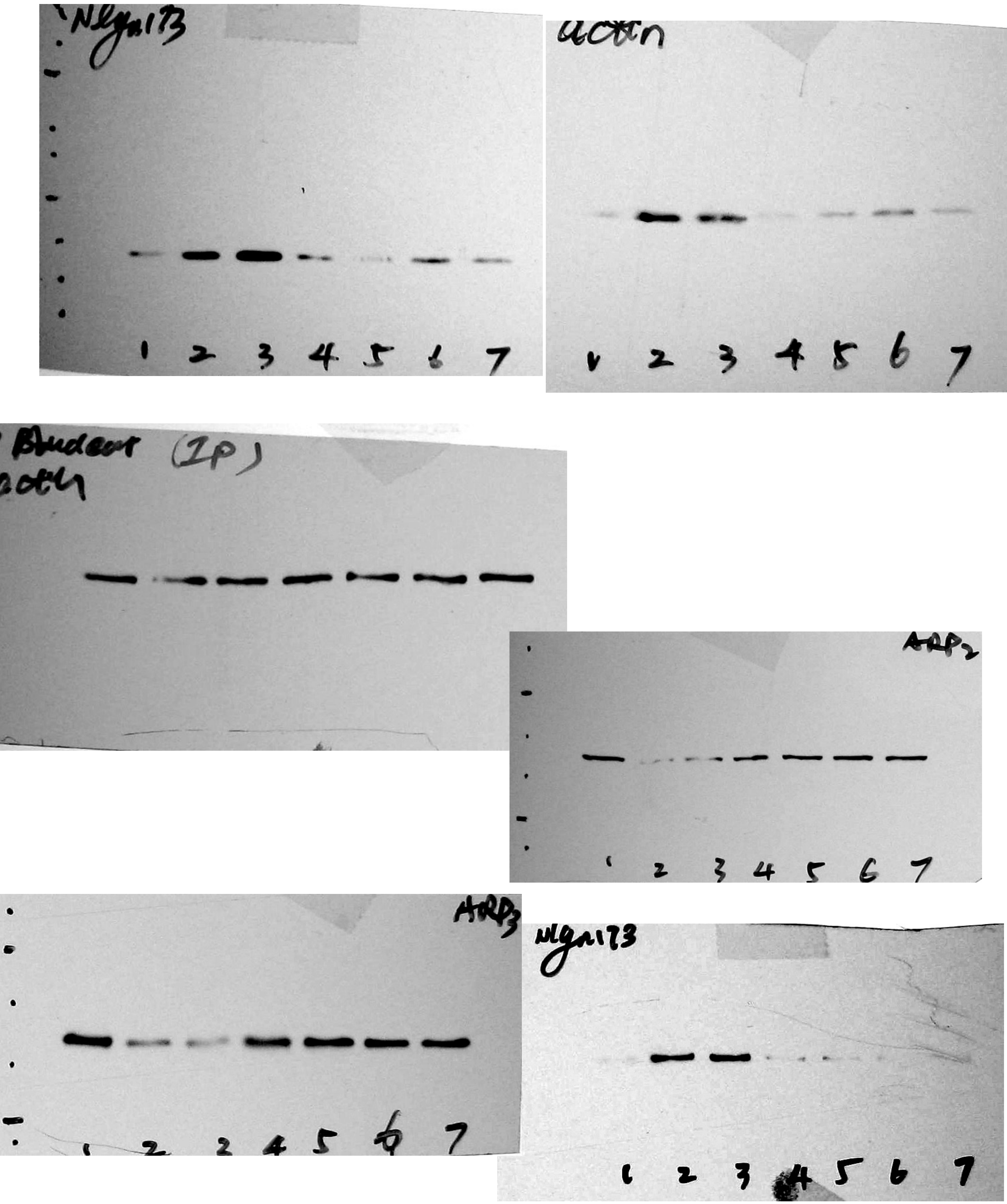

Fig 7H

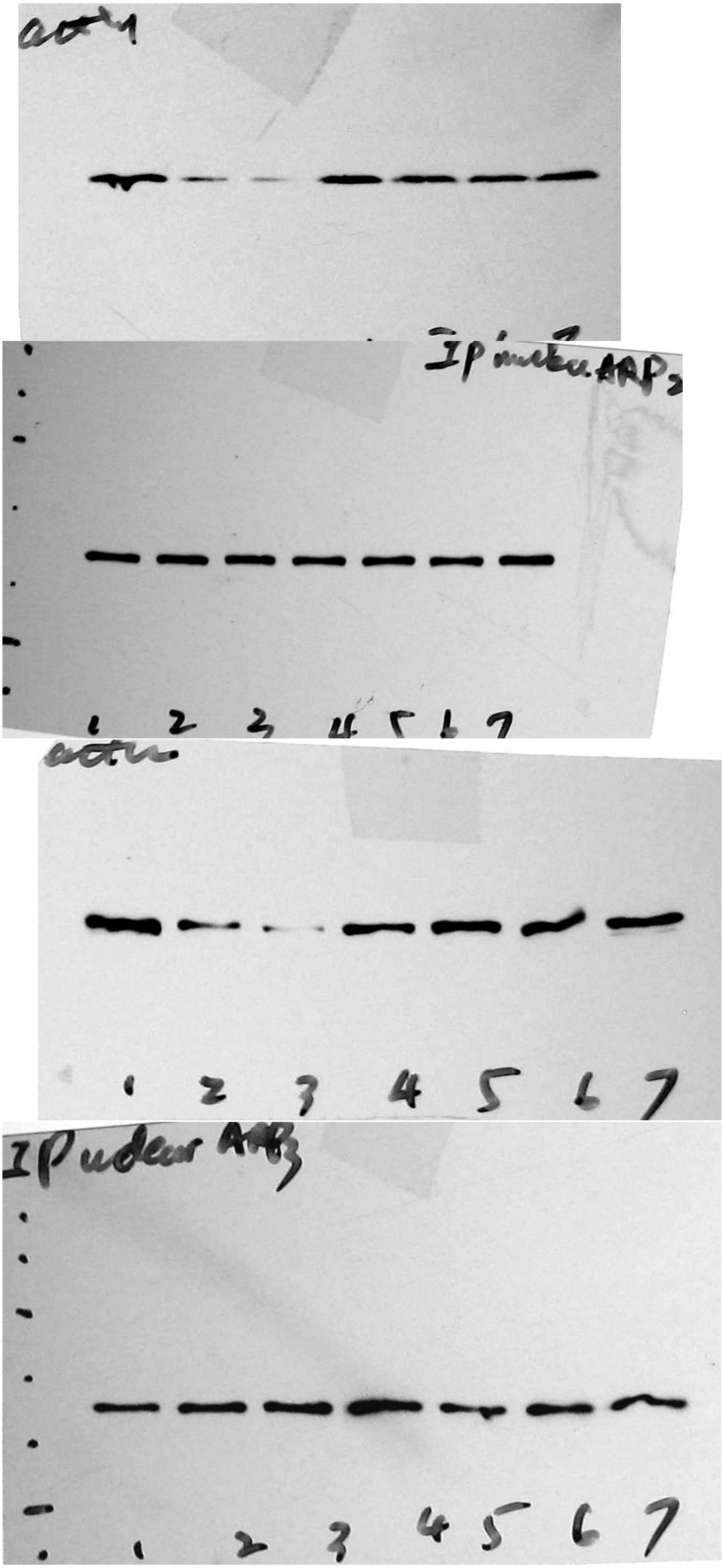

Fig 8A

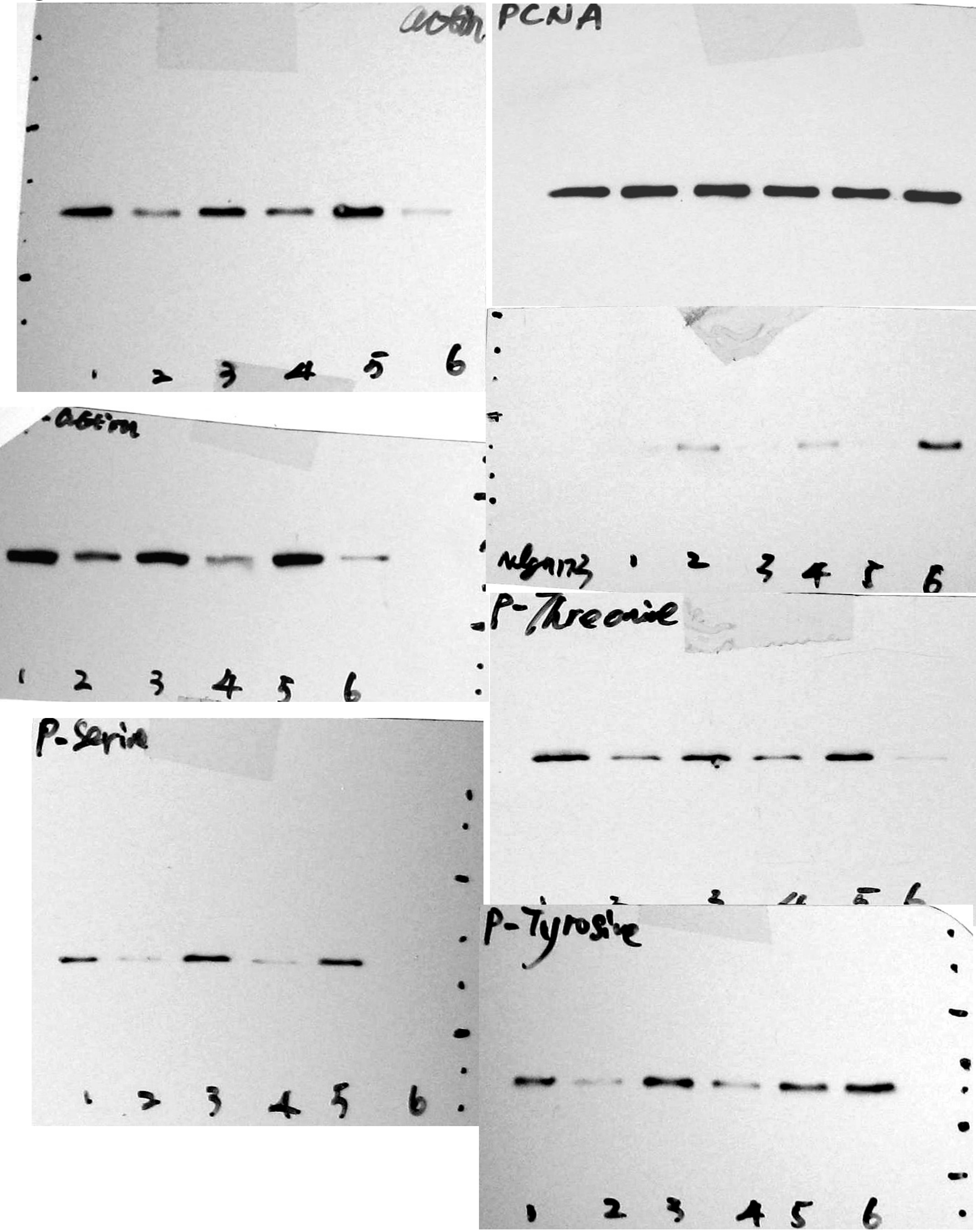

Fig 8B

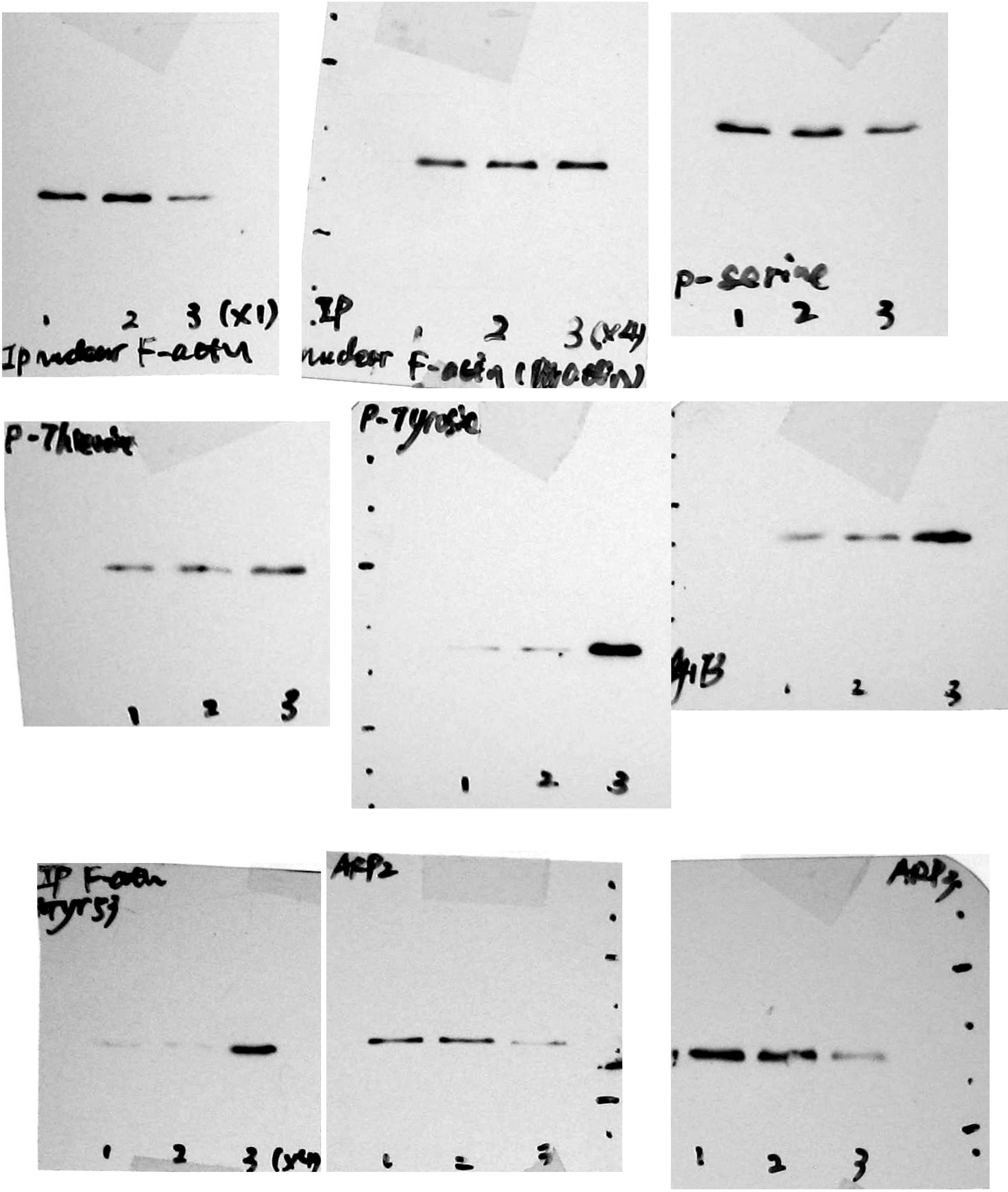

Fig 8C

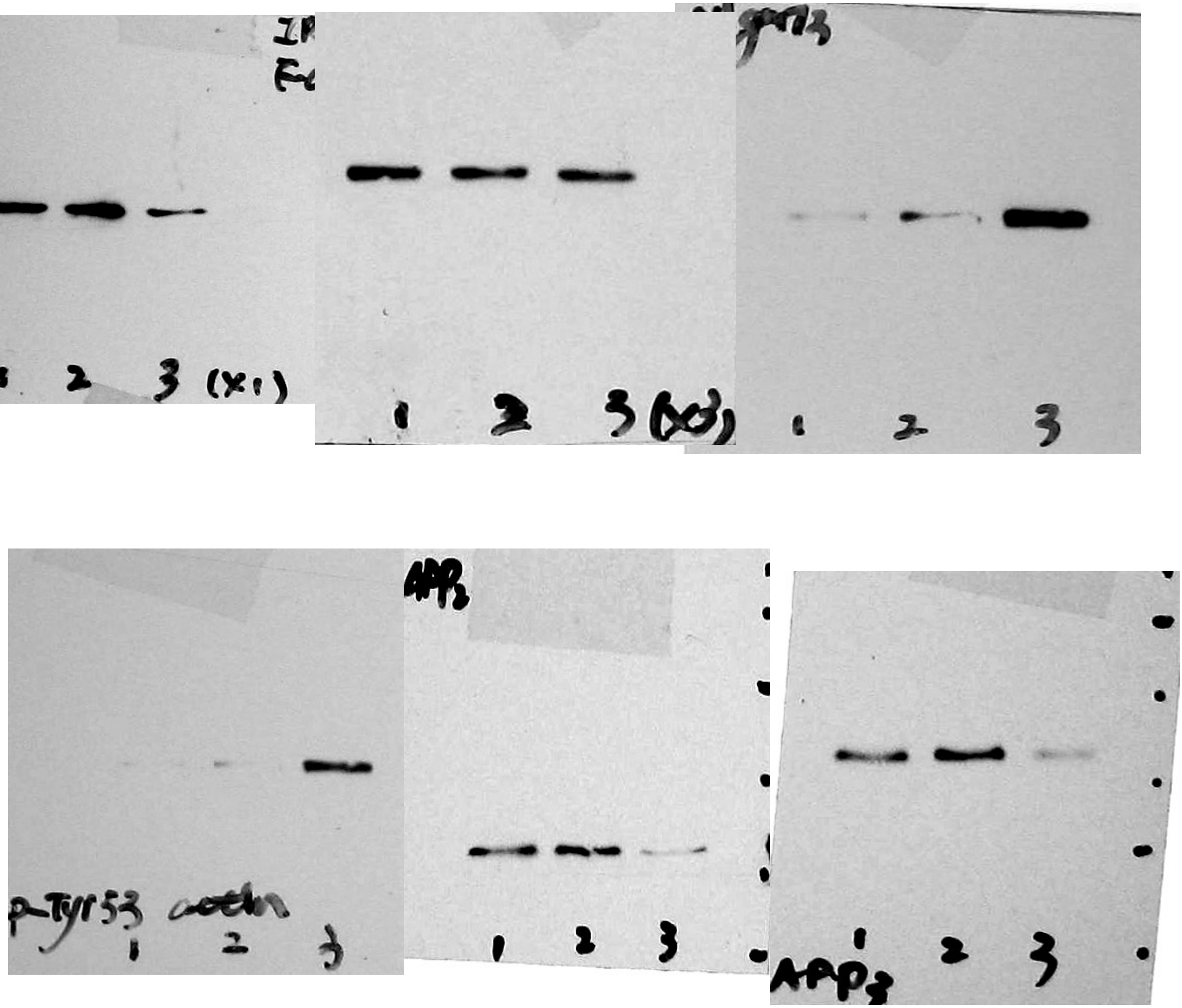

Fig 8D

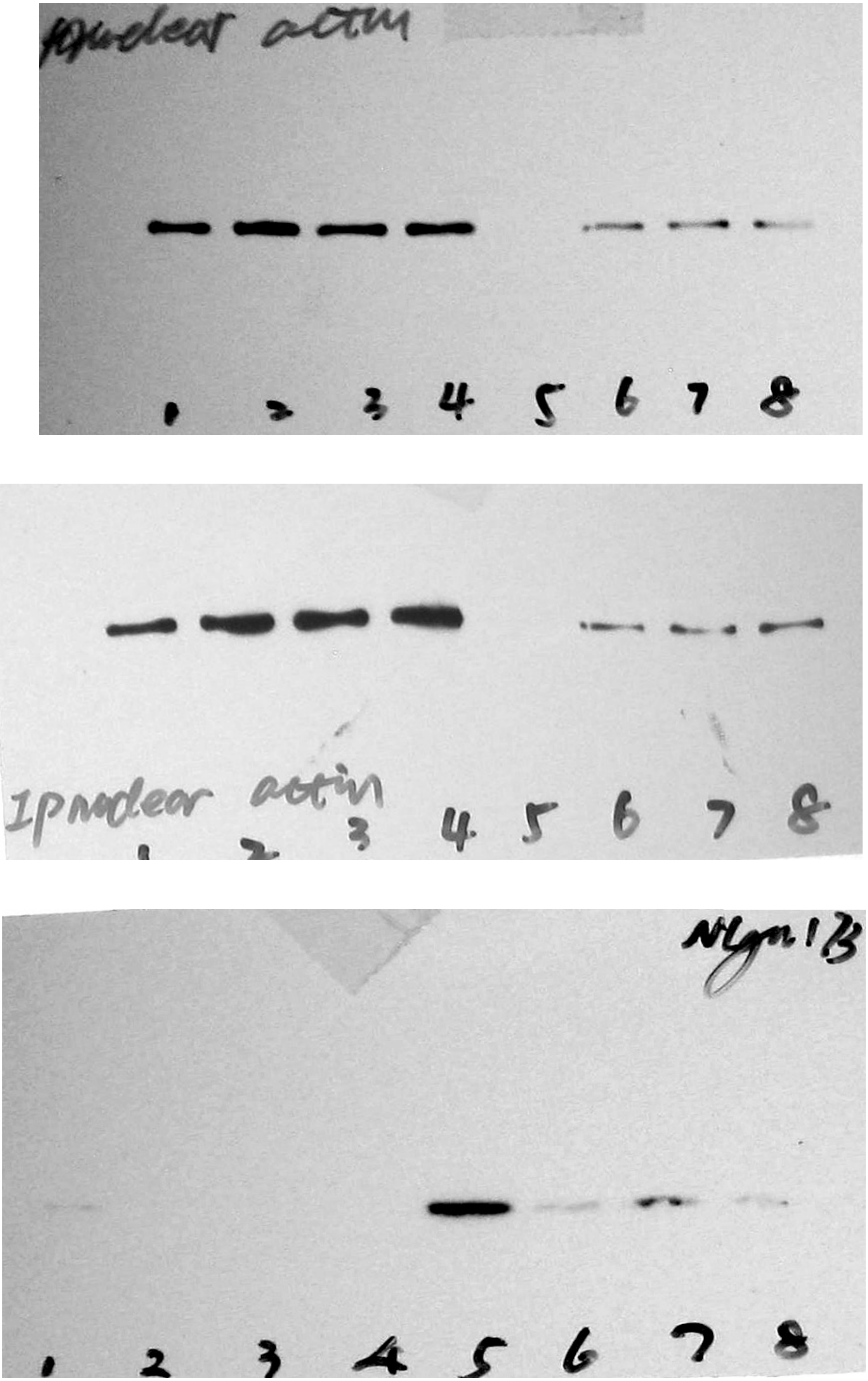

Fig 8E

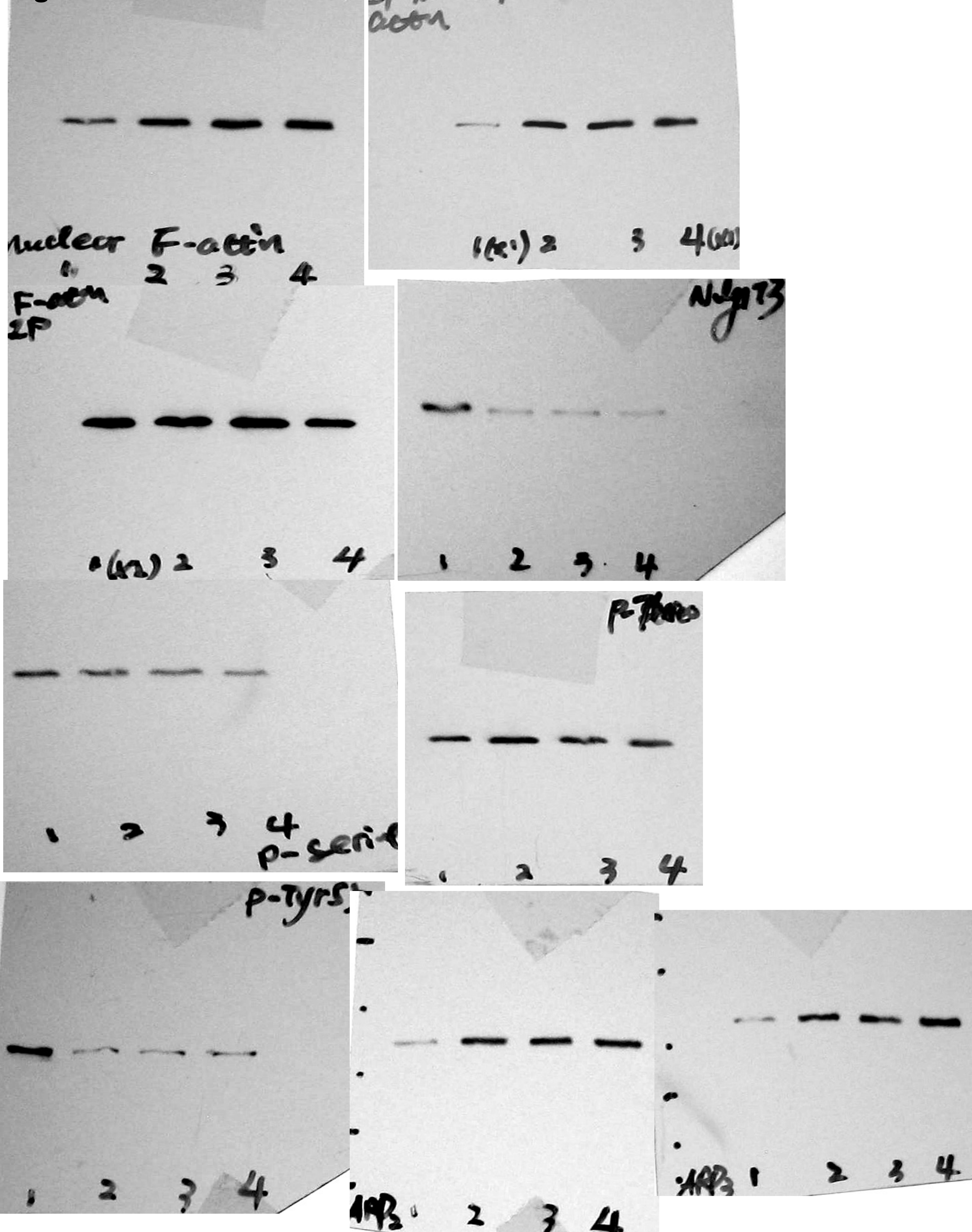

Fig 8F

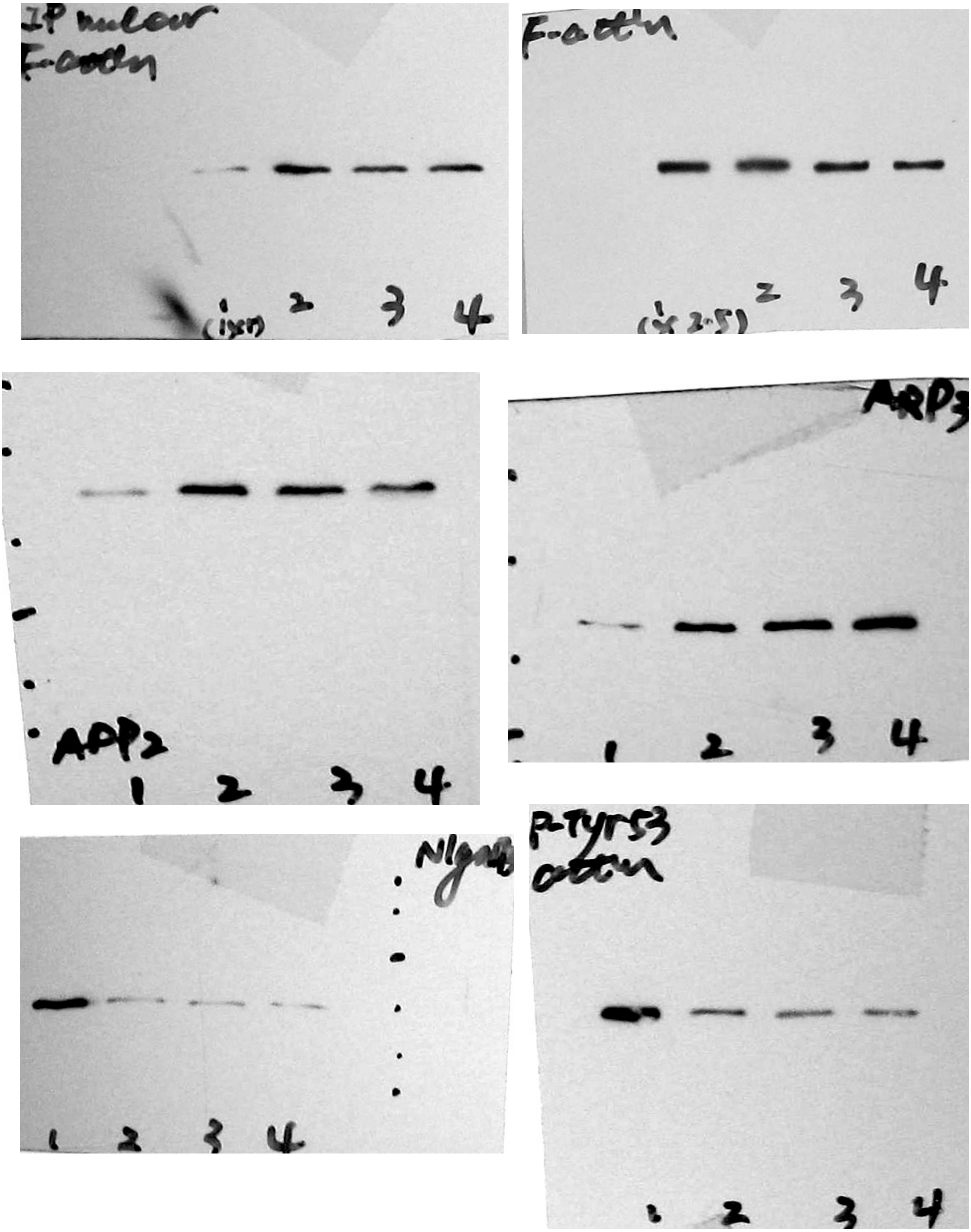

Fig 8G

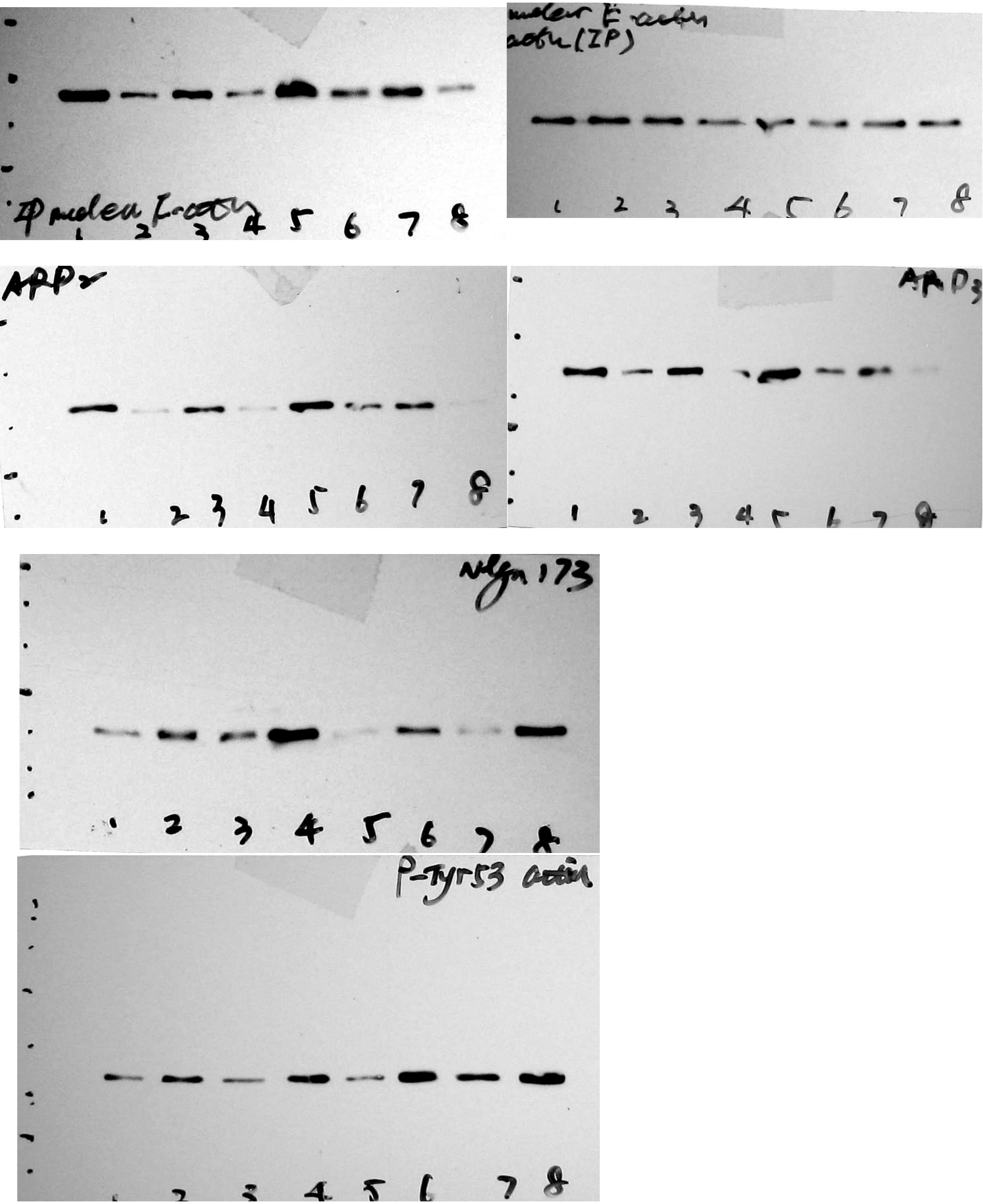

Fig 8H

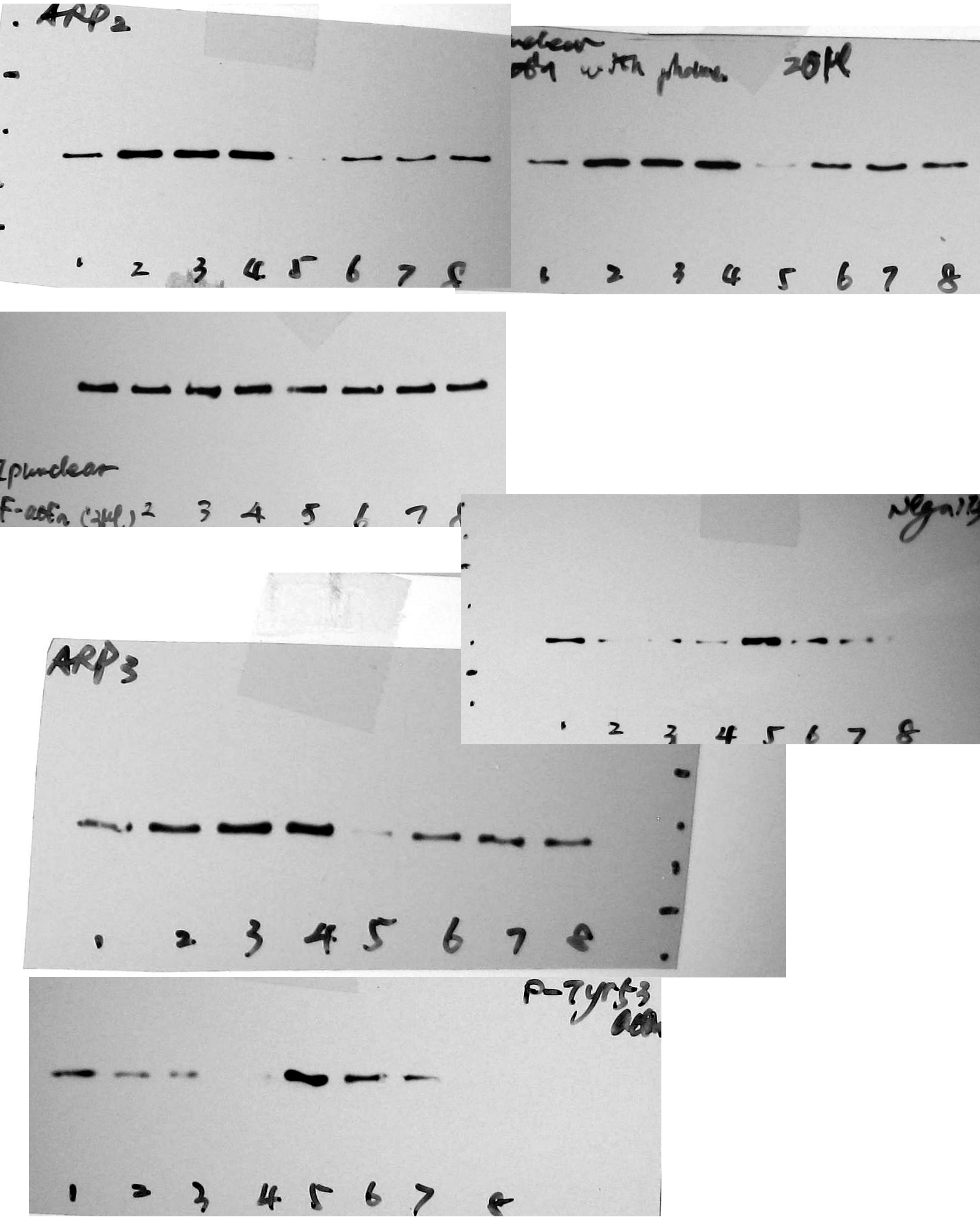
ARP2
20μl
1 2 3 4 5 6 7 8
1 2 3 4 5 6 7 8
1 2 3 4 5 6 7 8
ARP3
1 2 3 4 5 6 7 8
1 2 3 4 5 6 7 8

Fig 9E

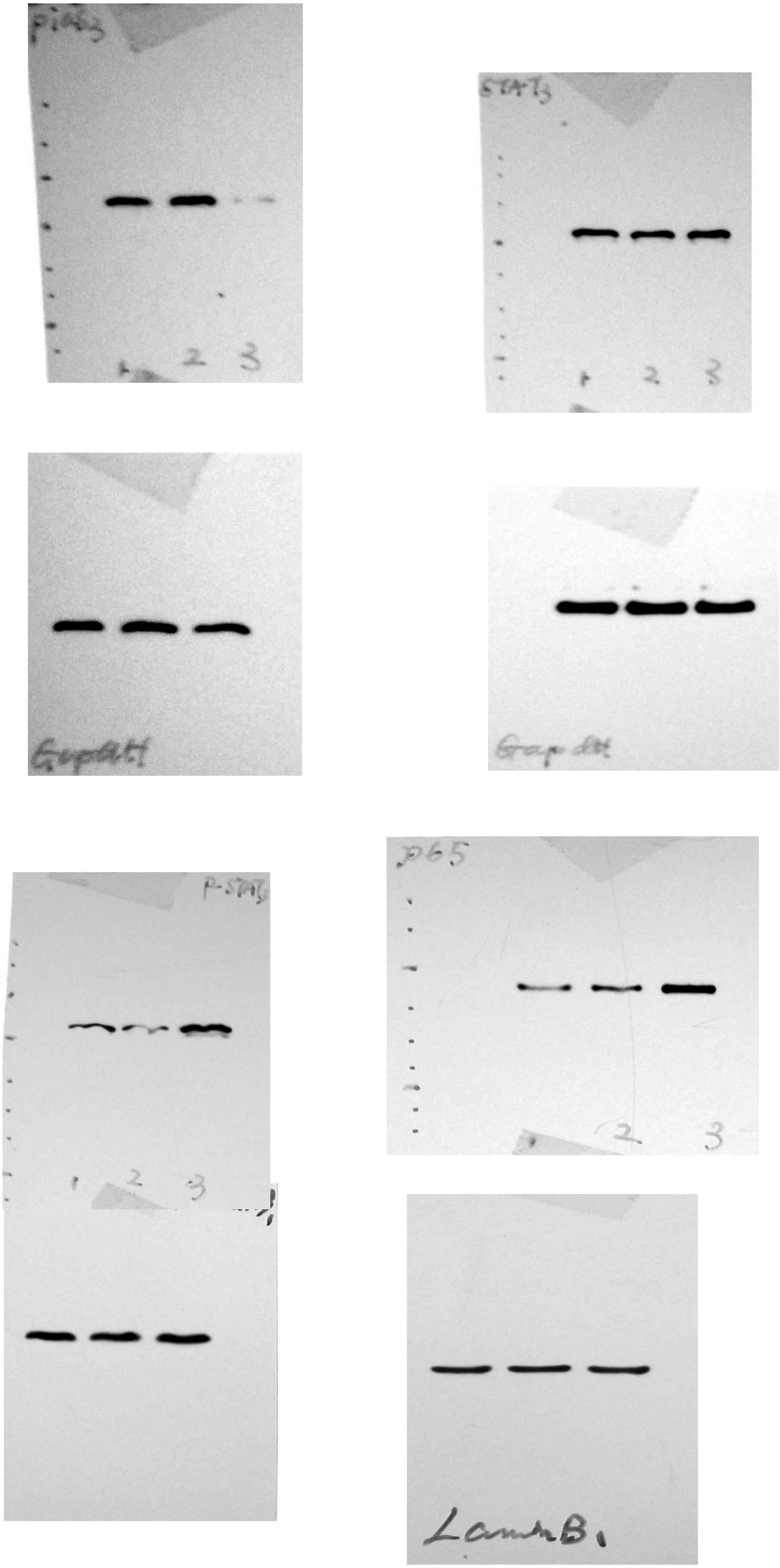

Fig S8E

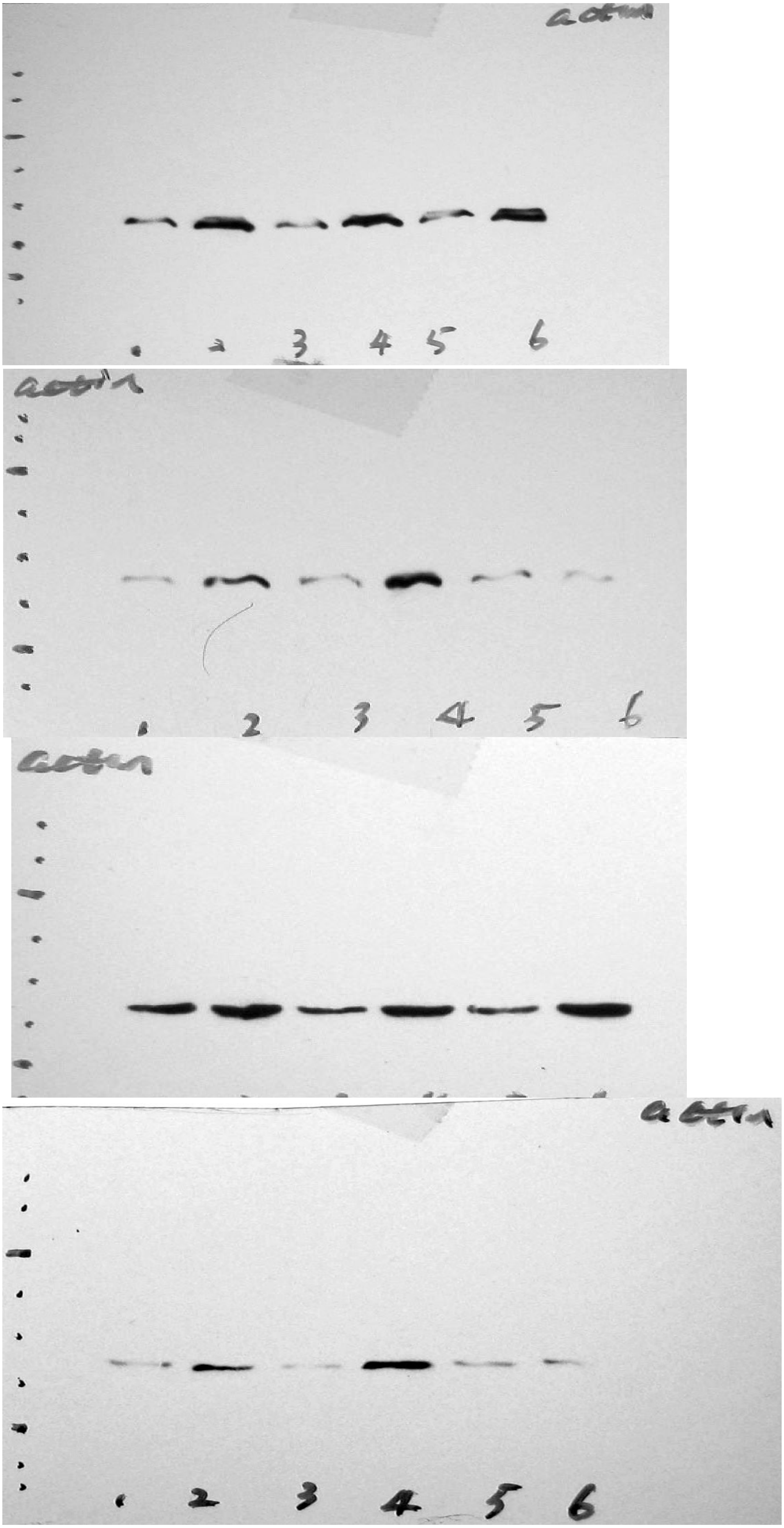

Fig S12C

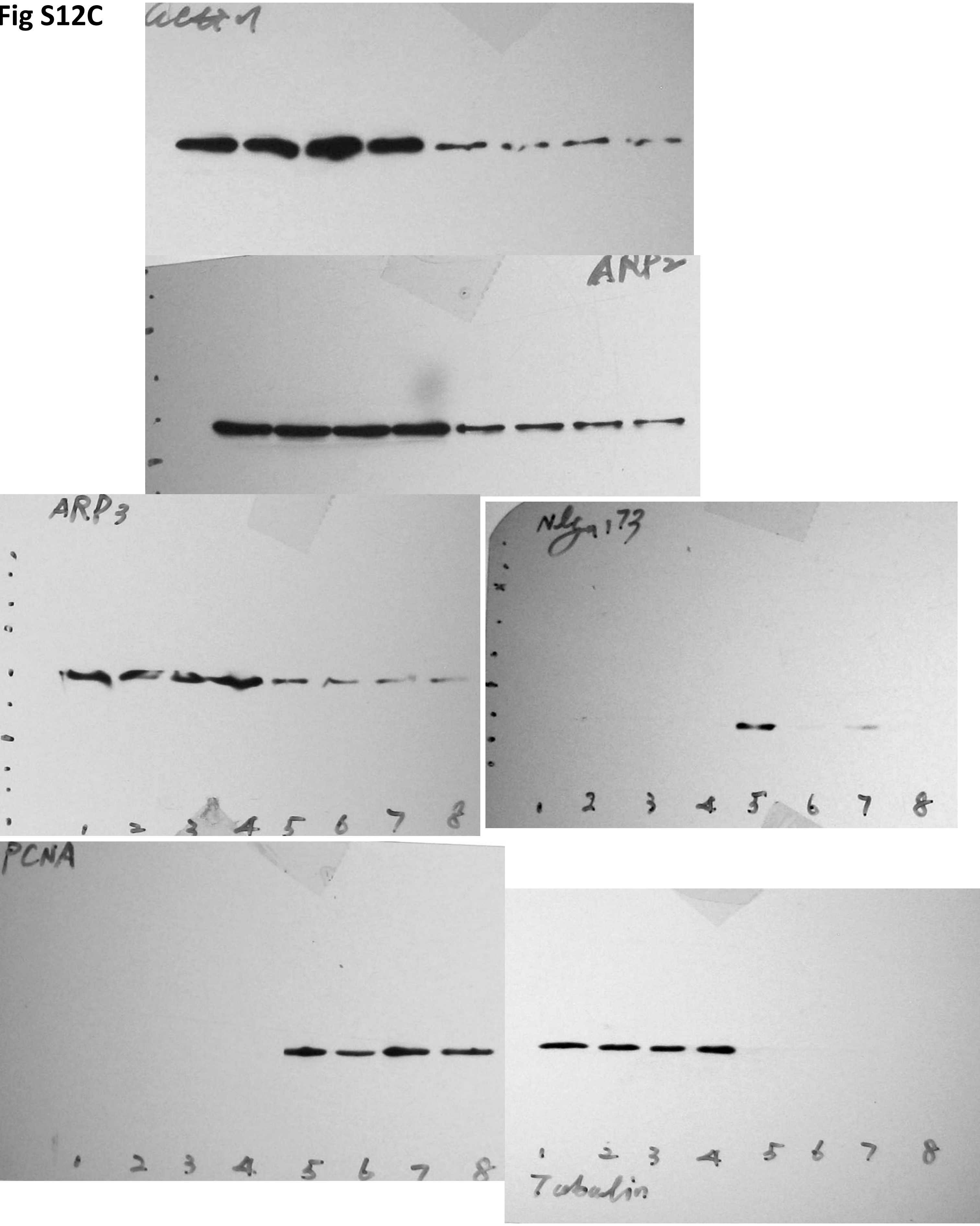

Fig S12D

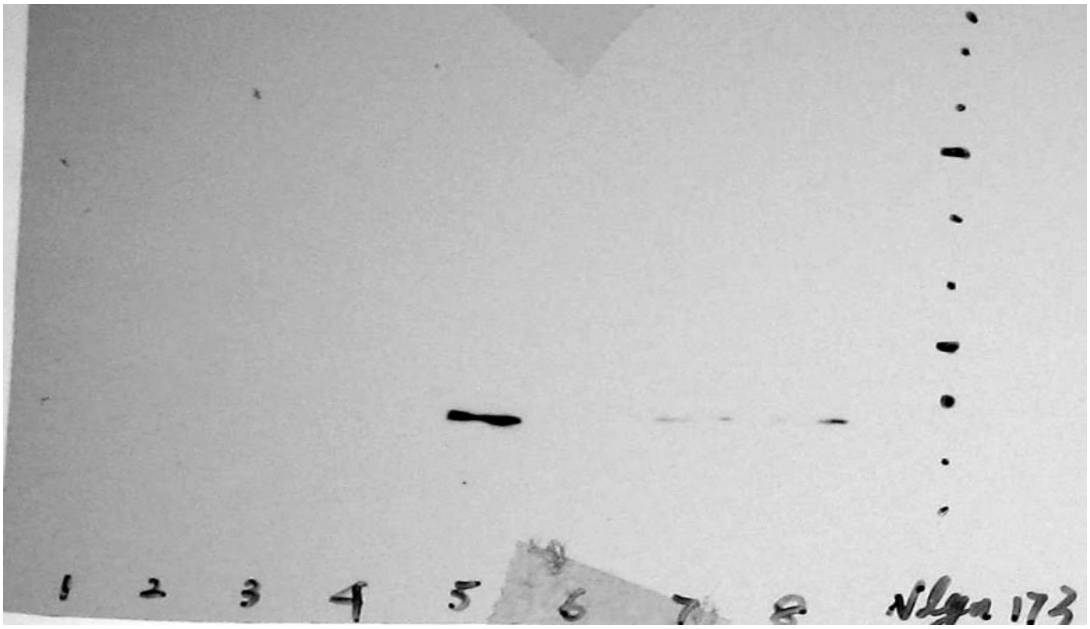

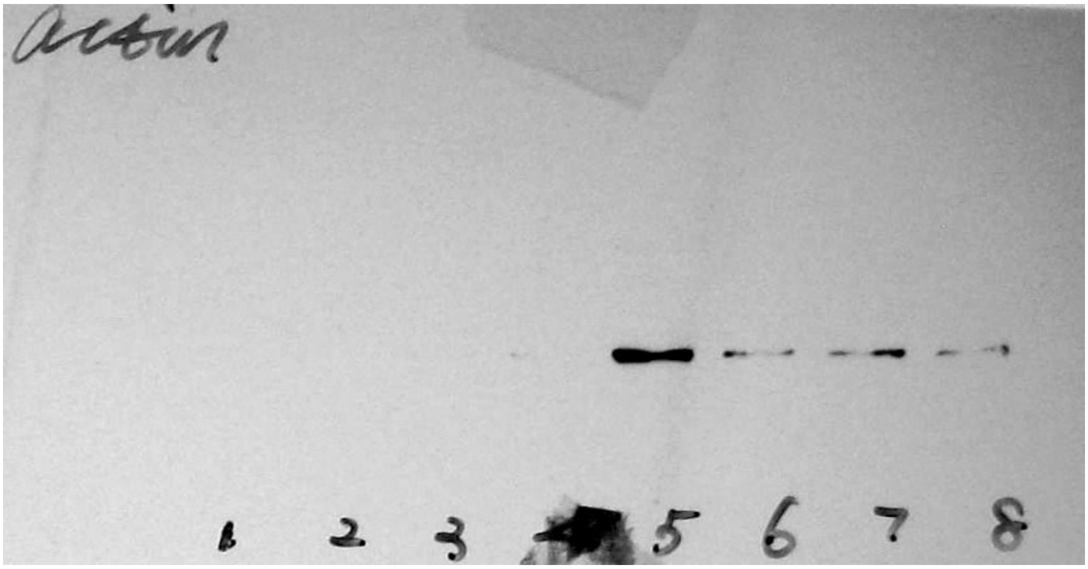

Fig S12E

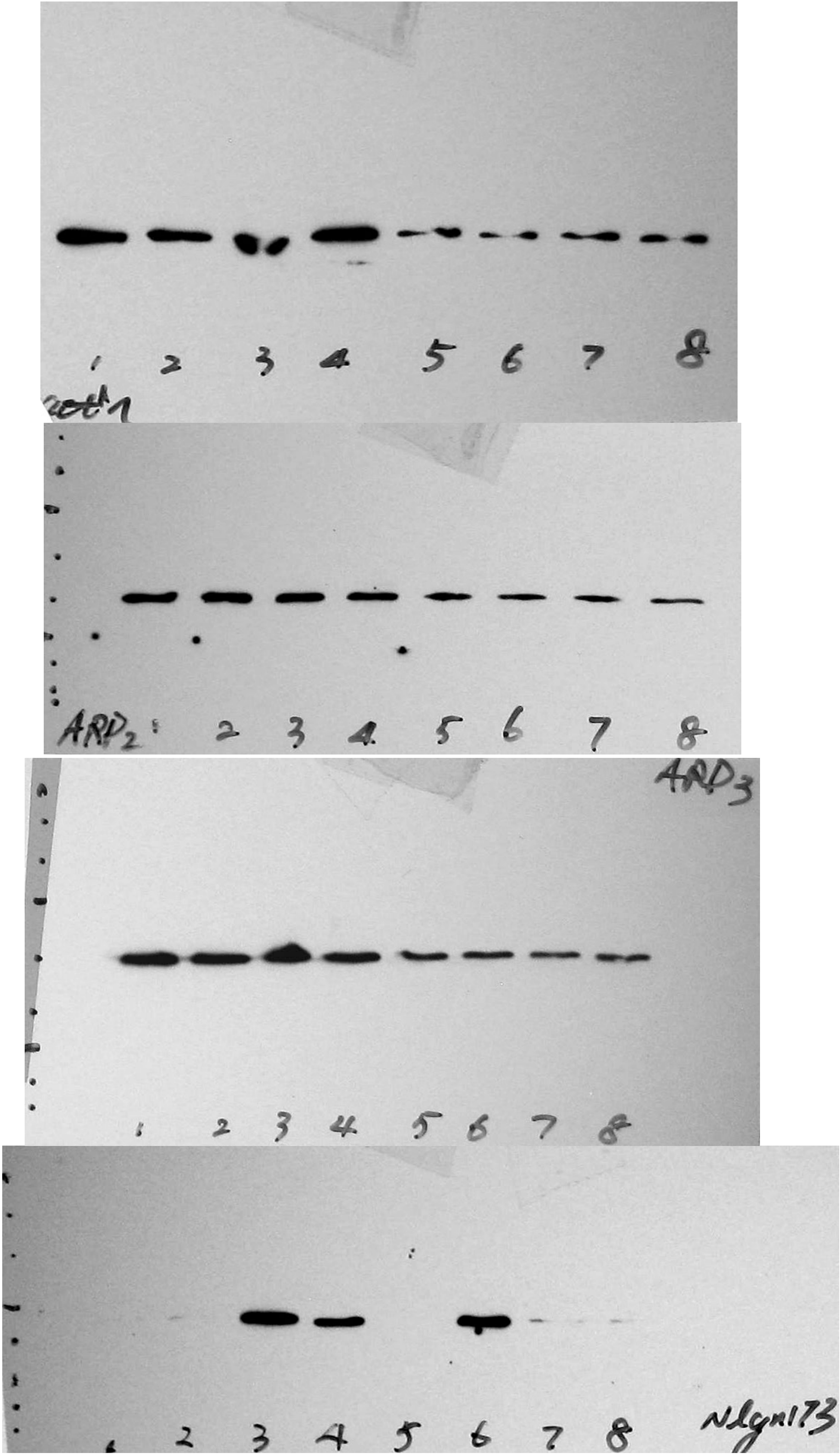

Fig S12F

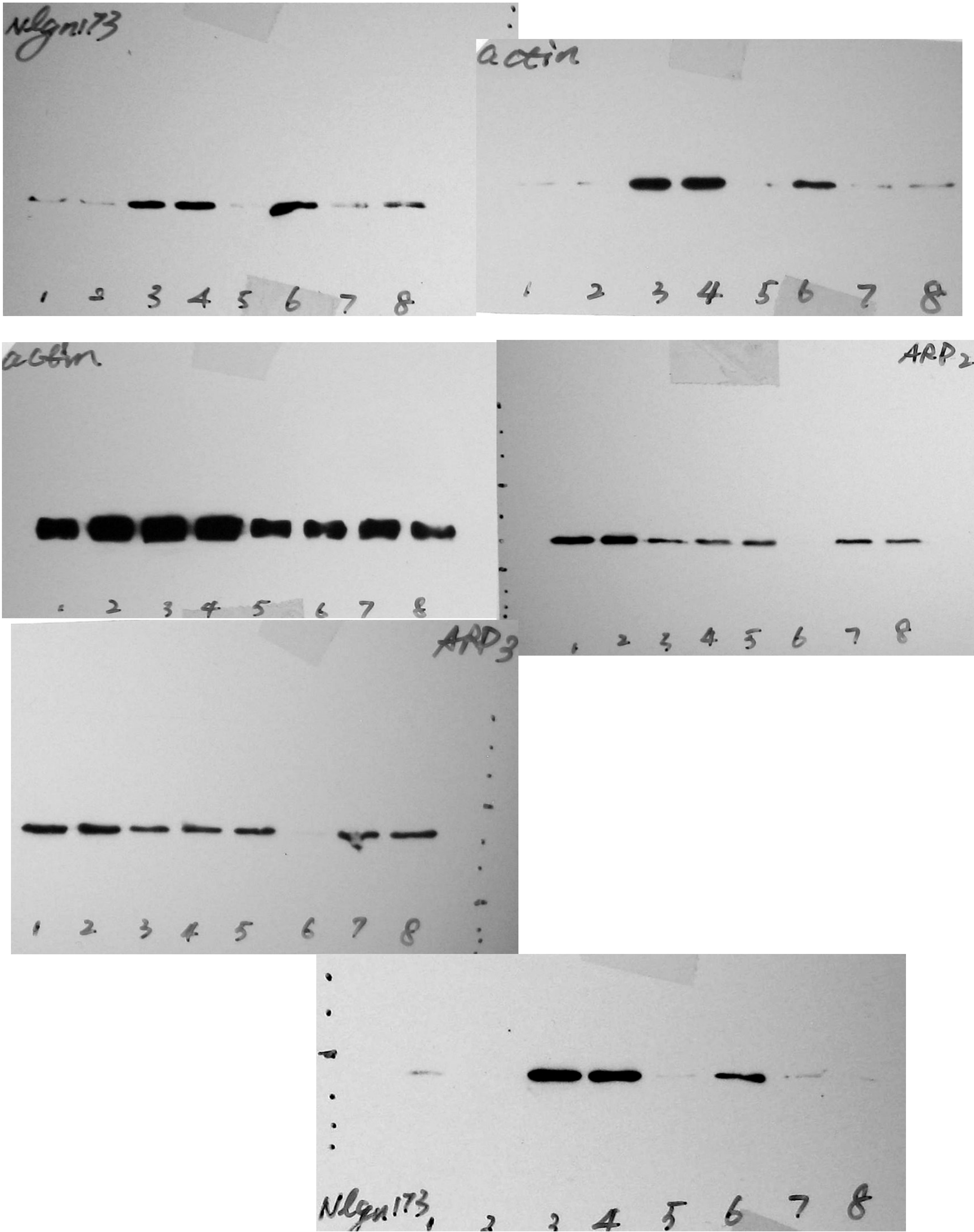

Fig S12F

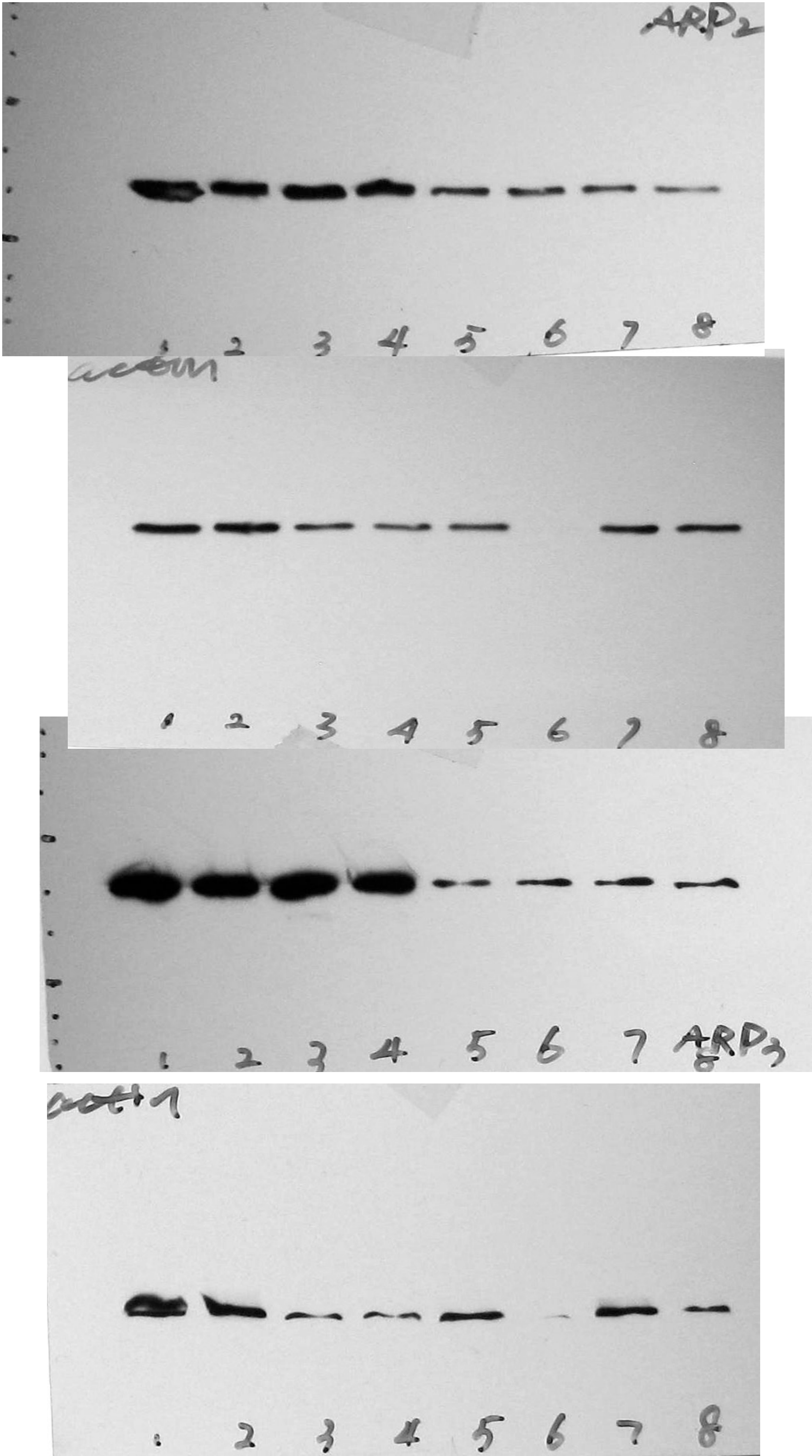

Fig S15D

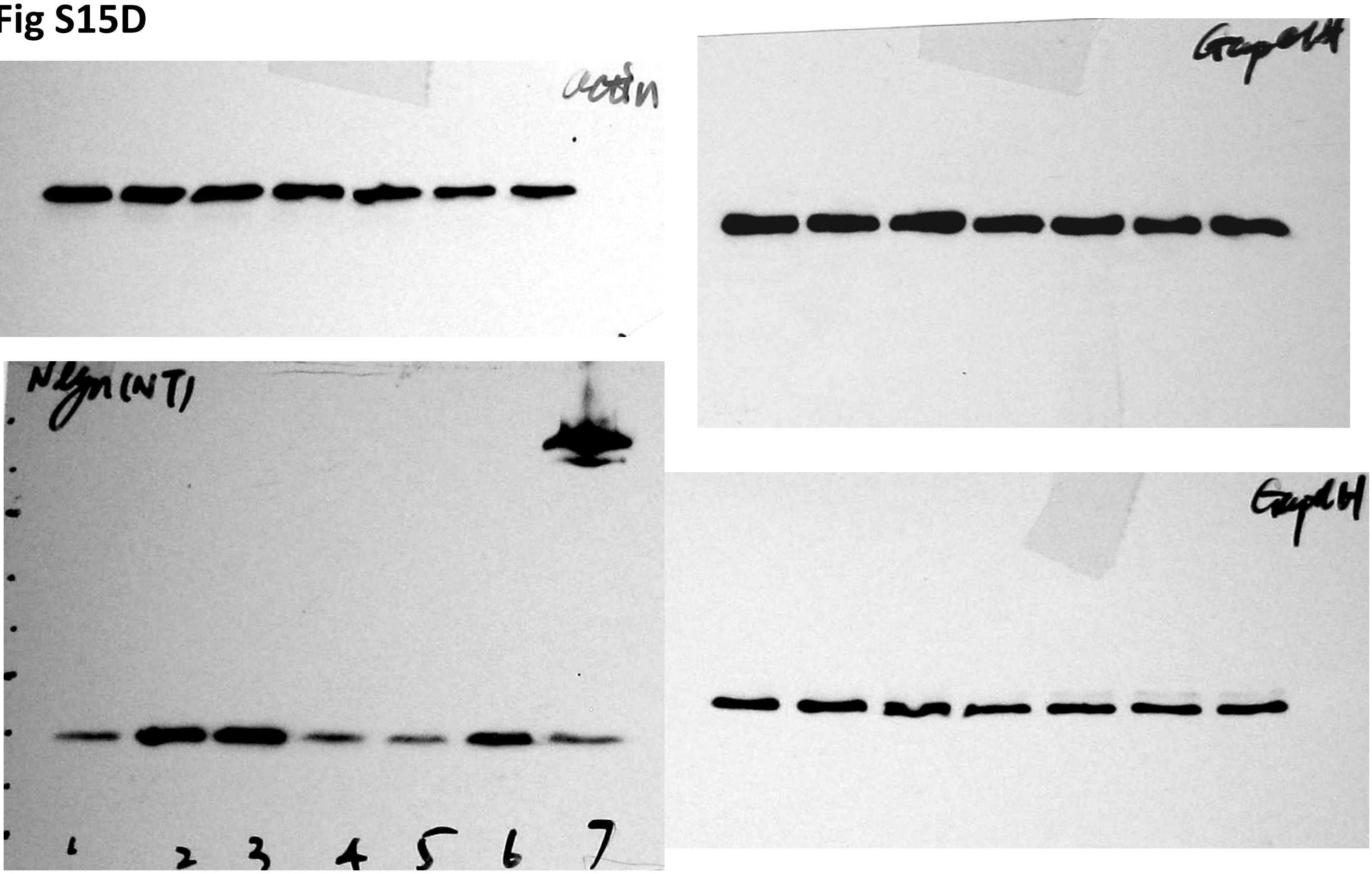

Fig S15E

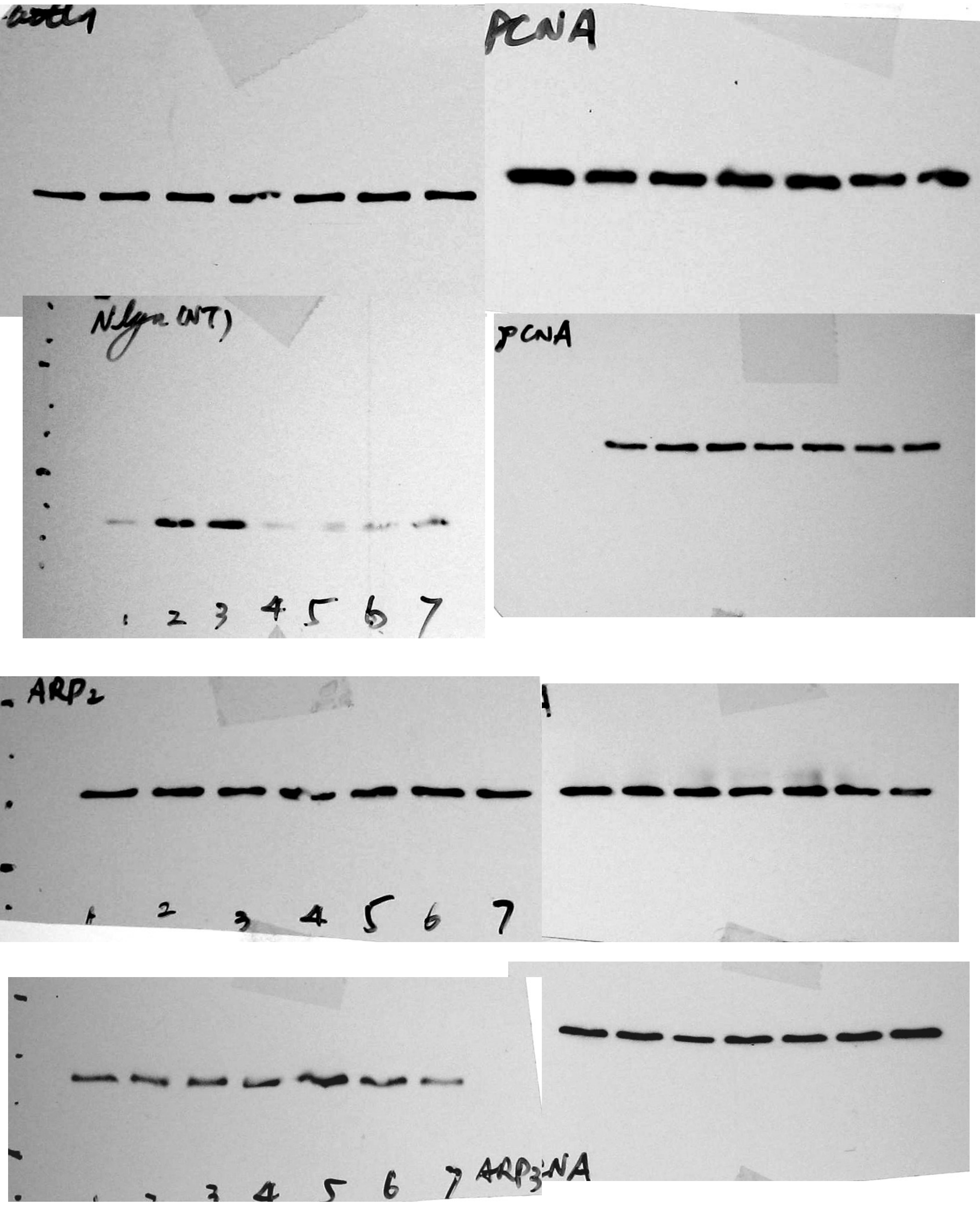

Fig S17B

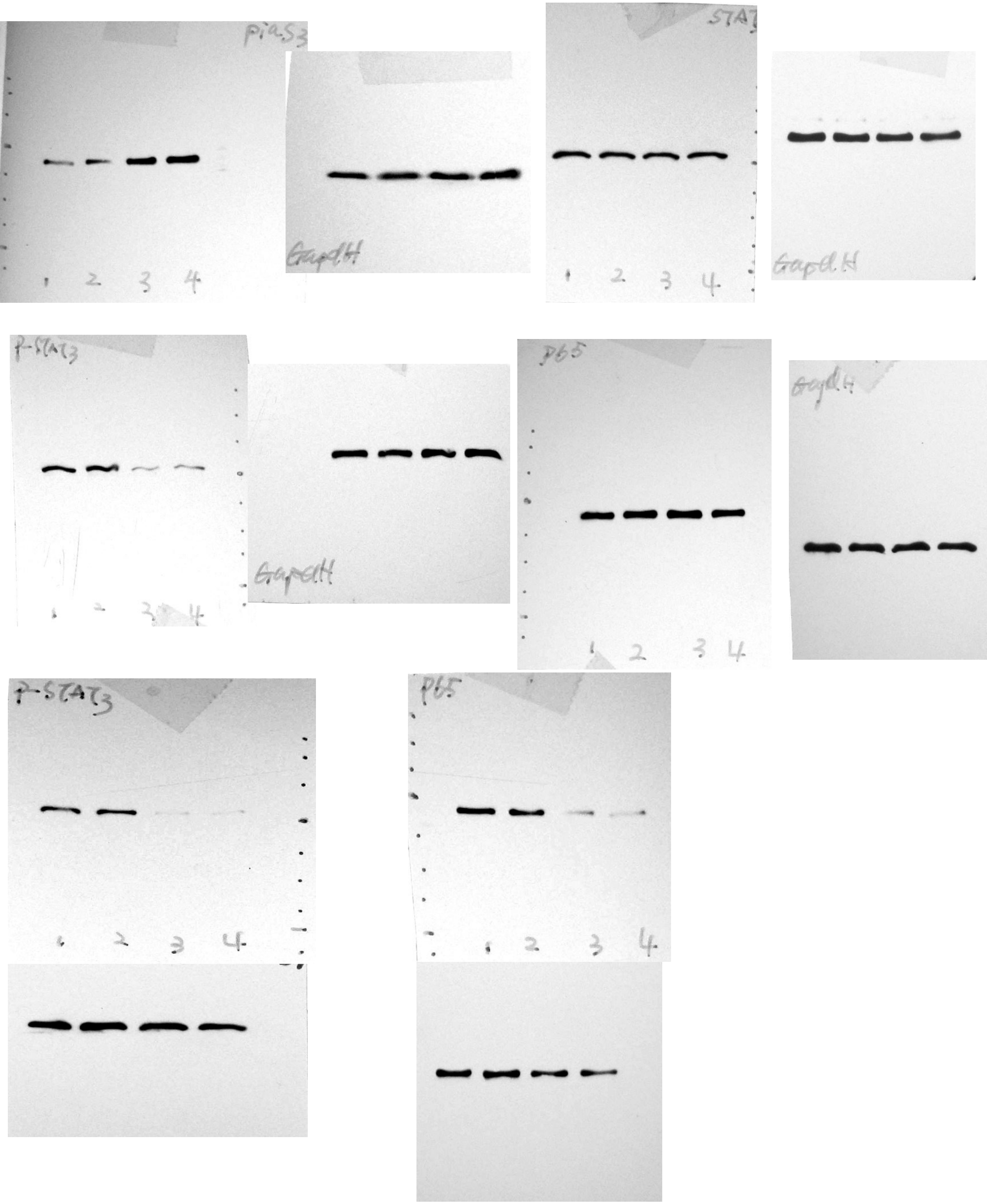
pias3
GapdH
STAT3
GapdH
1 2 3 4
p-STAT3
GapdH
p65
GapdH
p-STAT3
p65
